# National strategy for palliative care of severely ill and dying people and their relatives in pandemics (PallPan) in Germany - study protocol of a mixed-methods project

**DOI:** 10.1186/s12904-021-00898-w

**Published:** 2022-01-13

**Authors:** C. Bausewein, F. Hodiamont, N. Berges, A. Ullrich, C. Gerlach, K. Oechsle, B. Pauli, J. Weber, S. Stiel, N. Schneider, N. Krumm, R. Rolke, C. Gebel, M. Jansky, F. Nauck, U. Wedding, B. van Oorschot, C. Roch, L. Werner, M. Fischer, M. Schallenburger, M. C. Reuters, J. Schwartz, M. Neukirchen, A. Gülay, K. Maus, B. Jaspers, L. Radbruch, M. Heckel, I. Klinger, C. Ostgathe, U. Kriesen, C. Junghanß, E. Lehmann, D. Gesell, S. Gauder, C. Boehlke, G. Becker, A. Pralong, J. Strupp, C. Leisse, K. Schloesser, R. Voltz, N. Jung, S. T. Simon, for the PallPan Study Group

**Affiliations:** 1 Department of Palliative Medicine, Ludwig-Maximilians-University Munich, Munich University Hospital, Munich, Germany; 2 Palliative Care Unit, Department of Oncology, Hematology and Bone Marrow Transplant, University Medical Center Hamburg-Eppendorf, Hamburg, Germany; 3 Department of Palliative Medicine, University of Cologne, Faculty of Medicine and University Hospital, Cologne, Germany; 4 Hannover Medical School, Institute for General Practice and Palliative Medicine, Palliative Care Reserch Grouph Group, Hannover, Germany; 5 Department of Palliative Medicine, University Hospital Aachen, Aachen, Germany; 6 Department of Palliative Medicine, Jena University Hospital, Jena, Germany; 7 Department for Palliative Medicine, Georg August University Goettingen, University Medical Center, Goettingen, Germany; 8 Interdisciplinary Centre for Palliative Medicine, University Wuerzburg, University Hospital, Wuerzburg, Germany; 9 Interdisciplinary Centre for Palliative Care, Heinrich Heine University Duesseldorf, University Hospital, Duesseldorf, Germany; 10 Department of Anesthesiology, Heinrich Heine University Duesseldorf, University Hospital, Duesseldorf, Germany; 11 Department of Palliative Medicine, University Hospital Bonn, Bonn, Germany; 12 Department of Palliative Medicine, Comprehensive Cancer Center, CCC Erlangen – EMN, Friedrich-Alexander-Universität Erlangen-Nürnberg (FAU), University Hospital Erlangen, Erlangen, Germany; 13 Department of Medicine, Clinic III-Hematology, Oncology, Palliative Medicine - CCC Mecklenburg-Vorpommern, Rostock University Medical Center, Rostock, Germany; 14 Faculty of Medicine, Medical Center, Department of Palliative Medicine, University of Freiburg, Freiburg, Germany; 15 Faculty of Medicine and University Hospital, Department I of Internal Medicine, University of Cologne, Cologne, Germany; 16 Faculty of Medicine and University Hospital, Center for Integrated Oncology Aachen Bonn Cologne Dusseldorf (CIO ABCD), University of Cologne, Cologne, Germany; 17 Faculty of Medicine and University Hospital, Clinical Trials Center (ZKS), University of Cologne, Cologne, Germany; 18 Faculty of Medicine and University Hospital, Center for Health Services Research, University of Cologne, Cologne, Germany

**Keywords:** Palliative care, End of life care, SARS-CoV-2, Pandemic, Pandemic preparedness

## Abstract

**Background:**

In the SARS-CoV-2 pandemic, general and specialist Palliative Care (PC) plays an essential role in health care, contributing to symptom control, psycho-social support, and providing support in complex decision making. Numbers of COVID-19 related deaths have recently increased demanding more palliative care input. Also, the pandemic impacts on palliative care for non-COVID-19 patients. Strategies on the care for seriously ill and dying people in pandemic times are lacking. Therefore, the program ‘Palliative care in Pandemics’ (PallPan) aims to develop and consent a national pandemic plan for the care of seriously ill and dying adults and their informal carers in pandemics including (a) guidance for generalist and specialist palliative care of patients with and without SARS-CoV-2 infections on the micro, meso and macro level, (b) collection and development of information material for an online platform, and (c) identification of variables and research questions on palliative care in pandemics for the national pandemic cohort network (NAPKON).

**Methods:**

Mixed-methods project including ten work packages conducting (online) surveys and qualitative interviews to explore and describe i) experiences and burden of patients (with/without SARS-CoV-2 infection) and their relatives, ii) experiences, challenges and potential solutions of health care professionals, stakeholders and decision makers during the SARS-CoV-2 pandemic. The work package results inform the development of a consensus-based guidance. In addition, best practice examples and relevant literature will be collected and variables for data collection identified.

**Discussion:**

For a future “pandemic preparedness” national and international recommendations and concepts for the care of severely ill and dying people are necessary considering both generalist and specialist palliative care in the home care and inpatient setting.

**Supplementary Information:**

The online version contains supplementary material available at 10.1186/s12904-021-00898-w.

## Background

The SARS-CoV-2 pandemic presents a huge challenge for societies and health care systems around the world. The primary aim during the pandemic is the reduction of new infections and the improvement of medical care of COVID-19 patients. The activities within health care systems focus primarily on vaccination and the provision of intensive care beds and respirators for acutely ill COVID-19 patients. At the same time, usual medical care of patients not infected with SARS-CoV-2 needs to be maintained. Despite all efforts, patients will die from COVID-19 but also from other diseases such as cancer or other chronic conditions. Furthermore, protection and rigorous isolation measures and bans on visits may have a severe impact on the situation of seriously ill and dying people and their relatives [1, 2]. A strict lockdown with social isolation leads to patients dying lonely in hospitals and nursing homes without their loved ones, and this not only severely diminishes the quality of dying but also produces high burden on family members with increased risk of post traumatic grief disorders. Even though the development in Germany has been significantly weaker in the first wave compared to other European countries, until February 2021 more than 60,000 people died from or with COVID-19 in Germany. In addition, deficits in care of non-COVID-19 patients have been described (“Corona Collateral Damage Syndrome”) [3].

Hospice and palliative care intends to relief suffering and increase quality of life in patients with life limiting disease at the end of life and is now integral part of many health care systems [4]. The German Association for Palliative Medicine (DGP) published recommendations for symptom relief [5] and for the support of burdened, seriously ill, dying and grieving people in the SARS-CoV-2 pandemic from a palliative care perspective in April 2020 [1]. There have also been a number of international publications on palliative care in pandemic times [2, 6–10]. Reported experiences from patients, general practitioners and nursing homes, but also from palliative care providers show that both the identification of individual preferences and the quality of care for seriously ill and dying patients varies greatly [11, 12].

To be better prepared for future pandemic situations, national and international recommendations and concepts are needed which focus on both general and specialist community and inpatient palliative care. This includes all areas where seriously ill and dying people are cared for.

The Network University Medicine (www.netzwerk-universitaetsmedizin.de) was founded by the Charité Berlin and the German Government as response to the current SARS-CoV-2 pandemic. It is funded by the German Ministry of Education and Research. The Network aims to connect German Medical Schools and German University Hospitals to jointly develop a pandemic preparedness for the current as well as for future pandemic situations. Within this network, 13 large-scale projects were funded focussing on various areas such as surveillance, immunity, data exchange, coordination of mobile pandemic apps, evidence based pandemic management etc. One of the projects is focussing on Palliative Care in Pandemics, called PallPan. The overall aim of PallPan is to develop and consent a national pandemic plan for the care of seriously ill and dying adults and their informal carers in pandemics including (a) guidance for generalist and specialist palliative care of patients with and without (SARS-CoV-2) infections on the micro-, meso- and macro level, (b) collection and development of information material for an online platform planned by the Network University Medicine, and (c) identification of variables on palliative care in pandemics which can be included in the planned national pandemic cohort.

## Methods

### Design

This mixed-methods project combines qualitative methods with semi-structured interviews and focus groups, quantitative methods with online surveys, scoping of the literature, and development of evidence and consensus-based guidance. The project group consists of a consortium of thirteen palliative care departments at German university hospitals: Aachen, Bonn, Cologne, Düsseldorf, Erlangen, Freiburg, Göttingen, Hamburg, Hannover, Jena, Munich, Rostock, and Würzburg. Additional partners come from the university hospitals of Cologne, Mainz, and the Lung Hospital Heckeshorn in Berlin. The group involves researchers from a medical, nursing, sociology, health services research and public health background with expertise in a wide range of quantitative and qualitative research methods.

### Setting

The following settings and stakeholders were chosen on the micro, meso and macro level both for reviewing the experiences of the SARS-CoV-2 pandemic in Germany up to winter 2020/2021 and as recipient for the guidance on palliative care for patients in pandemics (see Table 1).

**Table 1:** Macro, meso and micro level health care setting and authorities

Level	Setting
**MACRO**	Institutions of public administration at federal level with respect to health care: • Federal Government • Federal Ministry of the Interior, for Construction and Home Affairs (BMI) • Federal Ministry of Health (BMG) as well as the joint pandemic response teams of the BMI and BMG
**MESO**	Institutions of public administration at state and community level with respect to health care: • State governments • State Ministries of Health • State and district administrations • City councils • Municipal administrations as well as the executive offices connected to them, such as health offices, regulatory offices, and their pandemic response teams
**MICRO**	Patient care facilities and services (including inhouse pandemic response teams): • Hospitals and nursing homes as well as their providers • Home care services • Inpatient hospices • Palliative care units • Specialist palliative home care teams • Palliative care hospital support teams • General practitioners

### Procedures

**Fig. 1:**
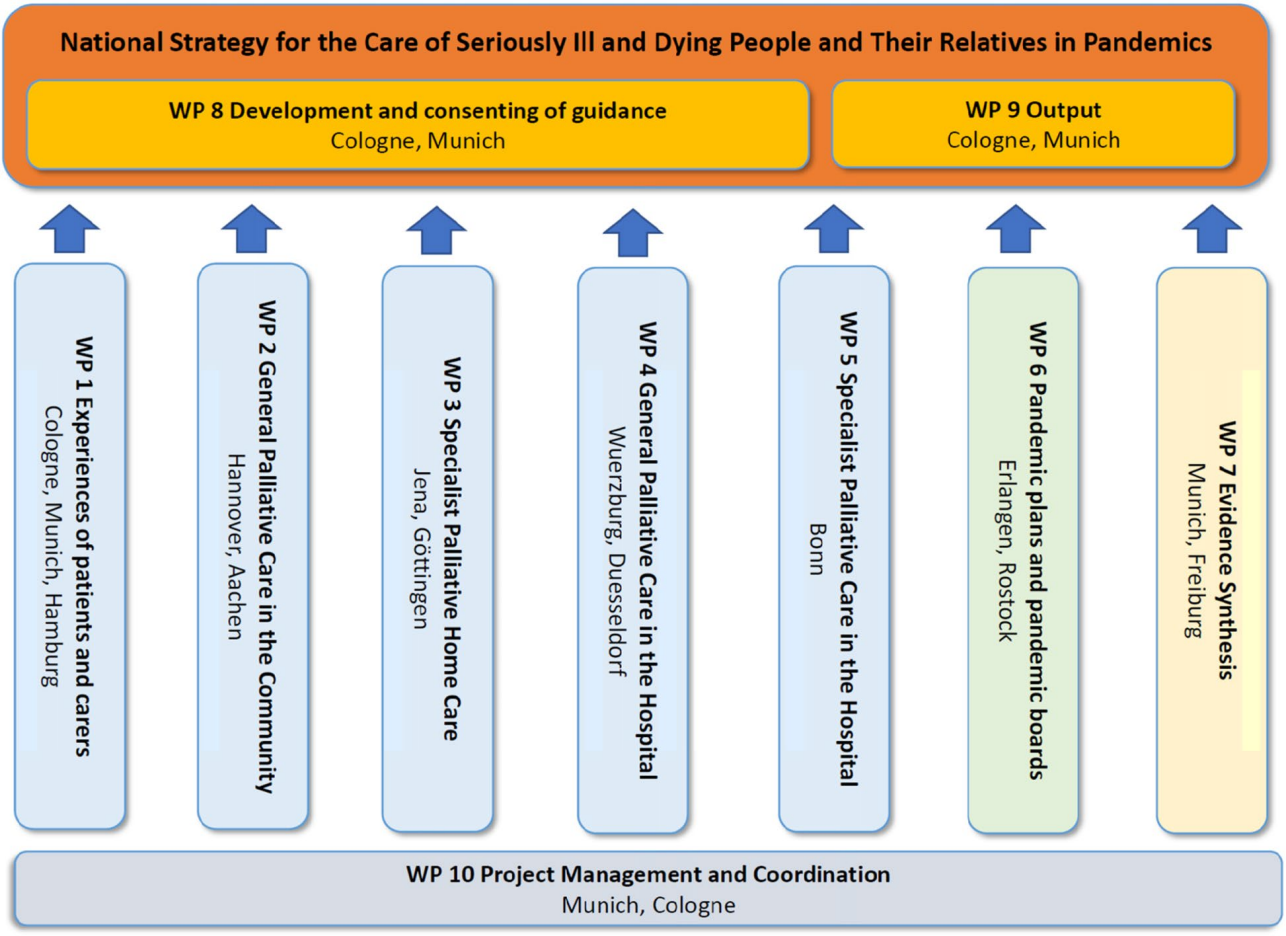
Structure of work packages in the PallPan Project

The project encompasses ten work packages working in parallel. Work packages 1–6 intend to collect and review experiences and practices of the pandemic in various settings and with various players in Germany up to Winter 2020/2021. Work package 7 conducts a scoping review of the current literature. In work package 8, guidance for palliative care in pandemic times is developed and work package 9 collects best practice examples and identifies variables for research data collection (see Fig. 1). Details of the work packages are provided further below.

#### Work package 1: experiences of patients and carers

##### Aim

To explore and describe the experiences of patients and relatives during the SARS-CoV-2 pandemic.

##### Method and design:

i) qualitative semi-structured interviews following an interview guide (Supplementary File WP1 Interview Guide Patients; Supplementary File WP1 Interview Guide Relatives); ii) national online survey based on an abbreviated version of the International Care of the Dying Evaluation (iCODE) questionnaire and the Positive and Negative Affect Schedule (PANAS; free of licence) [13–17].

##### Study population:

i) patients in specialist palliative care (palliative care unit, specialist palliative home care) not infected with SARS-CoV-2 and relatives. ii) Relatives of patients who died during the SARS-CoV-2 pandemic (with and without SARS-CoV-2 infection).

##### Data collection:

i) interviews will be conducted in the setting preferred by patients and relatives, audio recorded and transcribed verbatim, ii) recruitment using a comprehensive approach by clinicians, support groups and public media; data collection using a survey database (LimeSurvey).

##### Data analyses:

i) qualitative data will be analysed inductively following the Framework Method [18]. The software MAXQDA will facilitate data management and content analysis; ii) quantitative data analyses using descriptive statistics (i.e. means, standard deviations, medians, minimum–maximum ranges) and absolute and percentage frequencies using SPSS Statistics 27. German Clinical Trials Register: DRKS00023552 & DRKS00023839.

#### Work package 2: generalist palliative care in the community

##### Aim

To describe experiences, challenges and potential solutions of i) general practitioners (GPs), ii) oncologists, iii) mobile care services and iv) residential nursing homes and facilities for integration assistance in the current pandemic situation with regard to the care of seriously ill and dying patients (with and without SARS-CoV-2 infection) and their relatives.

##### Method and design:

i) Development and application of a German- wide, structured online survey by means of preparatory interviews and focus groups with GPs (Supplementary File WP2 Online Survey Oncologists); ii, iii and iv) qualitative interviews following a semi-structured interview guide (Supplementary File WP2 Interview guide Mobile care services, Supplementary File WP2 Interview guide Oncologists).

##### Study population:

i) GPs working in the community, ii) oncologists working in outpatient practices in the community, iii) mobile care services and iv) residential nursing homes and facilities for integration assistance in Germany.

##### Data collection:

i) preparatory single interviews and focus groups with audio recording and transcription, survey assessment via Questback (/www.questback.com), ii, iii and iv) qualitative interviews following semi-structured interview guide with audio recording and verbatim transcription.

##### Data analyses:

i) content analysis of qualitative data with the software MAXQDA, descriptive statistics (i.e. means, standard deviations, medians, minimum–maximum ranges) and absolute and percentage frequencies with SPSS Statistics 25, ii, iii and iv) content analysis of qualitative data with MAXQDA. German Clinical Trials Register: DRKS00024990.

#### Work package 3: specialist palliative home care

##### Aim

To describe and analyse experiences, challenges and potential solutions of specialist palliative home care teams and volunteer hospice services in the present pandemic with regard to the care of seriously ill and dying patients (with and without SARS-CoV-2 infection) and their relatives and to cooperation with other professionals involved (i.e. nursing services, inpatient hospices, general practitioners etc.).

##### Method and design:

i) Qualitative online focus groups, qualitative telephone/video interviews with representatives of specialist palliative home care federal state associations, and coordinators of volunteer hospice services (Supplementary File WP3 Interview Guide SPHC). ii) quantitative online questionnaire (Supplementary File WP3 Online Survey SPHC).

##### Study population:

Professionals from specialist palliative home care teams, representatives of specialist palliative home care federal state associations and staff from volunteer hospice services.

##### Data collection:

i) 4–6 focus groups with 5–7 participants each from differently affected pandemic areas, 5 representatives of specialist palliative home care state associations and 5 coordinators of volunteer hospice services, ii) nationwide recruitment of executive professionals of specialist palliative home care teams via telephone and e-mail.

##### Data analyses:

i) Content analysis [19] of qualitative data using MAXQDA, ii) descriptive statistics (i.e. means, standard deviations, medians, minimum–maximum ranges) and absolute and percentage frequencies with SPSS Statistics 26, quantitative content analysis for free-text answers. German Clinical Trials Register: DRKS00025090.

#### Work package 4: generalist palliative care in the hospital

##### Aim

To describe and analyse the experience, challenges and potential solutions of hospital staff in generalist palliative care in primary, secondary and tertiary care hospitals with regard to care of seriously ill and dying patients (with and without SARS-CoV-2) and their relatives respectively their bereaved.

##### Method and design:

i) Qualitative semi-structured online focus groups, if necessary additional individual interviews (Supplementary File WP4 Interview guide PC Hospital) ii) literature and expert-based online survey (Supplementary File WP4 Online Survey PC Hospital).

##### Study population:

physicians, nurses and other professions caring for patients with and without SARS-CoV-2 in hospitals (exclusive palliative care units).

##### Data collection:

i) 5 focus groups with 4–8 participants and 1 additional individual interview; recording of the online focus groups using Cisco Webex and transcription ii) nationwide recruitment of participants via mailing lists; online survey using Unipark.

##### Data analysis:

i) qualitative content analysis by Kuckartz [20], ii) descriptive statistics (i.e. means, standard deviations, medians, minimum–maximum ranges) and absolute and percentage frequencies using SPSS Statistics 26, qualitative content analysis by Kuckartz for free text answers. German Clinical Trials Register: DRKS00023595 & DRKS00023591.

#### Work package 5: specialist palliative care in the inpatient setting

##### Aim

To describe and analyse the experiences, challenges and potential solutions of health care professionals working in specialist palliative care services related to the inpatient treatment of patients with and without SARS-CoV-2 infection.

##### Method and design:

i) Secondary data analyses of an online survey on the burden on staff members caused by the pandemic conducted in May 2020 for the subgroup of respondents working in specialist palliative care services, ii) qualitative semi-structured interviews (Supplementary File WP5 Interview guide SPC Inpatient) and iii) an online survey (Supplementary File WP5 Online Survey PC Units; Supplementary File WP5 Online Survey PC Support Teams; Supplementary File WP5 Online Survey Hospices).

##### Study population:

i) health care professionals recruited via different pathways, ii) health care professionals working in palliative care units, palliative care hospital support teams or inpatient hospices identified from the German hospice and palliative care directory and iii) staff members from services identified with purposive sampling.

##### Data collection:

i) secondary evaluation of the database, ii) online survey using ScoSci and iii) semi-structured interviews, either face-to-face or via videoconference with audio recording and verbatim transcription.

##### Data analyses:

i), ii) descriptive statistics (i.e. means, standard deviations, medians, minimum–maximum ranges) and absolute and percentage frequencies using SPSS Statistics 27, iii) content analysis using MaxQDA. German Clinical Trials Register: DRKS00024979.

#### Work package 6: pandemic plans and crisis teams at federal, state, and community level and local health care facilities

##### Aim

To explore SARS-CoV-2 pandemic plans and pandemic boards at federal, state, and community level and local health care facilities with regard to the care of seriously ill patients with far advanced disease at the end of life and their families in the current pandemic.

##### Method and design:

document analysis (pandemic plans), qualitative telephone/video expert interviews (members of pandemic response teams).

##### Study population:

pandemic plans at federal, state and community level, members of pandemic boards (purposefully sampled, considering variance in cases with SARS-CoV-2 infection, settings, expertise, dwelling).

##### Data collection:

systematic collection of pandemic plans, qualitative video or telephone expert interviews with interview guide (Supplementary File WP6 Interview guide Pandemic Teams), recorded, transcribed verbatim.

##### Data analyses and syntheses:

Pandemic plans are analysed in terms of dealing with the seriously ill and dying and their families or caregivers and multiple other aspects. Deductive and inductive content analyses of interviews regarding the composition, organization and authority of pandemic boards with a focus on palliative care expertise and regarding staff experiences on end of life care issues, solutions and challenges. The results of the document analysis and interviews will be angulated with regard to the strategies of the care of seriously ill patients with far advanced disease at the end of life with or without SARS-CoV-2 and their families. German Clinical Trials Register: DRKS00025013.

#### Work package 7: evidence synthesis

##### Aim

i) To identify and synthesise national and international publications on the care of seriously ill patients with far advanced disease at the end of life in pandemic situations; ii) to link „PallPan “to „Ceo-Sys” (COVID-19 evidence ecosystem for improvement of knowledge management and knowledge translation), another consortium project of the Network University Medicine.

##### Method and design:

Scoping review of the literature.

##### Study population:

Seriously ill patients with far advanced disease at the end of life both infected and not infected; informal carers; professionals in the health care system.

##### Data collection:

MEDLINE (PubMed) and websites of international and national palliative care societies are searched in week 52,021 using the search terms “palliative care OR palliative medicine OR residential facilities OR terminal care AND COVID-19 OR corona OR pandemic OR pandemic preparedness”.

##### Data analyses and syntheses:

Deductive and inductive content analyses of all relevant articles following a predeveloped coding frame.

#### Work package 8: development and consenting of guidance

##### Aim

To develop a German guidance for generalist and specialist palliative care of seriously ill and dying patients (with and without infection) and their informal caregivers in pandemic settings with low and high incidence, considering various local and regional disparities of health care in Germany. Addressees are health care professionals in any setting; pandemic boards, stakeholders, and decision-makers in the health care system and on policy level.

##### Method and design:

i) Development of the recommendations by a group of experts, based on the results of the work packages 1–7 and the expertise of the PallPan-Group; ii) Consensus on the recommendations by a multi-professional panel of experts on the micro, meso and macro level, using a modified Group Delphi process with online survey before the workshop [21].

#### Work package 9: outputs

##### Aims

i) To collect information and training materials on palliative care during a pandemic, including best practice examples. ii) To design the “palliative care” area for the platform for pandemics planned by the Network University Medicine (web-based and/or mobile), to provide open access to the collected materials. iii) To define research questions and associated variables for the scientific approach of palliative care in pandemics integrated into the national pandemic cohort (NAPKON; www.napkon.de).

##### Method and Design:

a) Compiling information and training materials by executing a web research and by using networks of the consortium partners. Reviewing, translating and if necessary adapting international experiences and materials to the German healthcare system, including best practice examples, which will be recorded in the other subprojects. b) Testing all outputs for usability (applicability) and designing the “palliative care” area for the information platform of the National Network and adapting outputs for the mobile pandemic app (COMPASS www.netzwerk-universitaetsmedizin.de/projekte/compass). c) Defining the most relevant research questions and associated variables regarding palliative care during a pandemic by extracting results from the other subprojects as well as from completed and ongoing studies (data collections), especially the Lean European Open Survey on SARS-CoV-2 infected patients (LEOSS) project (https://leoss.net/).

#### Work package 10: Project Management and coordination

Two coordinators (CB, STS) are responsible for managing the consortium. The whole group meets in bi-weekly video conferences where regular updates of the work packages are discussed as well as all upcoming issues of the project. In addition, a small group of consortium members meets weekly and develops the guidance supporting work package 8.

### Data management

Research data from qualitative and quantitative surveys of patients, relatives and providers (including pandemic boards and stakeholders) on their experiences from the SARS-CoV-2 pandemic regarding palliative care provision will be collected. The project will generate several different datasets that require the development of a data model sensitive to the different methods of data collection and data coding. The IT infrastructure and data management will be set up by the responsible work package lead. Research data will be collected, secured and stored by each responsible network partner according to existing guidelines.

Participants who do not agree to data handling as described in the informed consent form will not be enrolled into the study.

## Discussion

The SARS-CoV-2 pandemic puts a strain on societies and health care systems demonstrating that worldwide most health care systems are poorly prepared for such a pandemic. Care for seriously ill and dying patients, both infected and not infected, is not in the focus of policy makers. These patients belong to the most vulnerable groups during the pandemic suffering from uncontrolled symptoms, unrelieved psychosocial and existential distress, isolation, and lockdowns. Dying under pandemic conditions also affects informal caregivers and poses significant risk factors for complicated grief, which needs to be prevented and addressed if we want to avoid a post-traumatic pandemic after the pandemic with additional economic consequences [22–24]. Thus, demands for pandemic preparedness need to include the situation of people towards the end of life and their carers irrespective of the underlying pandemic. As palliative care is now integrated in many health care systems it plays a vital role in any humanitarian crises [2, 25, 26]. The PallPan project aims to address these challenges and takes a whole systems approach to develop a national strategy for care of seriously ill and dying people and their relatives during a pandemic, irrespective of the setting or disease. The project unites most palliative medicine university departments in Germany, thus bringing together broad quantitative and qualitative research experience in a collaborative rather than competitive way and is embedded in the Network University Medicine funded by the German Ministry of Education and Research. To ground the national strategy both in most up to date evidence and the literature, several work packages aim to explore and understand the experiences of the current pandemic situation in Germany in all areas of health care provision, not only specialist palliative care. PallPan adds to other national efforts such as the CovPall project (https://www.isrctn.com/ISRCTN16561225) to evaluate the palliative care response in Covid-19.

Although specialist palliative and hospice care are integrated in many hospitals and the home care setting in Germany, there are still gaps in care provision. Therefore, the PallPan project provides a huge opportunity to emphasize the need for high quality care towards the end of life and also the role of generalists in any setting as they provide most of the care of seriously ill and dying people and their relatives. In addition, the project addresses all settings and all seriously ill and dying patient irrespective if infected or not infected in order to get an overall and comprehensive picture. The project will therefore provide guidance for the care of seriously ill and dying patients but will also collect and develop information material and examples for good clinical practice during a pandemic.

There are also some limitations regarding this project. First, the time scale to complete the project is rather short with eight months due to requirement of the funders. Fortunately, an extension of the project was granted until the end of 2021, allowing the project to utilize all the research data collected and having sufficient time to consent the guidance within the national strategy. Second, as the pandemic is quite dynamic with a third wave potentially starting in Spring 2021, it is challenging to capture the whole situation as latest developments might not be integrated in the interview studies and surveys. Nevertheless, the whole PallPan consortium is covering a wide range of expertise and aims to include as much information as possible. Third, including perspectives of other pandemics, e.g., such as Ebola, in the guidance is challenging as we lack this experience in Europe. Nevertheless, the guidance should be adapted for other pandemics as the principles of the care of dying people are the same. In conclusion, the systematic evaluation of the challenges professionals are facing in the current pandemic in health care settings and palliative care specifically will help to establish a better future pandemic preparedness.

## Conclusion

Seriously ill and dying patients and their relatives suffer from additional burden in the current SARS-CoV-2 pandemic. Their care is even more challenging and hindered by protection and rigorous isolation measures and bans on visits. The national PallPan project will provide guidance and supporting material for the care of seriously ill and dying patients and their relatives and will hopefully contribute to better and holistic care at the end of life even in times of pandemics.

## Supplementary Information

**Additional file 1: Supplementary file WP1.** Interview Guide Patients.

**Additional file 2: Supplementary file WP1.** Interview Guide Relatives.

**Additional file 3: Supplementary file WP1.** Interview Online Survey Relatives.

**Additional file 4: Supplementary file WP2.** Interview guide Mobile care services.

**Additional file 5: Supplementary file WP2.** Interview guide Oncologists.

**Additional file 6: Supplementary file WP2.** Online Survey Oncologists.

**Additional file 7: Supplementary file WP3.** Interview Guide SPHC.

**Additional file 8: Supplementary file WP3.** Online Survey SPHC.

**Additional file 9: Supplementary file WP4.** Interview guide PC Hospital.

**Additional file 10: Supplementary file WP4.** Online Survey PC Hospital.

**Additional file 11: Supplementary file WP5.** Interview guide SPC Inpatient.

**Additional file 12: Supplementary file WP5.** Online Survey PC Units.

**Additional file 13: Supplementary file WP5.** Online Survey PC Support Teams.

**Additional file 14: Supplementary file WP5.** Online Survey Inpatient Hospices.

**Additional file 15: Supplementary file WP6.** Interview guide Pandemic Teams.

## Data Availability

Data and materials will be shared on request to the respective work package leads.

## Abbreviations

COMPASS: Coordination on mobile pandemic apps best practice and solution sharing
COVID-19: Coronavirus Disease 2019
DGP: German Association for Palliative Medicine (DGP)
GP: General Practitioner
LEOSS: Lean European Open Survey on SARS-CoV-2 infected patients
MAXQDA: Qualitative Software Package
NAPKON: National Pandemic Cohort Network
SARS-CoV-2: Severe Acute Respiratory Syndrome Coronavirus Type 2
SPSS: Statistical Package for the Social Sciences
