# Supplementary material for: National strategy for palliative care of severely ill and dying people and their relatives in pandemics (PallPan) in Germany - study protocol of a mixed-methods project
Source: BMC Palliat Care. 2022 Jan 13;21:10. doi: 10.1186/s12904-021-00898-w (PMC8756412; doi:10.1186/s12904-021-00898-w)
Supplement: Supplementary file 1 — Additional file 1: Supplementary file WP1. Interview Guide Patients. [file 12904_2021_898_MOESM1_ESM.docx]

**Before the interview**

| Participant information  Space for questions  Informed consent  Explaining the interview  (Continuous text for the ethics application – during the interview free explanation of the points mentioned here) | Thank you very much for agreeing to participate in this study.   \| Before we begin, I’d like to briefly explain the goal of the study again.  These are turbulent times that we live in. We want to use the context of the current situation with COVID-19 to gain an understanding of pallative care patients and their relatives that is as accurate and comprehensive as possible. That means we want to understand what is especially important to you as a patient / you as a relative at the moment and what your concerns or thoughts are.    I will ask some questions about your current situation in a moment. Please tell me about your own views and experiences about it. I am aware that you are in a special phase of life and there are many things around you that are developing dynamically and are changing. So, feel free to share anything you can think of – there are no wrong answers – your current views as well as those of the past weeks/months are welcome.  During the interview, we can always take a break if you want. Also, we can terminate the interview at any point. I will keep an audio recording running during the interview, so we do not lose anything said.  Do you have any questions about this?  *Start of the recording.* \| \| --- \| |
| --- | --- | --- |

**Fulfillment of needs**

| Main question | Adressed? | Potential follow-up questions |
| --- | --- | --- |
| 1. What is currently on your mind regarding your situation?  Alternatively: As a relative, what is currently on your mind regarding your situation? | Current themes + needs | Could you elaborate on that?  What else can you think of? |
| *2. Now, thinking back to the situation before COVID-19, a couple of months ago – that is quite a while:*  How have the topics that concern you changed as a result of the COVID-19 situation? | Change regarding:   - Greater relevance - Reduced relevance   New topics | Which topics became more important for you?  Which topics became less important for you?  Did any new issues arise? If yes, which? |

**Implementation of care**

| Main question | Adressed? | Potential follow-up questions |
| --- | --- | --- |
| 3a. What is your experience of the care you are receiving in this hospital / from the SAPV team?  Alternatively: How are you experiencing the care your mother, father, etc…. is receiving in this hospital / from the SAPV team? | Decision-making ability and sense of control  Communication  Realtionship with doctors/nurses  Change | Which decisions did you have to make in the past few months*? …giving the participant opportunity to reply, then:* How did the COVID-19 situation influence these decisions?  What is your experience regarding the exchange of information / the communication with the treatment team?  In your opinion, which consequences arose from the COVID-19 situation regarding the care? |
| 3b. (additionally for relatives:)  How do you personally feel about that? |  |  |

**Description of fears or worries**

| Main question | Adressed? | Potential follow-up question |
| --- | --- | --- |
| 4. Which worries and fears are you particularly concerned about because of the COVID-19 situation?  5. What is helping you or giving you strength right now? | Social isolation  Uncertainty / certainty  Preparations  Internal resources / coping strategies  External resources | Which protective measures and contact restrictions did you follow?  How did that make you feel? Or rather, which effect did that have on your emotional state?  How did/does the COVID-19 situation influence your attitude toward life?  What plans have you been pursuing in the past months? How did the COVID-19 situation influence these plans?  How do you support yourself in coping with the COVID-19 situation?  Which external sources of support for coping with the COVID-19 situation do you have? |

**Conclusion; space for reflecting on the conversation and asking questions; saying good-bye**

| Is there anything we did not cover that would be important for you in the context of this conversation? |  |
| --- | --- |

**Participant characteristics:**

 Patient  Relative

Context of care:

 Palliative care unit  Palliative care service  Specialist palliative home care

Age: _________________

Gender:

 Female  Male

Marital status:

 Single  In partnership or married
