# Supplementary material for: National strategy for palliative care of severely ill and dying people and their relatives in pandemics (PallPan) in Germany - study protocol of a mixed-methods project
Source: BMC Palliat Care. 2022 Jan 13;21:10. doi: 10.1186/s12904-021-00898-w (PMC8756412; doi:10.1186/s12904-021-00898-w)
Supplement: Supplementary file 2 — Additional file 2: Supplementary file WP1. Interview Guide Relatives. [file 12904_2021_898_MOESM2_ESM.docx]

**Interview guide – relatives**

| 1. **Family members‘ experience of end-of-life care during the COVID-19 pandemic** | |
| --- | --- |
| Experiencing the dying situation- farewell | - How did you experience the accompaniments of your family member's end of life? - Did you have the opportunity to say goodbye before/after death? |
| **Experiencing the pandemic situation**  - Experiences/  Changes  - Challenges/  Worries  - Resources | **Due to the Corona pandemic, the care for many sick people has changed dramatically within a short period.**   - Have you experienced any impact/change in this situation as a result of the Corona pandemic? Can you name examples of the impact? - Have the issues and concerns you are dealing with change as a result of the Corona situation? - What was challenging/stressful for you while caring for your relative during the pandemic situation? - How did you deal with challenges and stresses? What gave you strength? - How do you feel about it today? |
| **Health care in times of pandemic**   - Pos. and neg.   examples   - Measures - Family member’s opinion | - How have you perceived the health care situation since March 2021? - What could the health care (not) provide ? What did they provide additionally / differently? How did this (not) work? Why exactly (not)? What did work well? Please name examples; - What measures/regulations to contain the pandemic have you noticed and what is your attitude towards them? |
| **Specific needs of**  patients  and relatives in times of pandemic | - What additional needs did you and your relative have as a result of the COVID-19 pandemic? - Have these additional needs been addressed? - Possibly further question: decision-making and communication |
| **Solutions**  in times of Pandemic | - What solutions were suggested to overcome thepandamic-related challenges what would you have wished for? - In retrospect, how do you rate these approaches to solve the problem? |
| Perspectives for future pandemic times | Suppose we were facing another pandemic (with COVID or other diseases)...   - What would you wish for other people in a similar situation in terms of future support to feel well taken care of? |
| Further Topics | Is there anything else you would like to tell me and share with me regarding the care of your relative in times of pandemic that I have not asked about yet? |
