# Supplementary material for: National strategy for palliative care of severely ill and dying people and their relatives in pandemics (PallPan) in Germany - study protocol of a mixed-methods project
Source: BMC Palliat Care. 2022 Jan 13;21:10. doi: 10.1186/s12904-021-00898-w (PMC8756412; doi:10.1186/s12904-021-00898-w)
Supplement: Supplementary file 3 — Additional file 3: Supplementary file WP1. Interview Online Survey Relatives. [file 12904_2021_898_MOESM3_ESM.docx]

| **PallPan**  **Workpackage 1: Online Survey Relatives** | |
| --- | --- |
|  | |
| **Your relative**  *In this part, we ask you some general questions about your deceased relative.* | |
| 1. What was the age of your relative? | _________ years |
| 1. Was your relative a man or a woman? | - Man - Women - Other |
| 1. Where did your relative live? | - … - Etc. |
| 1. When did your relative die?   Please fill in the date. | .. /.. /2020 |
| 1. How were you related to your relative? | - S/he was my parent - S/he was my partner - S/he was another family member, namely:   _________________________   - S/he was a friend - Other relationship, namely:   S/he was my _________________________ |
| 1. Was your relative infected with the new Coronavirus? | - Yes, certainly - Yes, probably - No, probably not - No, certainly not - Don’t know |
| 1. Did you relative have (other) health problems?   (more than one answer possible) | - Cancer - Heart disease - Lung disease - Diabetes - Dementia - Other disease, namely:   _________________________   - No, my relative had no (other) disease |

| **Care for your relative** | |
| --- | --- |
| *Please look at the following statements and cross the answer box that corresponds best with your opinion*. | |
| **The care received from the nurses & doctors**  *These questions are concerned with the general care your relative received from the doctors and nurses and, where appropriate the environment in which this care was delivered. The questions apply to the last* ***two days*** *of his/her life and relates to the doctors and nurses (including healthcare assistants and / or care agency staff) who were most involved with his/her care during this time.* | |
| 1. There was enough medical care, from the general practitioner or another doctor. | - Strongly agree - Agree - Neither agree nor disagree - Disagree - Strongly disagree - I don’t know |
| 1. There was enough help with nursing care, such as giving medicines and helping him/her find a comfortable position in bed. | - Strongly agree - Agree - Neither agree nor disagree - Disagree - Strongly disagree - I don’t know |
| 1. There was enough help available to meet his/her personal care needs, such as washing, personal hygiene and toileting needs. | - Strongly agree - Agree - Neither agree nor disagree - Disagree - Strongly disagree - I don’t know |
| 1. The bed area and surrounding environment was comfortable for him/her. | - Strongly agree - Agree - Neither agree nor disagree - Disagree - Strongly disagree - I don’t know |
| 1. The bed area and surrounding environment had adequate privacy for my relative. | - Strongly agree - Agree - Neither agree nor disagree - Disagree - Strongly disagree - I don’t know |
| 1. Did you have confidence and trust in the nurses who were caring for him/her? | - Yes, in all of them - Yes, in some of them - No, not in any of the nurses - Not applicable, there were no nurses caring for my relative |
| 1. Did you have confidence and trust in the doctors who were caring for him/her? | - Yes, in all of them - Yes, in some of them - No, not in any of the doctors - Not applicable, there were no doctors caring for my relative |
| 1. The nurses had time to listen and discuss his/her condition with me. | - Strongly agree - Agree - Neither agree nor disagree - Disagree - Strongly disagree - Not applicable, I have had no contact with nurses |
| 1. The doctors had time to listen and discuss his/her condition with me. | - Strongly agree - Agree - Neither agree nor disagree - Disagree - Strongly disagree - Not applicable, I have had no contact with doctors |
| **The control of pain & other symptoms**  *These questions are concerned with the symptoms s/he had and the care s/he received during the last two days of his/her life.* | |
| 1. In your opinion, during the last two days, did s/he appear to suffer from shortness of breath? | - Yes, all of the time - Yes, some of the time - No, s/he did not seem to suffer from shortness of breath |
| 1. In your view, did the doctors and nurses do enough to help relieve the shortness of breath? | - Yes, all of the time - Yes, some of the time - No, not at all - Not applicable, s/he did not seem to suffer from shortness of breath |
| 1. In your opinion, during the last two days, did s/he appear to be in pain? | - Yes, all of the time - Yes, some of the time - No, s/he did not appear to be in pain |
| 1. In your view, did the doctors and nurses do enough to help relieve the pain? | - Yes, all of the time - Yes, some of the time - No, not at all - Not applicable, s/he was not in pain |
| 1. In your opinion, during the last two days, did s/he appear to be restless? | - Yes, all of the time - Yes, some of the time - No, s/he did not appear to be restless |
| 1. In your view, did the doctors and nurses do enough to help relieve the restlessness? | - Yes, all of the time - Yes, some of the time - No, not at all - Not applicable, s/he was not restless |
| **Communication with the healthcare team**  *The following questions are about the communication that you, your family members and friends received from the healthcare team who were most involved with his/her care in the last two days of his/her life. By ‘healthcare team’, we mean the doctors, the nurses and any other member of staff who may have been involved in his/her care such as a social worker or a chaplain.* | |
| 1. Did the healthcare team explain his/her condition and/or treatment in a way you found easy or difficult to understand? | - Very easy - Fairly easy - Fairly difficult - Very difficult - They did not explain his/her condition or treatment to me |
| 1. Were you able to obtain enough information about the patient's condition from the physicians so that you felt adequately informed? |  Strongly agree   Agree   Neither agree nor disagree   Disagree   Strongly disagree   I don’t know |
| 1. How involved were you with the decisions about his/her care and treatment? | - Very involved - Fairly involved - Not involved |
| 1. Did the healthcare team discuss any limitations in his/her treatment or care? | - Yes, clearly - Yes, but not very clearly - No, not that I know of → go to question 28 - Don’t know → go to question 28 |
| 1. Which limitations were discussed?   (more than one answer possible) | - No resuscitation - No ICU admission - No hospital admission - No admission to other inpatient care facility - Other limitation, namely:   ___________________________________ |
| 1. Did the healthcare team discuss the reason for limiting his/her treatment or care?   (more than one answer possible) | - Yes, namely:   - Full treatment was futile because of his/her medical condition   - It was my relative’s choice   - My relative was too old   - There were not enough beds available   - There was not enough healthcare staff available   - Other reason, namely:   __________________________   - No, the reason was not discussed |
| 1. Do you think that the treatment or care of your relative was limited due to the Corona crisis? | - Yes - No - Don’t know |
| If you wish, you can explain your answer: |  |
| **The emotional & spiritual support provided by the healthcare team**  *The following questions are about the emotional and spiritual support that was provided to you and your family member or friend by the healthcare team in the last two days of his/her life. By ‘spiritual support’, we mean support relating to important personal beliefs. These beliefs may be connected with a specific religion but may also be personal beliefs about what life means, what provided you or your family member / friend with hope and helped you cope.* | |
| 1. How would you assess the overall level of emotional support given to you by the healthcare team? | - Excellent - Good - Fair - Poor |
| 1. Overall, his/her religious or spiritual needs were met by the healthcare team. | - Strongly agree - Agree - Neither agree nor disagree - Disagree - Strongly disagree |
| 1. Overall, my religious or spiritual needs were met by the healthcare team. | - Strongly agree - Agree - Neither agree nor disagree - Disagree - Strongly disagree |
| **The circumstances surrounding his/her death**  *The following questions are about the circumstances surrounding his/her death, and your feelings about the way in which the healthcare team treated you and your family member at this time. By ‘healthcare team’, we mean the doctors, the nurses and any other member of staff who may have been involved in his/her care such as a social worker or a chaplain.* | |
| 1. Before s/he died, were you told s/he was likely to die soon? | - Yes - No |
| 1. Did a member of the healthcare team talk to you about what to expect when s/he was dying (e.g. symptoms that may arise)? | - Yes → go to question 35 - No |
| 1. Would a discussion about what to expect when s/he was dying have been helpful? | - Yes - No - Not applicable, we had these types of discussions |
| 1. Where did s/he die? | - At home - In a hospital:   - At an ICU   - At a ward for Corona patients   - At another ward - In a care home / nursing home   - At a ward for Corona patients   - At another ward - In an institution for Corona patients - In a hospice (not specifically for Corona patients) - Other (please specify) :   _______________________________ |
| 1. Were visitors allowed during the last two days of his/her life?   (more than one answer possible) | - Yes, without limitation - Yes, with limitations:   Maximum number of visitors per day:  _________________   - Yes, with limitations:   Maximum amount of time per visit:  _________________ minutes   - No visitors were allowed |
| 1. Did you feel safe during visits from your relative (if they were allowed)? | - Yes - No - Don´t know |
| 1. Were you trained as a visitor regarding protection and hygiene measures? | - Yes - No - Don´t know |
| 1. If yes, how were you trained? | - Online - In person by experts - In person by persons trained by experts |
| 1. Was this training sufficient for you to feel safe? | - Yes - No - Don´t know |
| 1. Were there enough protection supplies available for you as a relative? | - Yes - No - Don´t know |
| 1. Were there enough protection supplies available for the healthcare team? | - Yes - No - Don´t know |
| 1. Was there an opportunity for relatives to have online contact with the patient (e.g. through Skype, Facetime, etc.)? | - Yes, with facilities provided by the healthcare organisation - Yes, but this was not specifically facilitated by the healthcare organisation - No |
| 1. In case of visiting restrictions, were there alternatives for communication? | - Yes - No |
| 1. If yes: | - Patient via Video - Patient via phone - Doctor via phone (regularly) - Doctor via phone (seldom) - Caregiver via phone |
| 1. Did the patient die alone? | - Yes - No - Don’t know |
| 1. If yes – did the patient die alone due to visiting restrictions? | - Yes - No - Don´t know |
| 1. If no - in presence of | - Relatives - Caregivers / doctors - Visiting service - Other ________ |
| 1. Did the Healthcare Team wear mouth-to-nose protection during their stay? | - Yes - No - Don´t know |
| 1. If yes- did you feel that wearing the mask made communication more difficult? | - Strongly agree - Agree - Neither agree nor disagree - Disagree - Strongly disagree |
| 1. Were there any health care providers (e.g., physical therapy, psychologist, chaplaincy, primary care physician) who did not come any longer due to the pandemic (e.g., primary care physician? | - Yes - No - Don´t know |
| 1. In your opinion did s/he die in the right place? | - Yes, it was the right place - No, it was not the right place - Not sure - Don’t know |
| 1. I was given enough help and support by the healthcare team at the actual time of his/her death. | - Strongly agree - Agree - Neither agree nor disagree - Disagree - Strongly disagree |
| 1. After s/he had died, did individuals from the healthcare team deal with you in a sensitive manner? | - Yes - No - Not applicable, I didn’t have any contact with the healthcare team |
| 1. Was it allowed for relatives to assist in laying out his/her body? | - Yes - No - We did not ask for this - Don’t know |
| 1. Did the SARS COV2 pandemic situation result in any additional pandemic-related stress (in addition to the stress of having a close relative of yours at the end of life dying)? | - Yes - No |
| 1. If yes: What situation (due to the pandemic) has been most stressful to you? | Open-ended |
| 1. What were the main challenges in end-of-life care for the patient due to the pandemic situation? | Open-ended |
| 1. What would you have wished for in the end-of-life care of the patient in a time of pandemic in terms of additional information, options, offers, measures, etc.? | Open-ended |
| 1. Do you have ideas and suggestions on how to better accompany/support people at the end of life and dying phase and their relatives in a pandemic situation? [please name 3 ideas or suggestions] | Open-ended |
| 1. Are you infected with the Corona virus? | - Yes, certainly - Yes, probably - No, probably not - No, certainly not - Don’t know |

| **Support from others** | | | | | |
| --- | --- | --- | --- | --- | --- |
| 1. In the two days before the death of your relative, how often did you experience any of the following? | | | | | |
|  | Never | | Rarely | Sometimes | Often |
| I lacked companionship |  | |  |  |  |
| I felt close to people |  | |  |  |  |
| There were people I could turn to |  | |  |  |  |
| I felt that people really understood me |  | |  |  |  |
| 1. In the days after the death of your relative, how often did you experience any of the following? | | | | | |
|  | Never | | Rarely | Sometimes | Often |
| I lacked companionship |  | |  |  |  |
| I felt close to people |  | |  |  |  |
| There were people I could turn to |  | |  |  |  |
| I felt that people really understood me |  | |  |  |  |
| 1. What is your age? | | _________ years | | | |
| 1. Are you a man or a woman? | | - Man - Women - Other | | | |
