# Supplementary material for: National strategy for palliative care of severely ill and dying people and their relatives in pandemics (PallPan) in Germany - study protocol of a mixed-methods project
Source: BMC Palliat Care. 2022 Jan 13;21:10. doi: 10.1186/s12904-021-00898-w (PMC8756412; doi:10.1186/s12904-021-00898-w)
Supplement: Supplementary file 4 — Additional file 4: Supplementary file WP2. Interview guide Mobile care services. [file 12904_2021_898_MOESM4_ESM.docx]

**WP 2 Interview guide Mobile care services**

| **Before interview** | |
| --- | --- |
| Welcome |  |
| Short introduction | My name is ... I am a member of the “PallPan” project at the Clinic for Palliative Medicine. I had sent you a brief profile of myself. I am very pleased that I am allowed to conduct the interview with you today and that you are taking the time to talk to us. |
| Aim of study | I am very pleased that you are sharing your experiences and challenges from the COVID-19 pandemic and your approaches to solving them with me.  We are particularly interested in your perspective on caring for seriously ill and dying patients (with / without COVID-19) and their relatives during the COVID-19 pandemic.  Your information will help us to get a realistic picture of this special situation in Germany. |
| Explanation of the interview process | I'm going to ask you a few open questions below. You are free to tell me what you think of on the respective topic. Since we are at the very beginning of our work, everything is interesting and important to us. |
| Note regarding Corona | Our questions expressly relate to this specific corona situation compared to standard care in normal times. |
| Detailed descriptions are desirable | Your answers are welcome to be detailed. In order to be able to process your descriptions for us afterwards, I will run an audio recording device during the interview. |
| Consent | *Depending on the situation:*  *You have already received information and a declaration of consent by post*  *- and signed the consent and sent it back to us.*   - *- the consent has not yet been returned. Please do this urgently. Do you still have the form, or should I send it to you again?* |
| Anonymity / data protection | All information you provide will be treated in accordance with the data protection guidelines. This also means that we store all audio recordings and interviews under an anonymous identification number. |
| Inquiries | Do you have any questions in advance? |
| Start audio recording | I will now start the interview and start the audio recording. |

| 1. **Care of seriously ill and dying patients with COVID-19 and their relatives in pandemic times** | |
| --- | --- |
| **Screening** | - **Have you accompanied / cared for patients in palliative situations with COVID-19 on an outpatient basis?** - **If so, let's talk about these patients first.** |
| Experiencing the pandemic situation | - How did you experience these patients and relatives being accompanied? - How did you and your care team feel about it? |
| Experiences and challenges in supply in pandemic times | - What specific experiences have you had in your day-to-day care when caring for patients in palliative situations with COVID-19 and their relatives? - What exactly did your support / care look like? - What was the same / different / new / more / less ... than usual? - What could you (not) achieve? What have you done additionally / otherwise? How did that (not) work? Why exactly (not)? - What worked well? - In which way / through which measures and adjustments to your everyday care (e.g., temporal, spatial, personnel, structural, material, etc.) were you able to maintain your care for seriously ill and dying patients with COVID-19 and their relatives? - What was challenging for you and your care team in the care of seriously ill and dying patients with COVID-19 and their relatives? |
| Specific needs of patients  and relatives in pandemic times | - What additional needs did patients with COVID-19 and their relatives have due to the COVID-19 pandemic? - Which needs / inquiries from COVID-19 patients and their relatives were fewer / more / or less / more / or less / more often than usual due to the pandemic? |
| Solutions in pandemics | - Which solutions from you have proven successful and which have not? - Which solutions have your colleagues tried out and how were these solutions assessed in retrospect? - Would a repetition of this type of work be repeatable / feasible / basically conceivable for you and your care team? |
| Future perspectives for pandemics | - Suppose we were facing another pandemic (with COVID or other diseases) ...   - What would you change / adapt either way?  - How would you specifically prepare for this?  - What would you do / organize in advance, etc.?  - What do you need for that?   - What then do you and your care team need to be able to provide care for critically ill and dying patients affected by a pandemic disease and their relatives? |
| Open themes | - Is there anything else you would like to tell me about your care for patients with COVID-19 and their relatives in the pandemic that I have not yet asked about? |

| 1. **Care of seriously ill and dying patients without COVID-19 and their relatives in pandemic times** | |
| --- | --- |
| **Introduction** | - **Let's now talk about outpatient care for patients in palliative situations without COVID-19 in your facility.** |
| Experiencing the pandemic situation | - How did you experience these patients and relatives being accompanied? - How did you and your care team feel about it? |
| Experiences and challenges in supply in pandemic times | - What specific experiences have you had in your day-to-day care when caring for patients in palliative situations and their relatives? - What exactly did your support / care look like? - What was the same / different / new / more / less ... than usual? - What could you (not) achieve? What have you done additionally / otherwise? How did that (not) work? Why exactly (not)? - What worked well? - In which way / through which measures and adjustments to your everyday care (e.g., temporal, spatial, personnel, structural, material, etc.) were you able to maintain your care for seriously ill and dying patients without COVID-19 and their relatives? - What was challenging for you and your care team in the care of seriously ill and dying patients without COVID-19 and their relatives? |
| Specific needs of patients  and relatives in pandemic times | - What additional needs did patients without COVID-19 and their relatives have due to pandemic? - Which needs / inquiries from patients and their relatives were fewer / more / or less / more / or less / more often than usual due to the pandemic? |
| Solutions in pandemics | - Which solutions from you have proven successful and which have not? - Which solutions have your colleagues tried out and how were these solutions assessed in retrospect? - Would a repetition of this type of work be repeatable / feasible / basically conceivable for you and your care team? |
| Future perspectives for pandemics | - Suppose we were facing another pandemic (with COVID or other diseases) ...   - What would you change / adapt either way?  - How would you specifically prepare for this?  - What would you do / organize in advance, etc.?  - What do you need for that?   - What then do you and your care team need to be able to provide care for critically ill and dying patients affected by a pandemic disease and their relatives? |
| Open themes | - Is there anything else you would like to tell me about your care for patients without COVID-19 and their relatives in the pandemic that I have not yet asked about? |

| 1. **Changes in care in pandemics** | |
| --- | --- |
| **Introduction** | **Now let's talk about the general changes in your daily care routine.** |
| Occupancy | - To what extent have the number of clients or new inquiries changed during the pandemic? - How do you explain that? - What does this mean for your supply processes and structures? - What are the long-term consequences? - What does this mean for the economic situation of your facility? |
| Personnel deployment | - How did you keep your supply staff busy during the pandemic (e.g., short-time work, shift work)? |
| Material storage | - What additional material requirements did you have due to the pandemic? - How did you manage to meet this need? Where and how did you get the material? - What are the additional costs? |
| Rooms | - What measures have you taken regarding home visits to protect everyone from infection? |
| Daily routines and times | - To what extent have you changed your normal daily routines. |
| Cooperations | - Please describe your cooperation with:   - hospitals,  - hospices,  - outpatient specialist palliative services (SAPV),  - outpatient hospice services,  - outpatient care services,  - General practitioners and other general practitioners  To what extent were there changes in cooperation because of the pandemic?   - What should change in the future? |
| Quality of provision | - How do you rate the quality of care for patients and their relatives during the pandemic? |

| **Am Ende des Interviews** | |
| --- | --- |
| Empfehlung von potentiellen Interviewpartner*innen (Schneeball-Prinzip) | Können Sie uns Kolleg*innen aus Einrichtungen der Altenhlfe empfehlen, die schwerkranke und sterbende Patient*innen mit/ohne COVID-19 und ihre Angehörigen in der Pandemiezeit versorgt haben, die wir noch für ein Interview anfragen könnten? |
| Ausblick im Projekt | Ihre Angaben haben uns sehr geholfen und fließen im weiteren Projektverlauf in die Entwicklung einer „Nationale Strategie für Palliativversorgung in Pandemiezeiten“ ein. |
| Rückfragen | Haben Sie noch Fragen an mich? |
| Kontakteinwilligung | Dürfen wir bei Rückfragen nochmals auf Sie zukommen? |
| Dank | Ich danke Ihnen sehr, dass Sie sich die Zeit genommen haben und die Fragen so ausführlich beantwortet haben. Das hilft uns sehr. |
| Gesprächsende | Das Interview ist nun zu Ende. Ich beende jetzt die Audioaufnahme. |
