# Supplementary material for: National strategy for palliative care of severely ill and dying people and their relatives in pandemics (PallPan) in Germany - study protocol of a mixed-methods project
Source: BMC Palliat Care. 2022 Jan 13;21:10. doi: 10.1186/s12904-021-00898-w (PMC8756412; doi:10.1186/s12904-021-00898-w)
Supplement: Supplementary file 5 — Additional file 5: Supplementary file WP2. Interview guide Oncologists. [file 12904_2021_898_MOESM5_ESM.docx]

**PallPan: "National strategy for palliative care in pandemic times"**

**WP 2: General outpatient palliative care (AAPV)**

**Interview guide - oncologists in private practice**

| **Before the interview** | |
| --- | --- |
| Welcome |  |
| Short introduction | My name is ... I am a member of staff in the "PallPan" project at the Institute of General Medicine and palliative Care at Hannover Medical School. I had sent you a short profile about myself. I am very pleased that I can conduct the interview with you today and that you have taken the time for our conversation. |
| Study Objective | I am very pleased to have you share your **experiences and challenges from the COVID-19 pandemic** and your **approaches** to solving them.  We are particularly interested in your **perspective on the care of critically ill and dying patients (with/without COVID-19) and their families** during the COVID-19 pandemic.  Your information will help us to get a realistic picture of this particular situation in Germany. |
| Explanation of the interview process | I will ask you a few open questions below. You can freely tell me what comes to your mind about the respective topic. Since we are at the very beginning of our work, everything is interesting and important for us. |
| Note Reference to Corona | Our questions explicitly refer to this specific Corona situation compared to standard care in ordinary times. |
| Detailed descriptions desired | You are welcome to give detailed answers. In order to be able to process your descriptions for us afterwards, I will have an audio recording device running during the interview. |
| Declaration of consent | *Depending on the situation:*  *You have already received an information and consent form by mail*  *- and returned the signed consent to us.*   - *- However, you have not yet returned the consent. Please do this as a matter of urgency. Do you still have the form or should I send it to you again?* |
| **Anonymity/data protection** | All information you provide will be handled in accordance with the data protection guidelines. This also means that we file all audio recordings and interviews under an anonymous identification number. |
| Questions | Do you have any questions in advance? |
| Start audio recording | I will now start the interview and start the audio recording. |

| 1. **Care for critically ill and dying patients with COVID-19 and their relatives in pandemic times** | |
| --- | --- |
| Screening | - Have you provided outpatient support/care to patients in palliative situations with COVID-19? - If yes, let's talk about these patients first. |
| Experiencing the Pandemic Situation | - How did you experience these patients and their family members? - How did you and your practice team feel about it? |
| Experiences and challenges in pandemic care | - What specific experiences have you had in your daily practice in caring for patients in palliative care situations with COVID-19 and their relatives? - What did your support/care specifically look like? - What was the same/ different/ new/ more/ less... than usual? - What could you (not) provide? What did you do additionally / differently? How did that (not) work? Why exactly (didn't)? - What worked well? - In which way/ by which measures and adaptations of your daily practice (e.g. in terms of time, space, staff, structure, material etc.) were you able to maintain your care of seriously ill and dying patients with COVID-19 and their relatives? - What in the care of seriously ill and dying patients with COVID-19 and their relatives was challenging for you and your practice team? |
| Specific needs of patients  and relatives in times of pandemic | - What additional needs did patients with COVID-19 and their families have due to the COVID-19 pandemic? - Which needs/requests of patients with COVID-19 and their relatives were less/more/ or less frequently/ more often than usual due to the pandemic? |
| Solution approaches in times of pandemic | - Which of your solution approaches have proven successful, and which have not? - Which solutions did your colleagues try out and how did they rate these solutions afterwards? - In how far would this form of work be repeatable/ feasible again/ basically conceivable for you and your practice team? |
| Future perspectives for pandemic times | - Suppose we were facing another pandemic (with COVID-19 or another disease).... - What would you change/adapt one way or the other? - How would you specifically prepare for it? - What would you do/ organize in advance, etc.? - What do you need for it (e.g., stockpiling)? - What do you and your practice team then need to be able to provide care for critically ill and dying patients affected by pandemic disease and their families? |
| Free topics | - Is there anything else you would like to tell me and share with me regarding your care of patients* with COVID-19 and their families during the pandemic period that I have not asked about yet? |

| 1. **Care for seriously ill and dying patients without COVID-19 and their relatives** | |
| --- | --- |
| **Introduction** | **Now let's talk about the outpatient care of patients in palliative situations without COVID-19 in your practice.** |
| Experiencing the pandemic situation | - How did you experience patients and their accompanying family members in pandemic times? - How did you and your practice team feel about it? |
| Experiences and challenges in providing care during pandemics | - What specific experiences have you had in your daily practice in caring for patients in palliative care situations without COVID-19 and their relatives? - What did your care look like specifically? - What was the same/ different/ new/ more/ less... than usual? - What were you (not) able to do? What did you do additionally / differently? How did that (not) work? Why exactly (didn't)? - What worked well? - In which way/ by which measures and adaptations of your daily practice (e.g. in terms of time, space, personnel, structure, material, etc.) were you able to maintain your care for seriously ill and dying patients* without COVID-19 and their relatives? - What in the care of critically ill and dying patients without COVID-19 and their relatives was challenging for you and your practice team in pandemic times? |
| Specific needs of patients | - What additional needs did patients without COVID-19 and their families have as a result of the COVID-19 pandemic? - Which needs/requests of patients* without COVID-19 and their relatives were less/more/ or less frequently/ more often than usual due to the pandemic? |
| Solution approaches in times of pandemic | - Which of your solution approaches have been successful, which have not? - Which solutions did your colleagues try out and how did they rate these solutions afterwards? - Would this form of work be repeatable/ feasible again/ basically conceivable for you and your practice team? |
| Future perspectives for pandemic times | - Suppose we were facing another pandemic (with COVID-19 or another disease)....   - What would you change/adapt one way or the other?  - How would you specifically prepare for it?  - What would you do/ organize, etc. in advance?  - What do you need for this?   - What do you and your practice team need then to be able to provide care for seriously ill and dying patients who are not directly affected by pandemic disease, and their families? |
| Free topics | - Is there anything else you would like to tell me and share with me regarding your care of patients without COVID-19 and their families during the pandemic period that I have not asked about yet? |

| 1. **Changes in everyday practice in times of pandemic.** | |
| --- | --- |
| **Introduction** | **Let's now talk about the general changes in your daily practice routine.** |
| Patient number | - To what extent has the number of patients treated in your private practice changed during the pandemic period? - How do you explain this? - What does this mean for your practice procedures and structures? - What are the consequences in the longer term? - What does this mean for the economic situation of your practice? |
| Staff deployment | - How did you employ your practice staff during the pandemic period (e.g., short-time work, shift scheduling)? |
| Material stockpiling | - What additional material requirements did you have as a result of the pandemic? - How were you able to meet these needs? Where and how did you get the material? - What additional costs did this entail? |
| Spatial arrangement | - How did you divide staff and patient flows in the practice to protect everyone from infection? |
| Consultation formats and times | - To what extent have you changed your usual office hour formats (e.g., phone, video)? - To what extent have you expanded/adjusted your usual office hours (e.g., Wednesdays and Saturdays)? |
| Home visits | - To what extent have you made home visits? - If you made home visits, for which patients and indications, how often, how long, etc.? |
| Cooperations | - Please describe your cooperation with:   - hospitals,  - inpatient hospices,  - outpatient palliative care services,  - outpatient hospice care services,  - outpatient nursing services,  - family doctors and other physicians in private practice   - To what extent have there been changes in cooperation as a result of the pandemic? - What should change in the future? |
| Quality of care | - How do you assess the quality of care for outpatients and their relatives during the pandemic? |

| At the end of the interview | |
| --- | --- |
| Recommendation of potential interview partners (snowball principle) | Can you recommend colleagues from oncology practices who have cared for seriously ill and dying patients with/without COVID-19 and their relatives during the pandemic period, who we could ask for an interview? |
| Outlook in the project | Your information has been very helpful to us and will be used in the further course of the project to develop a "National Strategy for Palliative Care in Pandemic Times". |
| Questions | Do you have any questions for me? |
| Contact consent | May we contact you again if we have any queries? |
| Thanks | Thank you very much for taking the time to answer the questions in such detail. That helps us a lot. |
| End of interview | The interview is now over. I will now end the audio recording. |
