# Supplementary material for: National strategy for palliative care of severely ill and dying people and their relatives in pandemics (PallPan) in Germany - study protocol of a mixed-methods project
Source: BMC Palliat Care. 2022 Jan 13;21:10. doi: 10.1186/s12904-021-00898-w (PMC8756412; doi:10.1186/s12904-021-00898-w)
Supplement: Supplementary file 6 — Additional file 6: Supplementary file WP2. Online Survey Oncologists. [file 12904_2021_898_MOESM6_ESM.docx]

| A Patient contacts | | | | | | | | |
| --- | --- | --- | --- | --- | --- | --- | --- | --- |
|  | | | | | | | | |
| 1. | | | How many patients in your practice do you know have tested positive for SARS-CoV-2? Please think of all your patients, regardless of other illnesses, since the beginning of the pandemic and give the approximate order of magnitude. | | - none - 1-10 - 11-50 - over 50 - no answer | | |  |
|  | | | Filter, at least one patient tested positive was | | | | |  |
| 1.1 | | | In how many of the SARS-CoV-2 positive patients in your practice did the infection take a severe course (e.g., inpatient hospitalization)? | | - Number of patients ___ | | |  |
| 1.2 | | | How many of your practice's SARS-CoV-2 positive patients with a severe course have died? | | - Number of patients ___ | | |  |
|  | | | Filter, for at least one deceased patient | | | | |  |
| 1.2.1 | | | Where did these SARS-CoV-2 positive patients from your practice die?  Number of patients in the respective facilities | | - at home - in care facility - in hospitals - in hospices - no answer | | |  |
| Please think about all critically ill and dying patients in your practice (regardless of COVID-19) when asking the following questions. | | | | | | |  |  |
| 2. | | | What is the total number of critically ill and dying patients you have cared for (completed and ongoing) so far this year? | | - none - 1-10 - 11-50 - 51-100 - over 100 - no answer | |  |  |
| 3. | | | During the initial pandemic peak in spring 2020, did the number of contacts with your critically ill and dying patients change from before the pandemic? | | - yes, more - no, unchanged - yes, less - no answer | |  |  |
| 4. | | | Did the quality of care for your critically ill and dying patients change during the first pandemic peak in the spring? | | - yes, has become better - no, has remained the same - yes, has become worse - no answer | |  |  |
| 5. | | | Did you make home/home visits to critically ill and dying patients during the pandemic? | | - yes - no - no answer | | |  |
|  | | | Filter, for A5 'yes'.... | | | | |  |
| 5.1 | | | Did the number of home/home visits for critically ill and dying patients change during the first pandemic peak in spring compared with before the pandemic? | |  | | |  |
| a) | | | Home visits to seriously ill and dying patients in their own homes | | - yes, more - no, unchanged - yes, less - no answer | | |  |
| b) | | | Visits to seriously ill and dying patients in ursing care facilities | |  |  |  |  |
| c) | | | Visiting patients in hospices | |  |  |  |  |
| 5.2 | | | Notes | | - Free text: ___________ | | |  |
| Availability by phone | | | | | | | |  |
| 6. | During the initial pandemic peak in spring, did you have more telephone contacts instead of face-to-face contacts with your critically ill and dying patients (compared to before the pandemic)? | | | - yes - no - no answer | | | |  |
|  | Filter, for A6 'yes'.... | | | | | | |  |
| 6.1 | Has the quality of care for your critically ill and dying patients changed as a result of telephone contacts instead of face-to-face contacts? | | | - yes, has become better - no, has remained the same - yes, has become worse - no answer | | | |  |
| 6.2 | If change, please justify your answer. | | | - Free text: ___________ | | | |  |
| 7. | During the initial pandemic peak in spring, did you have more telephone contacts instead of face-to-face contacts with family members of critically ill and dying patients (compared with before the pandemic)? | | | - yes - no - no answer | | | |  |
|  | Filter, for A7 'yes'.... | | | | | | |  |
| 7.1 | Has the quality of support for relatives of seriously ill and dying patients changed as a result of telephone contacts instead of personal contacts? | | | - yes, has become better - no, has remained the same - yes, has become worse - no answer | | | |  |
| 7.2 | If change, please justify your answer. | | | - Free text: ___________ | | | |  |
| 8. | To what extent were you personally (or staff in your practice) available to critically ill and dying patients outside of practice hours during the initial pandemic peak in the spring? | | | - always available - reachable at certain times (e.g. on weekends) - not reachable - not answer | | | |  |
| 9. | Notes on accessibility by telephone | | | - Free text: ___________ | | | |  |
| Video consultation | | | | | | | |  |
| 10. | | Are you offering video consultations instead of in-person contacts during the pandemic? | | | | - yes - no - no answer | |  |
| 11. | | Please justify your answer. | | | | - Free text: ___________ | |  |
|  | | Filter, for A10 'yes… | | | | | |  |
| 10.1 | | Has video consultation been used in the care of critically ill and dying patients during the pandemic? | | | |  | |  |
| a) | | at home | | | | - yes, regularly - yes, in individual cases - no, not at all - no answer | |  |
| b) | | in nursing care facilities | | | |  |  |  |
| c) | | In hospices | | | |  |  |  |
| 10.2 | | Has video consultation changed the quality of care for your critically ill and dying patients instead of face-to-face contact? | | | | - yes, has become better - no, has remained the same - yes, has become worse - no answer | |  |
| 10.3 | | Has video consultation been used to accompany relatives of seriously ill and dying patients during the pandemic? | | | | - yes, regularly - yes, in individual cases - no, not at all - no answer | |  |
| 10.4 | | Has the quality of support for relatives of seriously ill and dying patients changed as a result of video consultation instead of personal contact? | | | | - yes, has become better - no, has remained the same - yes, has become worse - no answer | |  |

| B Interfaces | | | | |
| --- | --- | --- | --- | --- |
| *Im* | | | | |
| 1. | During the first pandemic peak in the spring, how did you experience working with... | School grades |  |  |
|  | a) GP colleagues?  b) other specialist colleagues?  c) teams of specialized outpatient palliative care?  d) outpatient care services?  e) outpatient hospice services?  f) physiotherapists, occupational therapists and other therapeutic professions?  g) nursing care facilities?  h) hospices?  i) palliative care units in hospitals?  j) hospitals in general?  k) health care offices? | - 1 very good - 2 good - 3 satisfactory - 4 sufficient - 5 inadequate - 6 insufficient - No answer |  |  |
| 2. | Notes | - Free text: ___________ |  |  |
| 3a. | Were there any of the following problems with admitting critically ill and dying patients to nursing facilities during the initial pandemic peak in spring? | - Admission only possible after quarantine - Admission only possible after submission of a negative test result - Admission only possible after long waiting time - no, there were no problems - no answer |  |  |
| 3b. | Were there any of the following problems with hospice admissions of critically ill and dying patients during the initial pandemic peak in spring? | - Admission only possible after quarantine - Admission only possible after submission of a negative test result - Admission only possible after long waiting time - no, there were no problems - no answer |  |  |
| 3c. | Were there the following problems in admitting critically ill and dying patients to palliative care units in hospitals during the first pandemic peak in spring? | - Admission only possible after quarantine - Admission only possible after submission of a negative test result - Admission only possible after long waiting time - no, there were no problems - no answer |  |  |
| 3d. | Were there the following problems in admitting critically ill and dying patients to specialized outpatient palliative care during the first pandemic peak in spring? | - Admission only possible after quarantine - Admission only possible after submission of a negative test result - Admission only possible after long waiting time - no, there were no problems - no answer |  |  |
| 4. | Notes | - Free text: ___________ |  |  |
| 5. | Were the hygiene concepts of nursing care facilities in your catchment area uniform? | - yes - no - no answer |  |  |
|  | Filter, bei B5 ´nein´ | |  |  |
| 5.1 | What problems has this created for your patient care? | - Free text: ___________ |  |  |
| 6. | Please name aspects of hygiene concepts in nursing care facilities that are particularly relevant from your point of view? | - Free text: ___________ |  |  |
| 7. | Were relatives able to visit critically ill and dying patients in ... during the first pandemic peak phase in the spring? |  | |  |
| a) | Nursings care facilities | - yes - partly - no - no answer | |  |
| b) | Hospices |  |  |  |
| c) | Palliative care units in hospitals |  |  |  |
|  | Notes | - Free text: ___________ | |  |
| **Impact of contact restrictions and visitation bans.** | | | |  |
| 8. | As a result of the contact restrictions and visitation bans, were there any changes in ...  a) at home  b) in nursing care facilities  b) in inpatient hospices  c) in palliative care units in hospitals |  | |  |
| 8.1 | the physical health of seriously ill and dying patients? | - yes, improvement   no, no change   - yes, worsening - no answer | |  |
| 8.2 | the mental health of seriously ill and dying patients? |  |  |  |

| C Psychosocial aspects | | | |
| --- | --- | --- | --- |
| Please rate the extent to which the following statements are true. | | | |
| Please rate the extent to which the following statements are true. | | | |
| 1. | | Seriously ill and dying patients express fears during the pandemic, ... | - strongly apply - rather apply - rather does not apply - does not apply - no answer |
| 1.1 | | to contract corona (COVID-19). |  |
| 1.2 | | becoming lonely at home. |  |
| 1.3 | | becoming lonely in nursing care facilities. |  |
| 1.4 | | becoming lonely in hospitals. |  |
| 1.5 | | to be given lower priority care in the event of hospitalization. |  |
| 1.6 | | to be overtreated in the event of hospitalization. |  |
| **Please rate the extent to which the following statements are true.** | | | |
| 2.1 | Relatives receive less information about seriously ill and dying patients due to contact restrictions and visitation bans. | | - strongly apply - rather apply - rather does not apply - does not apply - no answer |
| 2.2 | Relatives find it particularly stressful not to be able to support seriously ill and dying patients with their physical presence and closeness. | |  |
| 2.3 | Saying goodbye to relatives of seriously ill and dying patients is possible only to a very limited extent due to contact restrictions and visitation bans. | |  |
| 2.4 | Saying goodbye to relatives of seriously ill and dying patients is not possible due to contact restrictions and visitation bans. | |  |
| 2.5 | Notes | | Free text: ___________ |

| D Living wills | | |
| --- | --- | --- |
| *Im* | | |
|  | Please rate the extent to which the following statements apply to your practice. |  |
| 1. | During the pandemic, there is an increased demand from critically ill and dying patients for the creation of advance directives. | - strongly apply - rather apply - rather does not apply - does not apply - no answer |
| 2. | During the pandemic, there is an increased demand from critically ill and dying patients to review or modify existing advance directives. |  |
| 3. | Comments (e.g., special concerns and questions from patients, COVID-19 related issues). | - Free text: ________ |

| E Information flow during the pandemic | | |
| --- | --- | --- |
| 1. | What sources did you use to obtain information on the care of critically ill and dying patients during the first pandemic peak phase in the spring?  a) Robert Koch Institute (RKI)  b) Public health department  c) Association of Statutory Health Insurance Physicians (KV)  d) General practitioners` association  e) German Society for General and Family Medicine (DEGAM)  f) German Society for Palliative Medicine (DGP)  g) Others: ___________ | - strongly apply - rather apply - rather does not apply - does not apply - no answer |
| 2. | What information were you missing when caring for critically ill and dying patients during the pandemic? | - Free text: ________ |

| F Solution approaches | | | |
| --- | --- | --- | --- |
| 1. | What is particularly important for your care of critically ill and dying patients during pandemic peaks?  Please name the three most important aspects for you. | - Free text: ________ - Free text: ________ - Free text: ________ |  |
| 2. | What should not be restricted / omitted during periods of lockdown in the care of critically ill and dying patients? | - Free text: ________ |  |
|  | Which of the following measures should be implemented during pandemic highs? |  |  |
| 3. | Care for patients in nursing care facilities should be provided by defined home health aides. | - strongly apply - rather apply - rather does not apply - does not apply - no answer |  |
| 4. | Family physicians should be involved in local crisis teams. |  |  |
| 5. | Palliative care professionals should be involved in local crisis teams. |  |  |
| 6. | Other solution approaches | - Free text: ________ |  |

| G Questions about yourself and your practice | | | | | | | | | | | | | | | | | | | | | |
| --- | --- | --- | --- | --- | --- | --- | --- | --- | --- | --- | --- | --- | --- | --- | --- | --- | --- | --- | --- | --- | --- |
| 1. | | What gender are you? | | | | | | | | | | | | | | | | | | | |
|  | | □ | | male | | | | □ | female | | | | | | | □ | divers | | | | |
| 2. | | How old are you?______ (years) | | | | | | | | | | | | | | | | | | | |
| 3. | In which state is your practice located? Praxis? | | | | | | | | | | | | | | | | | | | | |
|  | ________________________________ | | | | | | | | | | | | | | | | | | | | |
| 4. | | What is the population of the town where your practice is located? | | | | | | | | | | | | | | | | | | | |
|  | | □ | | | less than 5.000 inhabitants | | | | | |  | | □ | | between 20.000 and 100.000 inhabitants | | | | | | |
|  | | □ | | | between 5,000 and 20,000 inhabitants | | | | | |  | | □ | | more than 100,000 inhabitants | | | | | | |
| 5. | | What type of practice do you work in? | | | | | | | | | | | | | | | | | | | |
|  | | □ | | Individual practice | | | □ | Joint practice or group practice | | | | | | | | | | □ | | Medical care center | |
| 6. | | Are you a specialist for ...? | | | | | | | | | | | | | | | | | | | |
|  | | □ | | | General medicine | | | | | |  | | □ | | Internal medicine  (general practitioner) | | | | | | |
|  | | □ | | | Other specialty | | | | | |  | | □ | | Physician in further training | | | | | | |
|  | | If other specialty, please indicate which. | | | | | | | | | | | | | _________________________ | | | | | | |
| 7. | | | Do you have additional training in palliative care? | | | | | | | | | | | | | | | | | | |
|  | | | □ | | | Yes | | | |  | | □ | | No | | | | | | | |
| Filter, for G7 'yes'... | | | | | | | | | | | | | | | | | | | | | |
| 8. | | | Do you work as a specialized palliative care doctor yourself? | | | | | | | | | | | | | | | | | | |
|  | | | □ | | | Yes | | | |  | | □ | | No | | | | | | | |
| **Personal burdens** | | | | | | | | | | | | | | | | | | | | |  |
| 9. | The final question refers to your stresses. What did you personally find particularly stressful during the first pandemic peak in spring? | | | | | | | | | | | | | | | | | | | |  |
|  | 1. your own increased workload 2. increased sick leave in the team 3. more conflicts in the team 4. implementation of stricter hygiene regulations in the practice 5. financial situation of the practice 6. less contact with colleagues 7. compliance with distance rules to patients 8. concerns about own infection 9. concern about infection of own family 10. Other:______________ | | | | | | | | | | | | | | | | | | - strongly apply - rather apply - rather does not apply - does not apply - no answer | |  |
