# Supplementary material for: National strategy for palliative care of severely ill and dying people and their relatives in pandemics (PallPan) in Germany - study protocol of a mixed-methods project
Source: BMC Palliat Care. 2022 Jan 13;21:10. doi: 10.1186/s12904-021-00898-w (PMC8756412; doi:10.1186/s12904-021-00898-w)
Supplement: Supplementary file 7 — Additional file 7: Supplementary file WP3. Interview Guide SPHC. [file 12904_2021_898_MOESM7_ESM.docx]

**Introduction**

Thank you for investing the time to participate in an interview on ‘Specialized Palliative Home Care in Times of the Covid-19-Pandemic’.

- Privacy:

As you already know, I want to record the interview audio digitally to analyse it later. The recording will not be published. If you agree with the recording please say YES. Now, after starting the recording, I have to ask you again: If you agree with the recording, please say YES.

- Please start with telling me a little bit about yourself and your work.

**Effects of COVID-19-Pandemic to Specialized Palliative Home Care**

Thank you for the introduction. We want to speak about palliative care in pandemic times. Please tell me, how the pandemic affected the work of specialist palliative home care teams from the perspective of a state association? Which challenges where brought to your attention, and how did you deal with them?

Deepening Questions:

1. Perspectives: Patients (suffering from COVID-19, infected with SARS-COV-2 or not infected), relatives, team and cooperation (especially: volunteers)
2. Pandemic Phases: Were there differences between pandemic phases (first wave in March and April vs. low incidence during summer)?

**Umgang mit den Veränderungen**

How were challenges met?

How did the state association support the teams in your region?

What worked well and what didn’t?

What challenges are specialist palliative homecare teams currently facing?

**Strategies and solution**

For the ongoing second wave…

…what strategies and solutions would you chose again to deal with a pandemic? Where do you need more support?

Or: What do you wish for specialised palliative home care in times of pandemics?
