# Supplementary material for: National strategy for palliative care of severely ill and dying people and their relatives in pandemics (PallPan) in Germany - study protocol of a mixed-methods project
Source: BMC Palliat Care. 2022 Jan 13;21:10. doi: 10.1186/s12904-021-00898-w (PMC8756412; doi:10.1186/s12904-021-00898-w)
Supplement: Supplementary file 8 — Additional file 8: Supplementary file WP3. Online Survey SPHC. [file 12904_2021_898_MOESM8_ESM.docx]

Pallpan: "National Strategy for Palliative Care in Pandemic Times"

WP3: Specialist Palliative Home Care (SAPV) Online survey

# Introductory text:

Dear Colleague,

SAPV (**S**pezialisierte **A**mbulante **P**alliativ **V**ersorgung, in English: specialized outpatient palliative care) teams have faced and continue to face the challenge of adapting their mode of operation to the unique challenges of the COVID-19 pandemic.

As part of the project "Palliative Care in times of Pandemics (PallPan)", we want to develop a National Strategy for Palliative Care in Pandemic Times. We would therefore be pleased if you would share with us your experiences during the COVID-19 pandemic, the challenges associated with it, and your approaches in order to deal with them. To map the course of the pandemic, we ask you for some questions to distinguish between the initial peak phase and the later phases of the pandemic.

Our research project aims to contribute to the improvement of outpatient care for critically ill and dying patients during pandemic periods.

The present survey is conducted by the University Hospitals of Jena and Göttingen in collaboration with the joint project Research Network Palliative Medicine.

A nationwide full survey of all SAPV teams is planned for this purpose. Please note that it is important that only one person per SAPV team (coordinator, medical or nursing director) participates in the online survey.

The survey will take about 30 minutes to complete. We are interested in your views and experiences. Therefore, there are no "right" or "wrong" answers.

If you have any questions about this survey or the questions themselves, please feel free to contact the research team.

We greatly value your input. Thank you for your participation

Yours sincerely

PD Dr. Ulrich Wedding, Cordula Gebel, Lars Kloppenburg

O I hereby confirm that I have read and understood the information on data privacy and protection

# Information On data privacy and protection

Responsible head of the study:

Mr. PD Dr. Ulrich Wedding, Clinic of Internal Medicine II - Division of Palliative Medicine, Universitätsklinikum Jena, Am Klinikum 1, 07747 Jena, Phone.: +49-3641-9327500, e-Mail: ulrich.wedding@med.uni-jena.de

Contact for questions:

Mrs. Cordula Gebel, Phone: +49-3641-932751, e-Mail: cordula.gebel@med.uni-jena.de

Data Privacy Officer:

Center for Health and Safety Management, Data Protection Officer of the University Hospital Jena, Bachstraße 18, 07743 Jena, E-Mail: [Datenschutzbeauftragter@med.uni-jena.de](mailto:Datenschutzbeauftragter@med.uni-jena.de)

The study is part of the joint project Forschungsnetz Palliativmedizin "National Strategy for Palliative Care in Times of Pandemic (PallPan)." This project is funded as part of the University Medicine Network (Netzwerk Universitätsmedizin (NUM)) by the Federal Ministry of Education and Research in Germany (Bundesministerium für Bildung und Forschung (BMBF)).

The Ethics Committee of the University Hospital of Jena gave its approval to conduct the study (No. 2020-1848-Bef.).

What happens with your data?

Your data generated in the course of the survey will be sent in encrypted form and processed and evaluated exclusively for scientific purposes. The data will be stored in the data center of the University Hospital Jena for an estimated 2-3 years until the scientific evaluation is completed. The privacy policy of the University Hospital Jena also applies to indirectly collected and stored data, which is generated in the course of providing the web application. This information can also be found on the Internet at:

https://www.uniklinikum-jena.de/Datenschutz.html

Your participation in our study is voluntary. You will not suffer any disadvantages if you do not participate. Third parties will not be given access to personal data. You have the right to request information about whether and which personal data are processed by us. Under certain circumstances, you have the right to request restriction of the processing of your data, in particular if the accuracy of the data is disputed by you, the processing of the data is unlawful, the data is no longer required for processing, or an objection to the data processing has been filed. Under certain conditions, you have the right to request the immediate deletion of your personal data.

If you believe that we have not complied with data protection regulations when processing your data, you can lodge a complaint with the responsible supervisory authority, which will investigate your complaint.

Your declarations regarding the assertion of your rights must always be addressed in writing to the person responsible.

| Question | Input content |
| --- | --- |
| Section 1 |  |
| Have you cared for patients with SARS-CoV-2 infections? | - Yes - No - Don’t know |
| If yes: How many? | - ____Number of patients |
| Where have you cared for COVID-19 patients? | - At home/ - Nursing home/ - Hospice/ - Institution of integration assistance - Refugee Facility - Homeless Facility - Other: |
| Have you cared for patients with SARS-CoV-2 infections? | - Yes - No - not assessable |
| ►If yes:  Was the SARS-CoV-2 infection the reason for palliative care? | - Yes - No - not assessable |
| How have your patient numbers changed during the COVID 19 pandemic?  Compared to the pre-pandemic period, the number of patients... | |
| a)… during the 1st peak phase of the pandemic (March/April) was… | - significantly smaller - smaller - approximately unchanged - greater - significantly greater   - not assessable |
| b)… after the end of the lockdown (from May on) was… | - significantly smaller - smaller - approximately unchanged - greater - significantly greater   - not assessable |
| c)… today is… | - significantly smaller - smaller - approximately unchanged - greater - significantly greater   - not assessable |
| ►If the number of patients has changed:  What reasons do you see for this? | - Earlier discharge of patients from hospitals - Patients do not want to go to hospital - Hospice does not accept patients (or only after quarantine) - Nursing home does not accept patients (or only after quarantine) - Fewer allocations from nursing homes - Increased symptom burden due to shortfalls in other therapies - Lack of accessibility of other care providers (including primary care or specialty care) - Internal organizational problems (personnel, protective clothing, etc...) - Other: _______ |
| Section 2 |  |
| Was there a change in patient populations (e.g., type of disease, age, indications) in light of the pandemic? | - Yes - No - not assessable |
| ► If yes: What changes have there been? | free text |
| ► If yes: What reasons do you see for this? | - Earlier discharge of patients from hospitals - More care in nursing homes - Fewer care in nursing homes - Lack of accessibility of other care providers (including primary care or specialty care) - Hospice does not accept patients (or only after quarantine) - Nursing home does not accept patients (or only after quarantine) - Prescription of SAPV to avoid otherwise indicated hospitalization, e.g., for severe pneumonia - Other:_________ |
| Have living wills or precautionary powers of attorney been reviewed and, if necessary, updated in light of the pandemic? | - Yes - No - not assessable - (If yes) Here you can explain your statement:______________ |
| Have there been changes in therapy goal setting in light of the pandemic? | - Yes - No - not assessable - (If yes) Here you can explain your statement:______________ |
| Have there been any changes in how the deceased are handled in light of the pandemic? | - Yes - No - not assessable - (If yes) Here you can explain your statement:______________ |
| To what extent do the following statements apply to your SAPV team compared to before the pandemic?  During the COVID-19 pandemic, the... | |
| - number of telephone consultations with utilities | - has fallen significantly - has fallen - stayed approximately unchanged - has increased - has increased significantly - not assessable |
| - number of telephone contacts with patients/relatives | - has fallen significantly - has fallen - stayed approximately unchanged - has increased - has increased significantly - not assessable |
| - number of home visits | - has fallen significantly - has fallen - stayed approximately unchanged - has increased - has increased significantly - not assessable |
| - waiting time of patients/relatives for a home visit | - has fallen significantly - has fallen - stayed approximately unchanged - has increased - has increased significantly - not assessable |
| - number of crisis situations with patients | - has fallen significantly - has fallen - stayed approximately unchanged - has increased - has increased significantly - not assessable |
| - the amount of patient care in nursing homes | - has fallen significantly - has fallen - stayed approximately unchanged - has increased - has increased significantly - not assessable |
| - the amount of patient care in inpatient hospices | - has fallen significantly - has fallen - stayed approximately unchanged - has increased - has increased significantly - not assessable |
| - the frequency of the involvement of relatives | - has fallen significantly - has fallen - stayed approximately unchanged - has increased - has increased significantly - not assessable |
| - the number of referrals by general practitioners | - has fallen significantly - has fallen - stayed approximately unchanged - has increased - has increased significantly - not assessable |
| - the number of referrals by hospitals | - has fallen significantly - has fallen - stayed approximately unchanged - has increased - has increased significantly - not assessable |

| Question | Input content |
| --- | --- |
| Section 3 |  |
| To what extent did you have problems providing patient care during the COVID 19 pandemic? | - no problems - few problems - mediocre problems - severe problems - very severe problems - not assessable |
| To what extent do the following statements apply to your team during the 1st peak phase of the pandemic (March/April)? | |
| - Our SAPV team was able to treat the patients' symptoms as usual. | - does not apply - somewhat applies - partially applies - rather applies - fully applies - not assessable |
| - Our SAPV team was able to offer patients and relatives sufficient emotional support. | - does not apply - somewhat applies - partially applies - rather applies - fully applies - not assessable |
| - Our team was able to accompany patients and relatives in decision-making processes as usual | - does not apply - somewhat applies - partially applies - rather applies - fully applies - not assessable |
| - Despite the uncertainties in light of the pandemic, our team was able to convey a sense of security and safety to patients and their families. | - does not apply - somewhat applies - partially applies - rather applies - fully applies - not assessable |
| - We were able to provide palliative care for patients with SARS-CoV-2 infections. | - does not apply - somewhat applies - partially applies - rather applies - fully applies - not assessable |

| Question | Input content |
| --- | --- |
| Is there anything else you would like to add, explain or comment concerning patient care? | Free text |

# EXPERIENCEs AT THE ORGANIZATIONAL LEVEL TO ENSURE SAPV

Please share with us your experiences on the organizational level. The first questions to follow are some general questions about your SAPV team.

| Question | Input content | |  |
| --- | --- | --- | --- |
| Section 1 |  | |  |
| In which county(ies) does your SAPV team primarily operate?  Location of operation/County of operation:*  *This information is used exclusively to categorize the COVID-19 risk situation according to the RKI. |  | |  |
| KV-district | - Baden-Wuerttemberg (KVBW) - Bavaria (KVB) - Berlin (KV Berlin) - Brandenburg (KVBB) - Bremen (KVHB) - Hamburg (KVH) - Hessen (KV Hessen) - Mecklenburg-Western Pomerania (KVMV) - Lower Saxony (KVN) - North Rhine (KVNO) - Rhineland-Palatinate (KV RLP) - Saarland (KV Saarland) - Saxony (KVS) - Saxony-Anhalt (KVSA) - Schleswig-Holstein (KVSH) - Thuringia (KV Thüringen) - Westphalia-Lippe (KVWL) | |  |
| How many cases of SAPV care did you provide in 2019? | - Less than 250 - 250-350 - 351-450 - 451-550 - More than 550 | | |
| Were colleagues in your SAPV team infected with SARS-CoV-2? | | - Yes - No - Don’t know | |
| Were colleagues from your SAPV team in quarantine? | | - Yes - No - Don’t know | |
| Were there any staff absences on your team due to the pandemic-related multiple responsibilities, e.g., homeschooling, caring for dependents? | | - Yes - No - Don’t know | |

| Question | Input content |
| --- | --- |
| To what extent has your day-to-day work changed because of the COVID 19 pandemic? | - no change at all - little change - medium changes - large changes - very large changes - not assessable |
| How much have the following COVID-19 prevention requirements affected your work? | |
| - Hygiene regulations | - no impairment - little impairment - medium impairment - large impairment - very large impairment - there were no changes in this scope |
| - Avoidance of staff contact with patients and relatives | - no impairment - little impairment - medium impairment - large impairment - very large impairment - there were no changes in this scope |
| - Compliance with distance rules for patients and relatives | - no impairment - little impairment - medium impairment - large impairment - very large impairment - there were no changes in this scope |
| - Avoidance of contacts within the team | - no impairment - little impairment - medium impairment - large impairment - very large impairment - there were no changes in this scope |
| - Avoidance of contacts and contact minimization with network partners | - no impairment - little impairment - medium impairment - large impairment - very large impairment - there were no changes in this scope |
| - Restrictions/prohibitions on visits by relatives | - no impairment - little impairment - medium impairment - large impairment - very large impairment - there were no changes in this scope |
| - Restrictions/prohibitions on visits by hospice services/volunteers | - no impairment - little impairment - medium impairment - large impairment - very large impairment - there were no changes in this scope |
| Section 2 |  |
| To what extent do you have/had difficulties with | |
| - the procurement of protective equipment? | **In the 1st peak of the pandemic (March/April)**   - non - little - mediocre - large - very large - not assessable   **currently**   - non - little - mediocre - large - very large - not assessable   Here you can explain your statement: ________free text |
| - the financing of protective equipment? | **In the 1st peak of the pandemic (March/April)**   - non - little - mediocre - large - very large - not assessable   **currently**   - non - little - mediocre - large - very large - not assessable   Here you can explain your statement: ________free text |
| - the testing possibilities/testing capacities for your colleagues? | **In the 1st peak of the pandemic (March/April)**   - non - little - mediocre - large - very large - not assessable   **currently**   - non - little - mediocre - large - very large - not assessable   Here you can explain your statement: ________free text |
| - the testing possibilities/testing capacities for your patients? | **In the 1st peak of the pandemic (March/April)**   - non - little - mediocre - large - very large - not assessable   **currently**   - non - little - mediocre - large - very large - not assessable   Here you can explain your statement: ________free text |
| - the supply of medicines? | **In the 1st peak of the pandemic (March/April)**   - non - little - mediocre - large - very large - not assessable   **currently**   - non - little - mediocre - large - very large - not assessable   Here you can explain your statement: ________free text |
| Where could you acquire protective equipment from? | Sponsoring organization of the SAPV team  Public health office  KV  Hospital (if not sponsoring organization)  Pharmacy   - District administration/county council/city - Other: ___________ free text |
| How would you rate your technological equipment (computers, tablets, phones...) to meet the challenge of the COVID 19 pandemic? | - very bad - bad - medium - good - very good |
| What technique did you increase your use of as a result of the pandemic? | - Phone calls/teleconferences - Video-based communication with laptop, tablet or smartphone - E-Mails - Messenger services e.g. WhatsApp, Signal, Telegram - Information sheets for patients/relatives - Mobile documentation - Other:______________ |
| Despite the pandemic and the resulting changes in communication channels, communication has been functioning smoothly... | |
| - Within the team | - does not apply - somewhat applies - partially applies - rather applies - fully applies - not assessable |
| - between the patients/relatives and us | - does not apply - somewhat applies - partially applies - rather applies - fully applies - not assessable |
| - with care partners (e.g. nursing service, family doctor) | - does not apply - somewhat applies - partially applies - rather applies - fully applies - not assessable |
| Section 3 |  |
| To what extent do the following statements apply to your team during the first height of the pandemic (March/April)? | |
| - Necessary structures within the team could be established quickly. | - does not apply - somewhat applies - partially applies - rather applies - fully applies - not assessable |
| - Due to the different regulations (e.g. from sponsoring organizations, counties, cooperation partners), we were able to carry out our core activities with more difficulty. | - does not apply - somewhat applies - partially applies - rather applies - fully applies - not assessable |
| - There was a lot of uncertainty in the team due to continuously changing regulations. | - does not apply - somewhat applies - partially applies - rather applies - fully applies - not assessable |
| - The bundling and forwarding of official information within the team went smoothly. | - does not apply - somewhat applies - partially applies - rather applies - fully applies - not assessable |

| Question | Input content |
| --- | --- |
| Have you adjusted your documentation by relevant disease symptoms, e.g., specific COVID-19 symptoms or body temperature? | - Yes - No - Not assessable |
| Were there any problems with the approval of SAPV services by the MDK in light of the pandemic? | - Yes - No - Not assessable - (If yes) Here you can explain your statement: ______ |
| Do you expect any financial losses as a result of the COVID-19 pandemic? | - No assessment possible at present - No - Yes, minor losses - Yes, significant losses |
| (If yes) How did the losses occur? | - Higher costs - Lower income - both |

| Question | Input content |
| --- | --- |
| Is there anything else you would like to add, explain or comment on the topic of organization and communication? |  |

Staff/Team

Please share with us your experience within the team.

| Question | Input content |
| --- | --- |
| To what extent do the following statements apply to your team during the 1st peak phase of the pandemic (March/April)? | |
| - Door-to-door conversations were missing for the exchange. | - does not apply - somewhat applies - partially applies - rather applies - fully applies |
| - The planning of the tours was more elaborate than usual. | - does not apply - somewhat applies - partially applies - rather applies - fully applies |
| - The spirit at work was good. | - does not apply - somewhat applies - partially applies - rather applies - fully applies |
| - There were more conflicts between colleagues during work. | - does not apply - somewhat applies - partially applies - rather applies - fully applies |
| - There was a higher workload. | - does not apply - somewhat applies - partially applies - rather applies - fully applies |
| - There were enough colleagues to cover the supply needs. | - does not apply - somewhat applies - partially applies - rather applies - fully applies |
| - More overtime has accrued. | - does not apply - somewhat applies - partially applies - rather applies - fully applies |
| - There have been more sick calls. | - does not apply - somewhat applies - partially applies - rather applies - fully applies |
| - We were able to work as a multi-professional team as usual. | - does not apply - somewhat applies - partially applies - rather applies - fully applies |
| - We were able to include, for example, physiotherapy, pastoral care, psychology, social work in patient care as usual. | - does not apply - somewhat applies - partially applies - rather applies - fully applies |

| Question | Input content |
| --- | --- |
| Is there anything else you would like to add, explain or comment on the topic of staff and the SAPV team? | Free text |

# Cooperation und Network

Please share with us your experiences with cooperation partners and networks.

| Question | Input content |
| --- | --- |
| Compared to the pre-pandemic period, cooperation with... | |
| - Nursing homes | **In the 1st peak of the pandemic (March/April)**   - was significantly harder - was harder - stayed unchanged - was better - was significantly better   **currently**   - was significantly harder - was harder - stayed unchanged - was better - was significantly better |
| - Family doctors | **In the 1st peak of the pandemic (March/April)**   - was significantly harder - was harder - stayed unchanged - was better - was significantly better   **currently**   - was significantly harder - was harder - stayed unchanged - was better   was significantly better |
| - established medical specialists | **In the 1st peak of the pandemic (March/April)**   - was significantly harder - was harder - stayed unchanged - was better - was significantly better   **currently**   - was significantly harder - was harder - stayed unchanged - was better   was significantly better |
| - outpatient care services | **In the 1st peak of the pandemic (March/April)**   - was significantly harder - was harder - stayed unchanged - was better - was significantly better   **currently**   - was significantly harder - was harder - stayed unchanged - was better   was significantly better |
| - inpatient hospices | **In the 1st peak of the pandemic (March/April)**   - was significantly harder - was harder - stayed unchanged - was better - was significantly better   **currently**   - was significantly harder - was harder - stayed unchanged - was better   was significantly better |
| - outpatient hospice services | **In the 1st peak of the pandemic (March/April)**   - was significantly harder - was harder - stayed unchanged - was better - was significantly better   **currently**   - was significantly harder - was harder - stayed unchanged - was better   was significantly better |
| - Palliative Care Unit | **In the 1st peak of the pandemic (March/April)**   - was significantly harder - was harder - stayed unchanged - was better - was significantly better   **currently**   - was significantly harder - was harder - stayed unchanged - was better   was significantly better |
| - Hospital | **In the 1st peak of the pandemic (March/April)**   - was significantly harder - was harder - stayed unchanged - was better - was significantly better   **currently**   - was significantly harder - was harder - stayed unchanged - was better   was significantly better |
| Were contact persons in the following organizational structures helpful to you? | |
| - Public Health Department | - Yes - No - No contact person and not needed - No contact person even though it would have been helpful - not assessable |
| - local pandemic crisis unit | - Yes - No - No contact person and not needed - No contact person even though it would have been helpful - not assessable |
| - Health insurance companies | - Yes - No - No contact person and not needed - No contact person even though it would have been helpful - not assessable |
| - MDK | - Yes - No - No contact person and not needed - No contact person even though it would have been helpful - not assessable |
| - SAPV state associations | - Yes - No - No contact person and not needed - No contact person even though it would have been helpful - not assessable |
| - local hospital | - Yes - No - No contact person and not needed - No contact person even though it would have been helpful - not assessable |
| - Other: | Free text |
|  |  |
| What sources did you use to gather information? | - RKI - Professional associations - SAPV State Association - German Society for Palliative Medicine - German Hospice and Palliative Association - Information of the city - Information of the county - Information of the state - Journals - General media - Social media - Others: |

# SOLUTION APPROACHES

| Question | Input content |
| --- | --- |
| We have compiled some measures to deal with the COVID-19 pandemic, for each measure please mark if  a) you have implemented it, b) you find it useful but have not implemented it, c) you do not find it useful, or whether d) this measure does not seem feasible from your perspective. | |
| Preparation of a pandemic plan | - We have implemented this. - We find this useful but have not implemented it. - We do not find this useful. - This seems not feasible to us. - no specification |
| Development of a written standard procedure for a suspected case among patients, relatives, employees | - We have implemented this. - We find this useful but have not implemented it. - We do not find this useful. - This seems not feasible to us. - no specification |
| Development of a written standard procedure for an infection case among patients, relatives, employees | - We have implemented this. - We find this useful but have not implemented it. - We do not find this useful. - This seems not feasible to us. - no specification |
| Create/revise hygiene plan | - We have implemented this. - We find this useful but have not implemented it. - We do not find this useful. - This seems not feasible to us. - no specification |
| Time and space-based patient management, e.g. via a dedicated COVID-19 SAPV team | - We have implemented this. - We find this useful but have not implemented it. - We do not find this useful. - This seems not feasible to us. - no specification |
| Drive to home visits by private car | - We have implemented this. - We find this useful but have not implemented it. - We do not find this useful. - This seems not feasible to us. - no specification |
| Contact prior to home visits to check for COVID-19 symptoms | - We have implemented this. - We find this useful but have not implemented it. - We do not find this useful. - This seems not feasible to us. - no specification |
| Personal contact/home visits only after strict indication evaluation | - We have implemented this. - We find this useful but have not implemented it. - We do not find this useful. - This seems not feasible to us. - no specification |
| Information sheets for patients and relatives on conditions of SAPV care | - We have implemented this. - We find this useful but have not implemented it. - We do not find this useful. - This seems not feasible to us. - no specification |
| Establishment of home office for SAPV employees | - We have implemented this. - We find this useful but have not implemented it. - We do not find this useful. - This seems not feasible to us. - no specification |
| Establishment of other measures to adapt the organization of work, e.g. changes in the shift system | - We have implemented this. - We find this useful but have not implemented it. - We do not find this useful. - This seems not feasible to us. - no specification |
| Restriction of contact with volunteers for infection control | - We have implemented this. - We find this useful but have not implemented it. - We do not find this useful. - This seems not feasible to us. - no specification |
| Measures to improve the bond among employees, e.g. through common rituals | - We have implemented this. - We find this useful but have not implemented it. - We do not find this useful. - This seems not feasible to us. - no specification |
| Measures to support health workers in dealing with stress/anxiety and uncertainty | - We have implemented this. - We find this useful but have not implemented it. - We do not find this useful. - This seems not feasible to us. - no specification |
| Supervision on the topic of "Dealing with the Pandemic" | - We have implemented this. - We find this useful but have not implemented it. - We do not find this useful. - This seems not feasible to us. - no specification |
| Stockpiling or management concept for rapid procurement of medicines and protective clothing | - We have implemented this. - We find this useful but have not implemented it. - We do not find this useful. - This seems not feasible to us. - no specification |
| Training regarding infection control and hygiene measures | - We have implemented this. - We find this useful but have not implemented it. - We do not find this useful. - This seems not feasible to us. - no specification |
| Training regarding clinical picture and course of the pandemic disease | - We have implemented this. - We find this useful but have not implemented it. - We do not find this useful. - This seems not feasible to us. - no specification |
| Sharing standards/advice and protocols with other SAPV teams | - We have implemented this. - We find this useful but have not implemented it. - We do not find this useful. - This seems not feasible to us. - no specification |
| Local networking, e.g. with nursing homes, various sponsoring organizations | - We have implemented this. - We find this useful but have not implemented it. - We do not find this useful. - This seems not feasible to us. - no specification |
| Clear internal responsibilities for information search, bundling and dissemination | - We have implemented this. - We find this useful but have not implemented it. - We do not find this useful. - This seems not feasible to us. - no specification |
| Legal contact persons, e.g. to clarify whether home visits can be carried out | - We have implemented this. - We find this useful but have not implemented it. - We do not find this useful. - This seems not feasible to us. - no specification |

| Question | Input content |
| --- | --- |
| Have you developed your own solutions to problems that you would recommend? |  |

# COURSE OF THE PANDEMIC

Please share with us how you are experiencing the course of the pandemic.

| Question | Input content |
| --- | --- |
| To what extent do the following statements currently apply to your team?  "As a team, we are well prepared for another intensification of the situation/a so-called "second wave"." | - does not apply - somewhat applies - partially applies - rather applies - fully applies |

| How do you assess **uncertainty in the team regarding SARS-CoV-2** before and at each stage of the pandemic? | | | | |
| --- | --- | --- | --- | --- |
|  | Before the pandemic  (January/February) | 1^st^ peak/”1^st^ wave”  (March/April) | Intermediate stage  (May until September) | 2^nd^ peak/”2^nd^ wave”  (from October on) |
| Very large |  |  |  |  |
| Large |  |  |  |  |
| Medium |  |  |  |  |
| Small |  |  |  |  |
| Very small |  |  |  |  |
| How do you estimate the **extent of organizational activities** before and at each stage of the pandemic? | | | | |
|  | Before the pandemic  (January/February) | 1^st^ peak/”1^st^ wave”  (March/April) | Intermediate stage  (May until September) | 2^nd^ peak/”2^nd^ wave”  (from October on) |
| Very large |  |  |  |  |
| Large |  |  |  |  |
| Medium |  |  |  |  |
| Small |  |  |  |  |
| Very small |  |  |  |  |
| How do you estimate the **communication effort** before and at each stage of the pandemic? | | | | |
|  | Before the pandemic  (January/February) | 1^st^ peak/”1^st^ wave”  (March/April) | Intermediate stage  (May until September) | 2^nd^ peak/”2^nd^ wave”  (from October on) |
| Very large |  |  |  |  |
| Large |  |  |  |  |
| Medium |  |  |  |  |
| Small |  |  |  |  |
| Very small |  |  |  |  |
| How do you assess the **need for information** before and at each stage of the pandemic? | | | | |
|  | Before the pandemic  (January/February) | 1^st^ peak/”1^st^ wave”  (March/April) | Intermediate stage  (May until September) | 2^nd^ peak/”2^nd^ wave”  (from October on) |
| Very large |  |  |  |  |
| Large |  |  |  |  |
| Medium |  |  |  |  |
| Small |  |  |  |  |
| Very small |  |  |  |  |

| How would you rate your **work atmosphere** before and at each stage of the pandemic? | | | | |
| --- | --- | --- | --- | --- |
|  | Before the pandemic  (January/February) | 1^st^ peak/”1^st^ wave”  (March/April) | Intermediate stage  (May until September) | 2^nd^ peak/”2^nd^ wave”  (from October on) |
| Very good |  |  |  |  |
| Good |  |  |  |  |
| Medium |  |  |  |  |
| Bad |  |  |  |  |
| Very bad |  |  |  |  |
| How do you assess the **quality of care for palliative care patients in nursing homes** before and at each stage of the pandemic? | | | | |
|  | Before the pandemic  (January/February) | 1^st^ peak/”1^st^ wave”  (March/April) | Intermediate stage  (May until September) | 2^nd^ peak/”2^nd^ wave”  (from October on) |
| Very good |  |  |  |  |
| Good |  |  |  |  |
| Medium |  |  |  |  |
| Bad |  |  |  |  |
| Very bad |  |  |  |  |
| How do you assess the **quality of care for palliative care patients at home** before and during the different phases of the pandemic? | | | | |
|  | Before the pandemic  (January/February) | 1^st^ peak/”1^st^ wave”  (March/April) | Intermediate stage  (May until September) | 2^nd^ peak/”2^nd^ wave”  (from October on) |
| Very good |  |  |  |  |
| Good |  |  |  |  |
| Medium |  |  |  |  |
| Bad |  |  |  |  |
| Very bad |  |  |  |  |
| How do you assess the **quality of care for palliative care patients in hospices** before and during the individual phases of the pandemic? | | | | |
|  | Before the pandemic  (January/February) | 1^st^ peak/”1^st^ wave”  (March/April) | Intermediate stage  (May until September) | 2^nd^ peak/”2^nd^ wave”  (from October on) |
| Very good |  |  |  |  |
| Good |  |  |  |  |
| Medium |  |  |  |  |
| Bad |  |  |  |  |
| Very bad |  |  |  |  |
| How do you assess the **quality of care for palliative care patients in institutions of integration assistance** before and during the individual phases of the pandemic? | | | | |
|  | Before the pandemic  (January/February) | 1^st^ peak/”1^st^ wave”  (March/April) | Intermediate stage  (May until September) | 2^nd^ peak/”2^nd^ wave”  (from October on) |
| Very good |  |  |  |  |
| Good |  |  |  |  |
| Medium |  |  |  |  |
| Bad |  |  |  |  |
| Very bad |  |  |  |  |

# DESCRIPTIVE DESCRIPTION OF THE SAPV TEAMS PART 2

At the end of the questionnaire, we ask you to provide general information describing your SAPV team.

| Data on the organization |  |
| --- | --- |
| How many doctors provide SAPV services in your SAPV team? | - ______Number |
| How many of these doctors work in SAPV with at least 50% of a full-time position? | - ______ Number |
| Through which structures are doctors employed to provide SAPV services in your team? | - the SAPV team itself - a hospital - a care service - others |
| How many nurses provide SAPV services on your SAPV team? | - ______ Number |
| How many of these nurses work in SAPV with at least 50% of a full-time position? | - ______ Number |
| Through which structures are nurses employed to provide SAPV services in your team? | - the SAPV team itself - a hospital - a care service - others |
| How is your SAPV organized? | - Independent organization (association, gGmbH...) - Connected to a clinic/hospital - Connected to another sponsoring organization, namely: |

| Question | Input content |
| --- | --- |
| Do you have any other comments on the questionnaire? |  |
