# Supplementary material for: National strategy for palliative care of severely ill and dying people and their relatives in pandemics (PallPan) in Germany - study protocol of a mixed-methods project
Source: BMC Palliat Care. 2022 Jan 13;21:10. doi: 10.1186/s12904-021-00898-w (PMC8756412; doi:10.1186/s12904-021-00898-w)
Supplement: Supplementary file 9 — Additional file 9: Supplementary file WP4. Interview guide PC Hospital. [file 12904_2021_898_MOESM9_ESM.docx]

**PallPan: "National strategy for palliative care in pandemic times"**

**WP 4: Generalist inpatient palliative care Interview Guide**

| **Introduction** |
| --- |

- Introduction:

I am pleased that today we found the time to meet for a focus group interview with the topic “inpatient generalist palliative care during pandemic times”. The overall goal of the PallPan study is to describe and analyse the experiences, challenges and possible solutions in hospitals concerning the care for severely ill, dying and deceased patients (with/without COVID-19) and their relatives or bereaved. In todays conversation we focus on inpatient generalist palliative care in … (intensive care units/ isolation wards/ special wards e.g. geriatric unit or psychiatric ward) at … (university hospitals/ hospitals excluding university hospitals).
Thank you very much for taking part in this interview. You contribute to the success of our project.

- Data privacy:

As we announced in writing already, we would like to record our discussion to be able to evaluate and analyse it afterwards. There will be no publishing of any video or audio material. Do you agree to be recorded from now onwards? (if yes – start recording) Due to data privacy reasons I need to ask you again after starting the recording if you agree to be recorded. Thank you!

- Presentation of the planned proceedings and communication rules:

Before we start I would like to share some thoughts about the proceeding and communication rules: We plan on discussion for about 60-90 minutes and we would like to learn as much as possible from your perspective. We are going take a backseat during the conversation, take notes and only asks a few questions where necessary. Otherwise I would like to invite you to talk with one another. Please try to organize yourselves and maybe already discuss with each other, add points to what was already said or ask questions. Speak your mind if you agree or disagree with what was said or just share your experiences. Please try not to get off the subject and let each other finish. Thereby it is easier for us to take notes and in the end evaluate the interview. My co-moderator supports me with taking notes and comprising interim results.

- Introduction of participants:

I would like to start with a quick introduction round. Therefore I am going to present myself: My name is …. Now I would ask you to introduce yourself with two small sentences.

| **INTENSE CARE UNIT** |
| --- |
| **Block 1: Experiences/ Everyday working life** |

What did your everyday professional life look like during the last 6 months since the beginning of the SARS-CoV2 pandemic with regards to severly ill, dying and deceased patients and their relatives, also in comparison to pre-pandemic times? (Which experiences did you have, think about the different phases of lockdown and relaxation?)

| **Substantial aspects** | **Maintenance questions** | **Specific questions** |
| --- | --- | --- |
| - Care pathways - Supporting opportunities - Rituals - Therapy goal setting - Inclusion of relatives - Dealing with advance directives and power of attorney - Care in the context of dying and after death | - Which aspects comprise your everyday professional life with regard to severely ill, dying and deceased patients in addition to what was already mentioned? - In which condition are the patients you care for in your everyday professional life and did differences occur due to the pandemic situation? - Which role do relatives of patients play during your everyday working life and to which extent did it change due to the pandemic? - Which role does and did palliative care play in your everyday working life? | - Are there supporting opportunities and care pathways specifically with regard to severely ill, dying and deceased patients? - In which way did the therapy goal setting change during the pandemic? - Which role did advance directives and power of attorney play and did it change significantly? - How did the contact with relatives and loved ones turn out? - What does the care in the context of dying and after death look like? - Did you always feel like being in control of the situation? - Have there been less resources of specialized palliative care or were these requested less frequently than before? |

| **Block 2: Challenges** |
| --- |

Which challenges did you face during the last 6 months since the beginning of the SARS-CoV-2 pandemic? To what extent did these challenges differ in comparison to pre-pandemic times?

| **Substantial aspects** | **Maintenance questions** | **Specific questions** |
| --- | --- | --- |
| - Resource scarcity (stuff, staff, space, systems) - Difficult conversations (Goal of care) - Triage - Personal, psychological distress   Reasons:   - Guidelines of the institution/ facility - Legal/ political prescribed measures to control the pandemic - Lack of protective equipment | - What else do you link to challenges in the context of the pandemic? - Who else do you link to challenges (persons or institutions)? - What makes your job challenging specifically regarding the care of severely ill, dying and deceased patients? | - How did you perceive working with relatives of the severely ill, dying and deceased patients? - How did you perceive the resource allocation? - Which challenges did occur due to the allocation of shortly trained intensive care staff? - How did you perceive the guidelines (e.g. visiting restrictions) of different entities? - How did you personally feel? Did you feel safe, protected and supported during your work? |

| **Block 3: Solution approaches** |
| --- |

What solutions were developed in your facility/institution/ward to overcome these challenges? Would you have wished for more support or solutions and from whom and what specifically?

| **Substantial aspects** | **Maintenance questions** | **Specific questions** |
| --- | --- | --- |
| - Guidelines - Concepts - Altered work flows/ structures - Telemedicine (relatives) | - Which thoughts do you connect to solution approaches? - Would you describe this approach more precisely for me? - Did you note solutions in your facility which can be used during another pandemic? | - What could a solution look like? What could it comprise? - Can you fall back on specific solutions? Is there specific support in your facility (specialized palliative care, health care ethics committee)? - To what extent did you have solutions with regard to dealing with relatives? - What would you specifically wish for regarding solutions? Did you yourself or in your professional environment develop these kind of strategies? |

| **ISOLATION WARD/ COVID-WARD** |
| --- |
| **Block 1: Experiences/ Everyday working life** |

What did your everyday professional life look like during the last 6 months since the beginning of the SARS-CoV2 pandemic with regards to severly ill, dying and deceased patients and their relatives, also in comparison to pre-pandemic times? (Which experiences did you have, think about the different phases of lockdown and relaxation?)

| **Substantial aspects** | **Maintenance questions** | **Specific questions** |
| --- | --- | --- |
| - Care pathways - Supporting opportunities - Rituals - Therapy goal setting - Inclusion of relatives - Dealing with advance directives and power of attorney - Care in the context of dying and after death | - Which aspects comprise your everyday professional life with regard to severely ill, dying and deceased patients in addition to what was already mentioned? - In which condition are the patients you care for in your everyday professional life and did differences occur due to the pandemic situation? - Which role do relatives of patients play during your everyday working life and to which extent did it change due to the pandemic? - Which role does and did palliative care play in your everyday working life? | - Are there supporting opportunities and care pathways specifically with regard to severely ill, dying and deceased patients? - In which way did the therapy goal setting change during the pandemic? - Which role did advance directives and power of attorney play and did it change significantly? - How did the contact with relatives and loved ones turn out? - What does the care in the context of dying and after death look like? - Did you always feel like being in control of the situation? - How is the personnel capacity on your ward? Were and is there enough staff on the isolation ward? Was the organisation easy to prepare this kind of ward? - Does your ward as a kind of special ward consult with other wards/ clinics/ institutions? |

| **Block 2: Challenges** |
| --- |

Which challenges did you face during the last 6 months since the beginning of the SARS-CoV-2 pandemic? To what extent did these challenges differ in comparison to pre-pandemic times?

| **Substantial aspects** | **Maintenance questions** | **Specific questions** |
| --- | --- | --- |
| - Resource scarcity (stuff, staff, space, systems) - Difficult conversations (Goal of care) - Triage - Personal, psychological distress   Reasons:   - Guidelines of the institution/ facility - Legal/ political prescribed measures to control the pandemic - Lack of protective equipment | - What else do you link to challenges in the context of the pandemic? - Who else do you link to challenges (persons or institutions)? - What makes your job challenging specifically regarding the care of severely ill, dying and deceased patients? | - How did you perceive working with relatives of the severely ill, dying and deceased patients? - How did you perceive the resource allocation? - How did you perceive the guidelines (e.g. visiting restrictions) of different entities? - Have Patientenverfügungen and Vorsorgevollmachten been special challenges? - Do conversations with patients/ relatives become more difficult or different due to the public insecurities or the rapid decline of the patients condition? - How did you personally feel? Did you feel safe, protected and supported during your work? |

| **Block 3: Solution approaches** |
| --- |

What solutions were developed in your facility/institution/ward to overcome these challenges? Would you have wished for more support or solutions and from whom and what specifically?

| **Substantial aspects** | **Maintenance questions** | **Specific questions** |
| --- | --- | --- |
| - Guidelines - Concepts - Altered work flows/ structures - Telemedicine (relatives) | - Which thoughts do you connect to solution approaches? - Would you describe this approach more precisely for me? - Did you note solutions in your facility which can be used during another pandemic? | - What could a solution look like? What could it comprise? - Can you fall back on specific solutions? Is there specific support in your facility (specialized palliative care, health care ethics committee)? - Can you name used strategies which you would recommend? - To what extent did you have solutions with regard to dealing with relatives? - What would you specifically wish for regarding solutions? Did you yourself or in your professional environment develop these kind of strategies? |

| **Exceedlingly burdened** |
| --- |
| **Block 1: Experiences/ Everyday working life** |

What did your everyday professional life look like during the last 6 months since the beginning of the SARS-CoV2 pandemic with regards to severly ill, dying and deceased patients and their relatives, also in comparison to pre-pandemic times? (Which experiences did you have, think about the different phases of lockdown and relaxation?)

| **Substantial aspects** | **Maintenance questions** | **Specific questions** |
| --- | --- | --- |
| - Care pathways - Supporting opportunities - Rituals - Therapy goal setting - Inclusion of relatives - Dealing with advance directives and power of attorney - Care in the context of dying and after death | - Which aspects comprise your everyday professional life with regard to severely ill, dying and deceased patients in addition to what was already mentioned? - In which condition are the patients you care for in your everyday professional life and did differences occur due to the pandemic situation? - Which role do relatives of patients play during your everyday working life and to which extent did it change due to the pandemic? - Which role does and did palliative care play in your everyday working life? | - Are there supporting opportunities and care pathways specifically with regard to severely ill, dying and deceased patients? - Did you experience new and different situations when dealing with patients compared to before the pandemic? - Did the behaviour or health condition of your patients drastically change due to the pandemic? - How was the experience in particular with patients suffering from dementia or having organic brain changes? - How was the influence of hygienic measures on the patients (face masks, plexiglass shields)? - Did your everyday working life change due to the visiting restrictions and regulations? - Have there been less resources of specialized palliative care or were these requested less frequently than before? - Could you notice a change regarding the relocation of patients e.g. discharge to a care home or admission to the hospital from care homes |
| **Block 2: Challenges** | | |

Which challenges did you face during the last 6 months since the beginning of the SARS-CoV-2 pandemic? To what extent did these challenges differ in comparison to pre-pandemic times?

| **Substantial aspects** | **Maintenance questions** | **Specific questions** |
| --- | --- | --- |
| - Resource scarcity (stuff, staff, space, systems) - Difficult conversations (Goal of care) - Triage - Personal, psychological distress   Reasons:   - Guidelines of the institution/ facility - Legal/ political prescribed measures to control the pandemic - Lack of protective equipment | - What else do you link to challenges in the context of the pandemic? - Who else do you link to challenges (persons or institutions)? - What makes your job challenging specifically regarding the care of severely ill, dying and deceased patients? | - What lead to challenges due to the pandemic regarding dealing with severely ill and dying patients? - In how far did guideline (e.g. visiting restrictions, obligation to wear a mask) lead to challenges when dealing with severely ill and dying patients? - How did patients react to quarantine situations or did they even realize these? - How was the discharge management designed? Was a proper discharge planning possible? - In how far does social isolation influence or concern your patients more than others? - Could your recognize an acute decline in the patients condition which lead to challenging situations? - How did you and your patients perceive the loss or restriction of visitations? - How did you personally feel? Did you feel safe, protected and supported during your work? |

| **Block 3: Solution approaches** |
| --- |

What solutions were developed in your facility/institution/ward to overcome these challenges? Would you have wished for more support or solutions and from whom and what specifically?

| **Substantial aspects** | **Maintenance question** | **Specific questions** |
| --- | --- | --- |
| - Guidelines - Concepts - Altered work flows/ structures - Telemedicine | - Which thoughts do you connect to solution approaches? - Would you describe this approach more precisely for me? - Did you note solutions in your facility which can be used during another pandemic? | - Can you fall back on specific solutions? Is there specific support in your facility? - Did you receive support for the discharge management or assessment of new intakes? If yes what did it look like? - Did you find alternative ways to simplify working with your patients (patients suffering from dementia do not understand wearing face masks or to keep a certain distance)? - Can you name used strategies which you would recommend? - What would you specifically wish for regarding solutions? Did you yourself or in your professional environment develop these kind of strategies? |

| **End** |
| --- |

I would like to close the focus group interview now if everyone agrees.

My colleague has gathered the most important topics and statements. We can listen to these now and if there is anything to add or to underline very important aspects please share your thoughts with us.

Thank you again for your time and goodbye!
