# Supplementary material for: National strategy for palliative care of severely ill and dying people and their relatives in pandemics (PallPan) in Germany - study protocol of a mixed-methods project
Source: BMC Palliat Care. 2022 Jan 13;21:10. doi: 10.1186/s12904-021-00898-w (PMC8756412; doi:10.1186/s12904-021-00898-w)
Supplement: Supplementary file 10 — Additional file 10: Supplementary file WP4. Online Survey PC Hospital. [file 12904_2021_898_MOESM10_ESM.docx]

**PallPan: "National strategy for palliative care in pandemic times"**

**WP 4: Generalist inpatient palliative care Online Survey**

Online questionnaire to assess the care situation of seriously ill and dying patients with and without COVID-19 and their relatives in hospital during the first wave of the COVID-19 pandemic (Mach-May 2020)

A. Patients volume and quality of care.

We aim to provide an overview of the qualitative and quantitative changes in patient volume during the first wave of the COVID-19 pandemic (March-May 2020)

Please answer the following questions:

1. Have patients with COVID-19 been cared for in the hospital you work?

Yes/ no/ not assessable

If answered with yes:

- 1. Please specify your answer:
  2. The hospital was located in a hotspot area during the designated period and had many COVID-19 patients.
  3. The hospital was located in a hot-spot area during the designated period, but had only a few COVID-19 patients
  4. The hospital was not located in a hot spot area during the designated period, but admitted COVID-19 patients from other hot spot areas
  5. The hospital was not located in a hot-spot area, but had many COVID-19 patients of its own
  6. The hospital was not located in a hot-spot area and had only a few COVID-19 patients of its own.

1. Have patients with COVID-19 been cared for in your professional field of activity?

Yes / no / not assessable

If answered with yes:

- 1. Estimate how many:
  2. Free nomination

1. To what extent has hospital occupancy changed compared to before the COVID-19 pandemic?

Considerably increased / increased / remained almost the same / decreased / considerably decreased / not assessable

1. To what extent has number of seriously ill and dying patients changed compared to before the COVID-19 pandemic?

Considerably increased / increased / remained almost the same / decreased / considerably decreased / not assessable

1. To what extent has quality of care for the seriously ill and dying with and without COVID-19 changed compared with before the COVID-19 pandemic?

Considerably improved / improved / remained almost the same / deteriorated / considerably deteriorated / not assessable

B. Symptom relief

1. The following questions about symptom relief are primarily for physicians and nurses. If you do not belong to this group, feel free to skip ahead to the next block of questions. (skip to C. Decision making)

Dying people often experience shortness of breath, anxiety and restlessness. What medications and measures do you use to relieve those symptoms?

1. What medication and measures do you use to relieve shortness of breath?
   1. Administration of oxygen
   2. Administration of diuretics, e.g. Furosemide or Toresamide
   3. Administration of opioids, e.g. Morphine or Hydromorphon
   4. Administration of benzodiazepines, e.g. Lorazepam or Midazolam
   5. Positioning of patients in their beds
   6. Rubbing, e.g. with essential oils
   7. Involvement of other therapists, e.g. respiratory therapists, physical therapists, occupational therapists
   8. Other, namely:
   9. Free nomination
   10. Not assessable
2. What medication and measures do you use to relieve anxiety and restlessness?
   1. Administration of benzodiazepines, e.g. Lorazepam or Midazolam
   2. Administration of opioids, e.g. Morphine or Hydromorphon
   3. Administration of neuroleptics, e.g. Pipamperone or Risperidone
   4. Involvement of family members
   5. Rubbing, e.g. with essential oil
   6. Sitting guard
   7. Soothing music
   8. Basal stimulation
   9. Involvement of other therapists, e.g. relaxation therapy
   10. Other, namely:
   11. Free nomination
   12. Not assessable
3. Were there shortages in the procurement of medicines during the first wave of the COVID-19 pandemic (March-May 2020)?

Yes / no / not assessable

If answered with yes:

- 1. Please explain your statement:
  2. Free nomination

C. Decision making

Making patient focused decisions in the care of the seriously ill and dying with and without COVID-19 may have changed during the first wave of the COVID-19 pandemic (March-May 2020).

The following questions relate to the changes in your professional field of activity:

1. Has the approach to therapy goal finding changed?

Yes / no / not assessable

If answered with yes:

- 1. Please explain:
  2. Free nomination

1. Did the seriously ill and dying patients express increased interest in the creation of documents for self-determined health care planning such as living wills and / or powers of attorney?

Yes / no / not assessable

1. Did the seriously ill and dying patients express increased interest in reviewing or modifying existing documents for self-determined health care planning?

Yes / no / not assessable

1. Were there any written instructions with regard to:

Each sub item with the following answer options:

Yes, already existed before / yes, was new introduced / no, also did not exist before / no, was suspended / not assessable

- 1. Documentation of the therapy goal
  2. Setting of escalation and limitation of therapy
  3. Dealing with patients with dementia
  4. Dealing with the dying
  5. Dealing with the deceased
  6. Support for relatives
  7. Support for the bereaved
  8. Grief counseling
  9. Other concepts:
  10. Free nomination

1. How often had to be weighed between infection control and:

Each sub item with the following answer options:

Very often / often / rarely / never / not assessable

- 1. Quality of Life
  2. Quality of Death
  3. Needs of relatives and bereaved

1. In your opinion, were these trade-offs appropriate with respect to the care of the seriously ill and dying? (Question relates to previous question)

Yes / no / not assessable

D. Psychosocial issues of patients, relatives and employees

During the first wave of the COVID-19 pandemic (March-May 2020), there were also changes in terms of psychosocial issues among the seriously ill and dying with and without COVID-19, their relatives, and the staff in your professional field of activity. We want to capture this in the next questions:

1. How has quality changed in regards to:

Each sub item with the following answer options:

Considerably improved / improved / remained almost the same / deteriorated / considerably deteriorated / not assessable

- 1. Emotional support of patients and their relatives
  2. Addressing the problems and concerns of multimorbid patients
  3. Addressing the problems and concerns of patients with dementia
  4. Consideration of the patient´s will when determining the indication for e.g. intensive care treatment

1. Were there exemptions for visiting dying patients?

Yes / no / not assessable

If answered with yes:

17.1. Only COVID-19 negative patients / only COVID-19 positive patients / both

1. During the care of dying patients with and without COVID-19, which of the following options existed in your daily work routine?
   1. Protection of privacy
   2. Rooming-in from relatives
   3. Providing a single room during the dying phase
   4. Use of a farewell room
2. Which digital services for communication between the seriously ill and the dying and their relatives existed in your facility?
   1. Telephones
   2. Access to the internet
   3. Tablet PC
   4. Smartphone
   5. Videoconferences
   6. Other, namely:
   7. Free nomination
   8. No special offers
3. What services existed to support relatives in taking leave of the deceased?

Each sub item with the following answer options:

Yes, already existed before / yes, was new introduced / no, also did not exist before / no, was suspended / not assessable

- 1. Structured conversation offers for the bereaved by the physicians
  2. Structured conversation offers for the bereaved by the nursing staff
  3. Religious rituals and conversations
  4. Psychooncological / psychotherapeutic conversations
  5. Accompaniment by hospice volunteers
  6. Giving information about grief counseling (e.g. flyer)
  7. Cards of condolence
  8. Other, namely:
  9. Free nomination

1. Did the operational management /crisis team support you in making exceptions for dying and farewell situations?

Yes / no / partially / not assessable

If answered with partially:

- 1. Please explain your statement:
  2. Free nomination

E. Collaboration

In the following section, we will discuss collaboration with palliative care professionals and other disciplines in the care and support of the seriously ill and dying with and without COVID-19 in your professional field of activity during the first wave of the COVID-19 pandemic (March-May 2020):

1. How has interdisciplinary collaboration changed in general?

Considerably improved / improved / remained almost the same / deteriorated / considerably deteriorated / not assessable

1. Were palliative care professionals actively involved in the care of the seriously ill and dying?

Yes, already existed before / yes, was new introduced / no, also did not exist before / no, was suspended / not assessable

If answered with no, was suspended:

- 1. Please explain your statement:
  2. Free nomination

1. What form did the involvement take? (Question relates to previous question)
   1. Consultative function via telephone or video regarding patients with COVID-19
   2. Consultative function via telephone or video regarding patients without COVID-19
   3. Support at the bedside of patients with COVID-19
   4. Support at the bedside of patients without COVID-19
   5. Not assessable
2. Was the transfer of seriously ill and dying patients from your professional field of activity to a palliative care unit possible?

Yes / no / not assessable

If answered with no:

- 1. Please explain the reasons:
  2. Free nomination

1. Which patients were transferred to a palliative care unit? (Question relates to previous question)

With and without COVID-19 / only without COVID-19 / not assessable

1. Was the discharge of the seriously ill and dying from your professional field of activity to their home, nursing home, or hospice possible?

Yes / partially / no / not assessable

If answered with partially:

- 1. Please explain your statement:
  2. Free nomination

F. Approaches/Solutions and resources

The following questions relate to your experiences caring for seriously ill and dying patients with and without COVID-19 in your professional field of activity during the first wave of the COVID-19 pandemic (March-May 2020). These may provide important approaches for managing patients in future pandemic situations.

1. If you think of the worst-case scenario, i.e. a situation in which you would have to take care for a large number of seriously ill and dying patients at the same time, what would be helpful?
   1. Access to additional wards and premises
   2. Recruitment of additional, medically trained, staff
   3. Availability of standardized pandemic kits with medications and materials for

symptom control

- 1. Written instructions on how to deal with the dying, the deceased and their relatives /

bereaved

- 1. Other, namely:
  2. Free nomination

1. Would additional services from palliative care professionals be helpful?

Yes / no / not assessable

If answered with yes:

1. Which services would be helpful?
   1. Hospital rounds via Video / video visits
   2. Topic related short presentations
   3. Advanced training courses
   4. easily available information
   5. Further written recommendations for action
   6. Not assessable
2. In your opinion, how important are the following measures for the care of dying patients with and without COVID-19?

Each sub item with the following answer options:

Very important / important / less important / unimportant / cannot judge

- 1. Visits from relatives during the phase of dying
  2. Contact with therapists
  3. Accompaniment of socially isolated dying patients by volunteers
  4. Taking leave of the deceased
  5. Digitally supported collaboration with palliative care professionals, e.g., video visits
  6. Further training on how to deal with dying patients with and without infectious

diseases

- 1. Increased exchange with other clinics (standards and best-practice examples)
  2. Relief of the employees e.g. by rituals, individual conversations or supervision
  3. Other, namely:
  4. Free nomination

1. What positive experiences did you have during the first wave of the COVID-19 pandemic (March-May 2020) with regard to the care and support of the seriously ill and dying?

- Free nomination

1. Based on your experience in spring 2020, what is particularly important for you in the current pandemic peak period (since November 2020) to ensure dignified care of your seriously ill and dying patients?

- Free nomination

G. Personal stresses

1. Hospital staff working close to patients were confronted with various stress factors during the first wave of the COVID-19 pandemic (March-May 2020). Select the most important ones
   1. Loneliness of patients due to visit restrictions
   2. Lack of daily structure for patients
   3. Psychological burden on the seriously ill and dying
   4. Psychological burden on relatives and the bereaved
   5. Increased workload
   6. More sick leave in the team
   7. Private multiple stresses
   8. Implementation of stricter hygiene regulations
   9. Respecting distance rules to patients
   10. Difficulty in collecting the deceased
   11. Difficulty / impossibility of taking leave at the bedside of the dying / deceased
   12. Concern about own infection
   13. Implementation of visiting regulations for relatives
   14. Difficulty in communication with relatives
   15. Difficulty in communication with other hospitals
   16. Increase in conflicts within the team
   17. Other burdens, namely:
   18. Free nomination
   19. There were no particular burdens

H. Personal data

1. How old are you?
2. What gender are you?

Female / male / diverse

1. To which profession do you belong?

- Physician

o Resident physician

o specialist/superior physician

o Senior physician or head physician

- Nurse Practitioner

o Non-managerial position

o Ward manager

o Nursing service management

o Nursing director

- Psychologist/ Psychooncologist/ Psychotherapist

- Social service/ discharge management

- Pastoral care

- Physiotherapist/ logo therapist/ respiratory therapist

- 1. Other, namely:
  2. Free nomination

1. In which medical specialty do you predominantly work?
2. Years of work experience?

Up to 5 years / 6-10 years / 11-25 years / longer than 25 years

1. Do you have an additional qualification/post-graduate qualification in palliative medicine /palliative care?

Yes / no / not assessable

1. How many patients in the final phase have you approximately accompanied in the past year (2019)?

None / less than 10 / 11-50 / 51-100 /more than 100

1. To which level of care does the hospital in which you work belong?

Primary and standard care hospital / Specialized hospitals / hospitals with priority care / Maximum care, non-university hospital / Maximum care, university hospital / Not assessable

1. On which ward do you work?

- Normal ward

- Palliative ward

- Specialized ward:

o Intensive care unit

o Admission ward

o Isolation ward

- Interdepartmental activity

- 1. Other, namely:
  2. Free nomination
