# Supplementary material for: National strategy for palliative care of severely ill and dying people and their relatives in pandemics (PallPan) in Germany - study protocol of a mixed-methods project
Source: BMC Palliat Care. 2022 Jan 13;21:10. doi: 10.1186/s12904-021-00898-w (PMC8756412; doi:10.1186/s12904-021-00898-w)
Supplement: Supplementary file 11 — Additional file 11: Supplementary file WP5. Interview guide SPC Inpatient. [file 12904_2021_898_MOESM11_ESM.docx]

**WP5 Interview Guide Specialist Palliative Inpatient Care**

| Interviewer: |  |
| --- | --- |
| Interview code/-number: |  |
| Position and gender: |  |
| Date: |  |
| Start of interview (time): |  |
| Telephone-/video interview: |  |

"Thank you very much for taking your time for this interview! We are conducting a study on the impact of COVID-19 on inpatient hospice and palliative care for patients/guests and relatives. We are also interested in how your team is doing in maintaining care. With the help of these expert interviews, we would like to gain an insight into the challenges of inpatient care/support, admission and transferral. Thus, the core questions of this qualitative analysis are: How can hospice and palliative care continuity be guaranteed under pandemic conditions? Where did you and your team develop good solutions? What barriers continue to emerge in everyday life?"

## **Adaption of core processes**

## **A1) General structures**

1. What requirements did the COVID-19 pandemic impose on you and your team?

- *ask if necessary:* from the federal government/state; Robert Koch Institute; direction; (local) health authorities
- Were these requirements feasible? If so, how quickly?
- Which requirements pushed you to your limits?
- Can you give an example to illustrate the problems of implementation?

1. Which areas/aspects that are important for care did you plan proactively together with your team?

- What / how much was delegated from a higher level?
- In which areas did you have a say?
  - for you as a leader vs. for your team

1. In which areas would you have liked more support or information? And if so, whence? (contact persons and accessibility)
2. *[hospital palliative care support teams and palliative care units]:* Did you participate in committees? If so, in which committees were you automatically involved, in which did you have to point out the involvement of palliative care?

## **A2) Working with COVID-19 infected persons**

**Patients/Guests and relatives**

1. Did you have patients/guests or relatives with COVID-19? Were there any suspected cases of COVID-19?

- Was there a standard test procedure for patients/guests before or at admission and during their stay?
- How do you handle this today?
- Was there an admission freeze at your facility?
- [hospital palliative care support teams]: Did you have more or less consultation requests during this time? Did the requests come from the usual wards or did new ones come in?

1. How do you deal with COVID-19 infected persons or with suspected cases?

**Health care staff**

1. Did you have positively tested staff members or suspected cases in your team?

- If so, what measures were taken?

1. Did you develop strategies for maintaining care in case of infected staff or quarantine for staff? (e. g. clustering, teleworking; short-time working; separation of workspaces)
2. Did you have the possibility to be supported by additional health care staff in case of staff shortages?

- If so, where were they recruited from? (e. g. medical students, retired physicians etc.)
- Approximately what percentage of staff were you able to keep during the pandemic period?

1. In contrast to the first lockdown, are there any differences in the way you treat COVID-19 infected persons or suspected cases?

## **A3) Impact on the care of patients/guests and relatives**

1. What impact did COVID-19 have on the care of patients/guests with palliative care needs who had already been admitted or were to be admitted?
2. Did you have any visitor regulations?

- If so, what did this mean for patients/guests and their relatives?
- What impact did this have on staff?

1. Which (inter- and multidisciplinary) services were discontinued or offered in a different form due to the pandemic? (co-operation partners, e. g. humour interventions, pastoral care etc.)

- Were the volunteers still allowed to come into your facility? If not, did you miss their contribution?
- Are there any differences to the first lockdown? If so: Can you please give us concrete examples?

1. Did you create alternative offers for contacting?

- If so, which ones? (face-to-face or virtual)

1. Did you request/were given exceptions for your facility?

- How did you experience the special regulations made for relatives of dying people "Saying goodbye to patients/deceased persons in times of Covid-19"?
- (How) do these restrictions change end-of-life care?
- Which of them do you think could be permanent?

1. What did you have to consider in order to be able to continue provision of care in your facility?

- concerning the premises (insufficient rooms/equipment)
- concerning bed occupancy
- concerning hygiene measures etc.

1. Were there fewer patients/guests during the pandemic and what impact did this have?

## **A4) Team structures and -processes**

1. What operational support & benefits were offered to staff?

- testing
- extra material (e. g. face masks; hand lotion; presents (appreciation); bonuses
- information
- emergency care for children or care-dependent relatives

1. Did you establish additional measures and offers to motivate and strengthen staff members?
2. Have there been changes in the rituals you offer to staff in your facility? (due to distance regulations etc.)?
3. When you think about your team, what fears & worries were you faced with as a leader?

- e. g. fear of infection

1. Were there trainings and induction periods/work interruptions (e.g. online training)?

- if so, what did these look like?
- if not, would you have liked some for your team?
  - which ones?

1. Did any of your employees work from home?

- Did this work well or were there difficulties with accessibility etc.?
- If so, can you please give us some concrete examples?

1. In case of a further/recurring pandemic: Are there any proposals for personnel restructuring - resulting from the current experiences?

## **Resources and stressors**

1. Were there any areas where shortages occurred?

- protective equipment
- disinfectants
- face masks
- acrylic glass
- medication

1. Did measures exist to prevent a possible shortage?
2. Did the pandemic have an impact on the financial situation of your facility?

- If so, which one?

1. Did you have a hygiene concept?

- Is it recorded in writing?
  - If so, could you make it available to us?
  - If not, could you please briefly list the most important points?

1. How did the prescribed hygiene and protection measures affect your work? (This refers to additional work, higher workload etc.)
2. To what extent did they affect your work with regard to the care of patients/guests? (Wearing masks; counselling; touch; closeness/distance)
3. To what extent did they affect your work with relatives?
4. To what extent did they affect teamwork?

- increased hygiene measures
- workload
- amount of work
- time pressure
- more difficult counselling of relatives

1. Has anything changed in your work processes and areas of responsibility because of the pandemic?

- What went well and what did not go well?
- What challenges do you still face? (critical incidence)

## **Networking/collaboration**

1. How did admissions [hospital: discharges/transfers] work during the pandemic period?
2. Was there any extra effort in communication during admission/counselling [hospital: and discharge]?

- If so, with whom, where, why? (patients/guests, relatives, staff, (crisis) management etc.)

1. How did the communication with other professions/teams work?

- interface management
- interdisciplinary cooperation
- How is this related to the pandemic?

1. How did the cooperation with funeral homes work?

- Were there any reservations / fears / worries?

## **Final**

1. Did there any areas/aspects occur that you now know "would have been good to consider in the future"?

- If so, which ones?
- Could you please give us a concrete example of this?

1. If you could wish for something for inpatient palliative care in pandemic times. What would that be?

## **Demographic data and closure**

*Finally, if you agree, I would like to collect a few demographic details that will help us in our data processing:*

*o What is your function in your facility?*

*o City/rural*

*o How many beds/occupancy places are there in your facility?*

*o For palliative care units: Admission freeze/restructuring (closed/restructured/keep everything as before).*

*Thank you for your time!*

*In case of emotional stress, we offer you the opportunity to have supervisory conversations with appropriately trained specialists from the Department of Palliative Medicine, University Hospital Bonn.*
