# Supplementary material for: National strategy for palliative care of severely ill and dying people and their relatives in pandemics (PallPan) in Germany - study protocol of a mixed-methods project
Source: BMC Palliat Care. 2022 Jan 13;21:10. doi: 10.1186/s12904-021-00898-w (PMC8756412; doi:10.1186/s12904-021-00898-w)
Supplement: Supplementary file 13 — Additional file 13: Supplementary file WP5. Online Survey PC Support Teams. [file 12904_2021_898_MOESM13_ESM.docx]

**WP 5 Online Survey Hospital Palliative Care Support Teams**

**Palliative care in pandemic times (hospital palliative care support teams)**

Dear participants,

Even in times of a pandemic, the care of seriously ill and dying people is an important task, regardless of whether the patients suffer from a Covid 19 infection or another serious illness. In the current pandemic situation, the care of seriously ill and dying people was difficult and only possible with restrictions. There are indications that appropriate palliative care was not possible at times – neither for the seriously ill and the dying, nor for those close to them who wanted to say goodbye. The project ‘Palliative care in times of pandemics (PallPan)’ under the coordination of Professor Claudia Bausewein (LMU Munich) and Professor Steffen Simon (UK Cologne) is dedicated to this still relevant topic.

At the University Hospital Bonn, we are working at the Department of Palliative Medicine on evidence-based recommendations for action in the field of specialist inpatient palliative care (SSPV). Among other things, this nationwide anonymous online survey of palliative care units, hospital palliative care support teams and inpatient hospices serves this purpose. Guideline-based interviews and an anonymised analysis of the hygiene plans of SSPVs will deepen the insight in challenges and need for improvement in dealing with a pandemic situation.

Please also read the detailed project description, information on data protection as well as your contact persons for questions on data protection.

Here is the file to download.

By continuing with the survey, you agree to participate in this study, which has been approved by our ethics committee (Ethikvotum Universitätsklinikum Bonn, No. 480/20). If you have any questions, please do not hesitate to contact us. Thank you for your interest and participation in this study.

Yours sincerely,

Gülay Ateș, Katja Maus, Birgit Jaspers and Lukas Radbruch

| 1. Firstly, we would like to gain an insight into the impact, structures and working practices of your **hospital palliative care support team** due to the COVID-19 pandemic.  Has a COVID-19 area been or will be established at your hospital? /Multiple answers possible) | | | | | | | | | | | | | | | | | | | | | | | | | | | | | | | | | | | | | | | | | | | | | | | | | | | | | | | | | | | | | | | | | | | | | | | | | | |  |
| --- | --- | --- | --- | --- | --- | --- | --- | --- | --- | --- | --- | --- | --- | --- | --- | --- | --- | --- | --- | --- | --- | --- | --- | --- | --- | --- | --- | --- | --- | --- | --- | --- | --- | --- | --- | --- | --- | --- | --- | --- | --- | --- | --- | --- | --- | --- | --- | --- | --- | --- | --- | --- | --- | --- | --- | --- | --- | --- | --- | --- | --- | --- | --- | --- | --- | --- | --- | --- | --- | --- | --- | --- | --- | --- | --- |
|  | Yes, a quarantine ward or area for Covid-19 suspected cases | | | | | | | | | | Yes, an isolation ward or area for positively tested patients | | | | | | | | | | | | | | | | | | | Yes, a general ward with isolation rooms | | | | | | | | | | | | Yes, a general ward with a quarantine area | | | | | | | | | | | | | | | | | | | | | No | | | | | | | | | | | |  |
| 1^st^ Lockdown (Mar – Apr 2020) | □ | | | | | | | | | | □ | | | | | | | | | | | | | | | | | | | □ | | | | | | | | | | | | □ | | | | | | | | | | | | | | | | | | | | | □ | | | | | | | | | | | |  |
| Interim Phase May Oct 2020) | □ | | | | | | | | | | □ | | | | | | | | | | | | | | | | | | | □ | | | | | | | | | | | | □ | | | | | | | | | | | | | | | | | | | | | □ | | | | | | | | | | | |  |
| 2^nd^ Lockdown (Nov 2020 – Feb 2021) | □ | | | | | | | | | | □ | | | | | | | | | | | | | | | | | | | □ | | | | | | | | | | | | □ | | | | | | | | | | | | | | | | | | | | | □ | | | | | | | | | | | |  |
| If **no**, please explain why … | | | | | | | | | | | | | | | | | | | | | | | | | | | | | | | | | | | | | | | | | | | | | | | | | | | | | | | | | | | | | | | | | | | | | | | | | | |  |
|  | | | | | | | | | | | | | | | | | | | | | | | | | | | | | | | | | | | | | | | | | | | | | | | | | | | | | | | | | | | | | | | | | | | | | | | | | | |  |
| 2. Have there been any changes or restructuring of your palliative care service during the COVID-19 pandemic? | | | | | | | | | | | | | | | | | | | | | | | | | | | | | | | | | | | | | | | | | | | | | | | | | | | | | | | | | | | | | | | | | | | | | | | | | | |  |
|  | Yes, our team was/will be expanded | | | | | | | | | | Yes, our team was/will be downsized | | | | | | | | | | | | | | | | | | | Number of staff remains/ remained unchanged | | | | | | | | | | | | Yes, there are/will be the following changes | | | | | | | | | | | | | | | | | | | | | | | | | | | | | | | | |  |
| 1^st^ Lockdown (Mar – Apr 2020) | □ | | | | | | | | | | □ | | | | | | | | | | | | | | | | | | | □ | | | | | | | | | | | | □ | | | | | | | | | | | | | | | | | | | | | | | | | | | | | | | | |  |
| Interim Phase May Oct 2020) | □ | | | | | | | | | | □ | | | | | | | | | | | | | | | | | | | □ | | | | | | | | | | | | □ | | | | | | | | | | | | | | | | | | | | | | | | | | | | | | | | |  |
| 2^nd^ Lockdown (Nov 2020 – Feb 2021) | □ | | | | | | | | | | □ | | | | | | | | | | | | | | | | | | | □ | | | | | | | | | | | | □ | | | | | | | | | | | | | | | | | | | | | | | | | | | | | | | | |  |
| Here you have the possibility to inform us about the changes | | | | | | | | | | | | | | | | | | | | | | | | | | | | | | | | | | | | | | | | | | | | | | | | | | | | | | | | | | | | | | | | | | | | | | | | | | |  |
|  | | | | | | | | | | | | | | | | | | | | | | | | | | | | | | | | | | | | | | | | | | | | | | | | | | | | | | | | | | | | | | | | | | | | | | | | | | |  |
| 3. During the COVID-19 pandemic, were or will staff from your service be recruited or deployed to other units? | | | | | | | | | | | | | | | | | | | | | | | | | | | | | | | | | | | | | | | | | | | | | | | | | | | | | | | | | | | | | | | | | | | | | | | | | | |  |
|  | 1^st^ Lockdown (Mar – Apr 2020) | | | | | | | | | | | | | | | | | | | | | | | Interim Phase May Oct 2020) | | | | | | | | | | | | | | | | | | | | | | | | | | 2^nd^ Lockdown (Nov 2020 – Feb 2021) | | | | | | | | | | | | | | | | | | | | | | | | |  |
| Yes, nurses have been or are being deployed on other wards |  | | | | | | | | | | | | | | | | | | | | | | |  | | | | | | | | | | | | | | | | | | | | | | | | | |  | | | | | | | | | | | | | | | | | | | | | | | | |  |
| Yes, nurses have been or will be deployed in a COVID-19 area (incl. intensive care unit) |  | | | | | | | | | | | | | | | | | | | | | | |  | | | | | | | | | | | | | | | | | | | | | | | | | |  | | | | | | | | | | | | | | | | | | | | | | | | |  |
| Yes, physicians have been or are being deployed on other wards |  | | | | | | | | | | | | | | | | | | | | | | |  | | | | | | | | | | | | | | | | | | | | | | | | | |  | | | | | | | | | | | | | | | | | | | | | | | | |  |
| Yes, physicians have been or will be deployed in a COVID-19 area (incl. intensive care unit) |  | | | | | | | | | | | | | | | | | | | | | | |  | | | | | | | | | | | | | | | | | | | | | | | | | |  | | | | | | | | | | | | | | | | | | | | | | | | |  |
| Yes, other staff have been or are being deployed on other wards |  | | | | | | | | | | | | | | | | | | | | | | |  | | | | | | | | | | | | | | | | | | | | | | | | | |  | | | | | | | | | | | | | | | | | | | | | | | | |  |
| Yes, other staff have been or will be deployed in a COVID-19 area (incl. intensive care unit) |  | | | | | | | | | | | | | | | | | | | | | | |  | | | | | | | | | | | | | | | | | | | | | | | | | |  | | | | | | | | | | | | | | | | | | | | | | | | |  |
| No, no staff has been or is being deployed elsewhere |  | | | | | | | | | | | | | | | | | | | | | | |  | | | | | | | | | | | | | | | | | | | | | | | | | |  | | | | | | | | | | | | | | | | | | | | | | | | |  |
| Is there anything else you would like to tell us about this? | | | | | | | | | | | | | | | | | | | | | | | | | | | | | | | | | | | | | | | | | | | | | | | | | | | | | | | | | | | | | | | | | | | | | | | | | | |  |
|  | | | | | | | | | | | | | | | | | | | | | | | | | | | | | | | | | | | | | | | | | | | | | | | | | | | | | | | | | | | | | | | | | | | | | | | | | | |  |
| 4. Have patient admissions in the hospital as a whole been reduced or stopped due to the COVID-19 pandemic? | | | | | | | | | | | | | | | | | | | | | | | | | | | | | | | | | | | | | | | | | | | | | | | | | | | | | | | | | | | | | | | | | | | | | | | | | | |  |
|  | Yes, reduced | | | | | | | | | | | | | | | | | | | | | Yes, stopped | | | | | | | | | | | | | | | | | | | | | | | | | | No | | | | | | | | | | | | | | | | | | | | | | | | | | |  |
| 1^st^ Lockdown (Mar – Apr 2020) | □ | | | | | | | | | | | | | | | | | | | | | □ | | | | | | | | | | | | | | | | | | | | | | | | | | □ | | | | | | | | | | | | | | | | | | | | | | | | | | |  |
| Interim Phase May Oct 2020) | □ | | | | | | | | | | | | | | | | | | | | | □ | | | | | | | | | | | | | | | | | | | | | | | | | | □ | | | | | | | | | | | | | | | | | | | | | | | | | | |  |
| 2^nd^ Lockdown (Nov 2020 – Feb 2021) | □ | | | | | | | | | | | | | | | | | | | | | □ | | | | | | | | | | | | | | | | | | | | | | | | | | □ | | | | | | | | | | | | | | | | | | | | | | | | | | |  |
| 5. Have patient admissions to your service been reduced or stopped due to the COVID-19 pandemic? | | | | | | | | | | | | | | | | | | | | | | | | | | | | | | | | | | | | | | | | | | | | | | | | | | | | | | | | | | | | | | | | | | | | | | | | | | |  |
|  | Yes, admission have been or are being reduced | | | | | | | | | | | | | | | | | | | | | Yes, there was or is an admission freeze | | | | | | | | | | | | | | | | | | | | | | | | | | No | | | | | | | | | | | | | | | | | | | | | | | | | | |  |
| 1^st^ Lockdown (Mar – Apr 2020) | □ | | | | | | | | | | | | | | | | | | | | | □ | | | | | | | | | | | | | | | | | | | | | | | | | | □ | | | | | | | | | | | | | | | | | | | | | | | | | | |  |
| Interim Phase May Oct 2020) | □ | | | | | | | | | | | | | | | | | | | | | □ | | | | | | | | | | | | | | | | | | | | | | | | | | □ | | | | | | | | | | | | | | | | | | | | | | | | | | |  |
| 2^nd^ Lockdown (Nov 2020 – Feb 2021) | □ | | | | | | | | | | | | | | | | | | | | | □ | | | | | | | | | | | | | | | | | | | | | | | | | | □ | | | | | | | | | | | | | | | | | | | | | | | | | | |  |
| 6. Have there been or are there any changes in the palliative care service on the part of the otherwise requesting wards, as compared to the time before the COVID-19 pandemic?  The requests from the wards that requested your service before the pandemic ... | | | | | | | | | | | | | | | | | | | | | | | | | | | | | | | | | | | | | | | | | | | | | | | | | | | | | | | | | | | | | | | | | | | | | | | | | | |  |
|  | | Decreased considerably | | | | | | | Decreased | | | | | | | | | | | | | | | | Remained unchanged | | | | | | | | | | | | | | Increased | | | | | | | | | | | | | Increased considerably | | | | | | | | | | | | | | | | | | Don’t know | | | | |  |
| 1^st^ Lockdown (Mar – Apr 2020) | |  | | | | | | |  | | | | | | | | | | | | | | | |  | | | | | | | | | | | | | |  | | | | | | | | | | | | |  | | | | | | | | | | | | | | | | | |  | | | | |  |
| Interim Phase May Oct 2020) | |  | | | | | | |  | | | | | | | | | | | | | | | |  | | | | | | | | | | | | | |  | | | | | | | | | | | | |  | | | | | | | | | | | | | | | | | |  | | | | |  |
| 2^nd^ Lockdown (Nov 2020 – Feb 2021) | |  | | | | | | |  | | | | | | | | | | | | | | | |  | | | | | | | | | | | | | |  | | | | | | | | | | | | |  | | | | | | | | | | | | | | | | | |  | | | | |  |
| 7. Have there been or are there any patients at your hospital who have tested positive for SARS-CoV-2 or suspected cases? | | | | | | | | | | | | | | | | | | | | | | | | | | | | | | | | | | | | | | | | | | | | | | | | | | | | | | | | | | | | | | | | | | | | | | | | | | |  |
|  | Yes, persons who tested positive | | | | | | | | | | | | | | | | | | | | | | No, only suspected cases | | | | | | | | | | | | | | | | | | | | | | | | | | | | No, neither | | | | | | | | | | | | | | | | | | | | | | | |  |
| 1^st^ Lockdown (Mar – Apr 2020) | □ | | | | | | | | | | | | | | | | | | | | | | □ | | | | | | | | | | | | | | | | | | | | | | | | | | | | □ | | | | | | | | | | | | | | | | | | | | | | | |  |
| Interim Phase May Oct 2020) | □ | | | | | | | | | | | | | | | | | | | | | | □ | | | | | | | | | | | | | | | | | | | | | | | | | | | | □ | | | | | | | | | | | | | | | | | | | | | | | |  |
| 2^nd^ Lockdown (Nov 2020 – Feb 2021) | □ | | | | | | | | | | | | | | | | | | | | | | □ | | | | | | | | | | | | | | | | | | | | | | | | | | | | □ | | | | | | | | | | | | | | | | | | | | | | | |  |
| 8. How did or do you deal with positive tested patients? | | | | | | | | | | | | | | | | | | | | | | | | | | | | | | | | | | | | | | | | | | | | | | | | | | | | | | | | | | | | | | | | | | | | | | | | | | |  |
|  | They remain(ed) or remain at general wards isolated in a single room | | | | | | | | | | | | | | | They were/are transferred to a single room at the COVID-19 area | | | | | | | | | | | | | | | | | | | | They were/are transferred to a shared room at COVID-19 area | | | | | | | | | | | | | | | | | | | | | | They were/are transferred to another hospital with a COVID-19 area | | | | | | | | | | | | | | | | |  |
| 1^st^ Lockdown (Mar – Apr 2020) | □ | | | | | | | | | | | | | | | □ | | | | | | | | | | | | | | | | | | | | □ | | | | | | | | | | | | | | | | | | | | | | □ | | | | | | | | | | | | | | | | |  |
| Interim Phase May Oct 2020) | □ | | | | | | | | | | | | | | | □ | | | | | | | | | | | | | | | | | | | | □ | | | | | | | | | | | | | | | | | | | | | | □ | | | | | | | | | | | | | | | | |  |
| 2^nd^ Lockdown (Nov 2020 – Feb 2021) | □ | | | | | | | | | | | | | | | □ | | | | | | | | | | | | | | | | | | | | □ | | | | | | | | | | | | | | | | | | | | | | □ | | | | | | | | | | | | | | | | |  |
| 9. Have staff members of your team taken on tasks in a COVID-19 area? If yes, please tick which ones (Multiple answers possible) | | | | | | | | | | | | | | | | | | | | | | | | | | | | | | | | | | | | | | | | | | | | | | | | | | | | | | | | | | | | | | | | | | | | | | | | | | |  |
|  | Symptom management | | | | | Support of relatives | | | | | | | | | | | | | | | | Advice to colleagues | | | | | | | | | | | | | | Discussing goals of care | | | | | | | | | | | | End-of-life care | | | | | | | | | | | | | | | | | No | | | | | | | | | |  |
| 1^st^ Lockdown (Mar – Apr 2020) | □ | | | | | □ | | | | | | | | | | | | | | | | □ | | | | | | | | | | | | | | □ | | | | | | | | | | | | □ | | | | | | | | | | | | | | | | | □ | | | | | | | | | |  |
| Interim Phase May Oct 2020) | □ | | | | | □ | | | | | | | | | | | | | | | | □ | | | | | | | | | | | | | | □ | | | | | | | | | | | | □ | | | | | | | | | | | | | | | | | □ | | | | | | | | | |  |
| 2^nd^ Lockdown (Nov 2020 – Feb 2021) | □ | | | | | □ | | | | | | | | | | | | | | | | □ | | | | | | | | | | | | | | □ | | | | | | | | | | | | □ | | | | | | | | | | | | | | | | | □ | | | | | | | | | |  |
| Is there anything else you would like to tell us about this? | | | | | | | | | | | | | | | | | | | | | | | | | | | | | | | | | | | | | | | | | | | | | | | | | | | | | | | | | | | | | | | | | | | | | | | | | | |  |
|  | | | | | | | | | | | | | | | | | | | | | | | | | | | | | | | | | | | | | | | | | | | | | | | | | | | | | | | | | | | | | | | | | | | | | | | | | | |  |
| 10. How well or poorly does your hospital provide palliative care for patients with severe COVID-19 infection? | | | | | | | | | | | | | | | | | | | | | | | | | | | | | | | | | | | | | | | | | | | | | | | | | | | | | | | | | | | | | | | | | | | | | | | | | | |  |
|  | Very well | | | | | | | | | | Well | | | | | | | | | | | | | | | | | | | Poorly | | | | | | | | | | | | Very poorly | | | | | | | | | | | | | | | | | | | | | Don’t know | | | | | | | | | | | |  |
| 1^st^ Lockdown (Mar – Apr 2020) | □ | | | | | | | | | | □ | | | | | | | | | | | | | | | | | | | □ | | | | | | | | | | | | □ | | | | | | | | | | | | | | | | | | | | | □ | | | | | | | | | | | |  |
| Interim Phase May Oct 2020) | □ | | | | | | | | | | □ | | | | | | | | | | | | | | | | | | | □ | | | | | | | | | | | | □ | | | | | | | | | | | | | | | | | | | | | □ | | | | | | | | | | | |  |
| 2^nd^ Lockdown (Nov 2020 – Feb 2021) | □ | | | | | | | | | | □ | | | | | | | | | | | | | | | | | | | □ | | | | | | | | | | | | □ | | | | | | | | | | | | | | | | | | | | | □ | | | | | | | | | | | |  |
| 11. Were there or are there more or fewer patients for your service compared to the time before the COVID-19 pandemic? | | | | | | | | | | | | | | | | | | | | | | | | | | | | | | | | | | | | | | | | | | | | | | | | | | | | | | | | | | | | | | | | | | | | | | | | | | |  |
|  | Considerably less | | | | | | Less | | | | | | | | | | | | | | | | Unchanged | | | | | | | | | | | | | More | | | | | | | | | | | | | | | Considerably more | | | | | | | | | | | | | | | | | Don’t know | | | | | | |  |
| 1^st^ Lockdown (Mar – Apr 2020) | □ | | | | | | □ | | | | | | | | | | | | | | | | □ | | | | | | | | | | | | | □ | | | | | | | | | | | | | | | □ | | | | | | | | | | | | | | | | | □ | | | | | | |  |
| Interim Phase May Oct 2020) | □ | | | | | | □ | | | | | | | | | | | | | | | | □ | | | | | | | | | | | | | □ | | | | | | | | | | | | | | | □ | | | | | | | | | | | | | | | | | □ | | | | | | |  |
| 2^nd^ Lockdown (Nov 2020 – Feb 2021) | □ | | | | | | □ | | | | | | | | | | | | | | | | □ | | | | | | | | | | | | | □ | | | | | | | | | | | | | | | □ | | | | | | | | | | | | | | | | | □ | | | | | | |  |
| 12. In general, how do you rate the length of stay of patients due to the COVID-19 pandemic, as compared to before? | | | | | | | | | | | | | | | | | | | | | | | | | | | | | | | | | | | | | | | | | | | | | | | | | | | | | | | | | | | | | | | | | | | | | | | | | | |  |
| The length of stay | | | | | | | | | | | | | | | | | | | | | | | | | | | | | | | | | | | | | | | | | | | | | | | | | | | | | | | | | | | | | | | | | | | | | | | | | | |  |
|  | Increased considerably | | | | | | Increased | | | | | | | | | | | | | | | | Remained unchanged | | | | | | | | | | | | | Decreased | | | | | | | | | | | | | | | Decreased considerably | | | | | | | | | | | | | | | | | Don’t know | | | | | | |  |
| 1^st^ Lockdown (Mar – Apr 2020) | □ | | | | | | □ | | | | | | | | | | | | | | | | □ | | | | | | | | | | | | | □ | | | | | | | | | | | | | | | □ | | | | | | | | | | | | | | | | | □ | | | | | | |  |
| Interim Phase May Oct 2020) | □ | | | | | | □ | | | | | | | | | | | | | | | | □ | | | | | | | | | | | | | □ | | | | | | | | | | | | | | | □ | | | | | | | | | | | | | | | | | □ | | | | | | |  |
| 2^nd^ Lockdown (Nov 2020 – Feb 2021) | □ | | | | | | □ | | | | | | | | | | | | | | | | □ | | | | | | | | | | | | | □ | | | | | | | | | | | | | | | □ | | | | | | | | | | | | | | | | | □ | | | | | | |  |
| 13. Were or are patients tested for SARS-CoV-2 before or after admission? | | | | | | | | | | | | | | | | | | | | | | | | | | | | | | | | | | | | | | | | | | | | | | | | | | | | | | | | | | | | | | | | | | | | | | | | | | |  |
|  | All before admission | | | | | | | | | | | In case of symptoms before admission | | | | | | | | | | | | | | | | | | | All after admission | | | | | | | | | | | | In case of symptoms after admission | | | | | | | | | | | | | | | | | | | | | No testing | | | | | | | | | | |  |
| 1^st^ Lockdown (Mar – Apr 2020) | □ | | | | | | | | | | | □ | | | | | | | | | | | | | | | | | | | □ | | | | | | | | | | | | □ | | | | | | | | | | | | | | | | | | | | | □ | | | | | | | | | | |  |
| Interim Phase May Oct 2020) | □ | | | | | | | | | | | □ | | | | | | | | | | | | | | | | | | | □ | | | | | | | | | | | | □ | | | | | | | | | | | | | | | | | | | | | □ | | | | | | | | | | |  |
| 2^nd^ Lockdown (Nov 2020 – Feb 2021) | □ | | | | | | | | | | | □ | | | | | | | | | | | | | | | | | | | □ | | | | | | | | | | | | □ | | | | | | | | | | | | | | | | | | | | | □ | | | | | | | | | | |  |
| 14. What testing procedures are used with patients? | | | | | | | | | | | | | | | | | | | | | | | | | | | | | | | | | | | | | | | | | | | | | | | | | | | | | | | | | | | | | | | | | | | | | | | | | | |  |
|  | PCR tests financed by the hospital | | | | | | | | | | | Tests financed by the patients themselves | | | | | | | | | | | | | | | | | | | PoCT financed by the hospital | | | | | | | | | | | | PoCT financed by the patients themselves | | | | | | | | | | | | | | | | | | | | | Other | | | | | | | | | | |  |
| 1^st^ Lockdown (Mar – Apr 2020) | □ | | | | | | | | | | | □ | | | | | | | | | | | | | | | | | | | □ | | | | | | | | | | | | □ | | | | | | | | | | | | | | | | | | | | | □ | | | | | | | | | | |  |
| Interim Phase May Oct 2020) | □ | | | | | | | | | | | □ | | | | | | | | | | | | | | | | | | | □ | | | | | | | | | | | | □ | | | | | | | | | | | | | | | | | | | | | □ | | | | | | | | | | |  |
| 2^nd^ Lockdown (Nov 2020 – Feb 2021) | □ | | | | | | | | | | | □ | | | | | | | | | | | | | | | | | | | □ | | | | | | | | | | | | □ | | | | | | | | | | | | | | | | | | | | | □ | | | | | | | | | | |  |
| 15. Have patients been or will they be tested again or regularly during their stay? | | | | | | | | | | | | | | | | | | | | | | | | | | | | | | | | | | | | | | | | | | | | | | | | | | | | | | | | | | | | | | | | | | | | | | | | | | |  |
|  | All patients on a regular basis during their stay | | | | | | | | | | | | | | | | | | | | | | Patients with symptoms on a regular basis during the stay | | | | | | | | | | | | | | | | | | | | | | | | | | | | No testing | | | | | | | | | | | | | | | | | | | | | | | |  |
| 1^st^ Lockdown (Mar – Apr 2020) | □ | | | | | | | | | | | | | | | | | | | | | | □ | | | | | | | | | | | | | | | | | | | | | | | | | | | | □ | | | | | | | | | | | | | | | | | | | | | | | |  |
| Interim Phase May Oct 2020) | □ | | | | | | | | | | | | | | | | | | | | | | □ | | | | | | | | | | | | | | | | | | | | | | | | | | | | □ | | | | | | | | | | | | | | | | | | | | | | | |  |
| 2^nd^ Lockdown (Nov 2020 – Feb 2021) | □ | | | | | | | | | | | | | | | | | | | | | | □ | | | | | | | | | | | | | | | | | | | | | | | | | | | | □ | | | | | | | | | | | | | | | | | | | | | | | |  |
| 16. Have patients been or will they be tested before internal transfers? | | | | | | | | | | | | | | | | | | | | | | | | | | | | | | | | | | | | | | | | | | | | | | | | | | | | | | | | | | | | | | | | | | | | | | | | | | |  |
|  | All patients before internal transfers | | | | | | | | | | | | | | | | | | | | | | Patients with symptoms before internal transfers | | | | | | | | | | | | | | | | | | | | | | | | | | No testing | | | | | | | | | | | | | | | | | | | | | | | | | |  |
| 1^st^ Lockdown (Mar – Apr 2020) | □ | | | | | | | | | | | | | | | | | | | | | | □ | | | | | | | | | | | | | | | | | | | | | | | | | | □ | | | | | | | | | | | | | | | | | | | | | | | | | |  |
| Interim Phase May Oct 2020) | □ | | | | | | | | | | | | | | | | | | | | | | □ | | | | | | | | | | | | | | | | | | | | | | | | | | □ | | | | | | | | | | | | | | | | | | | | | | | | | |  |
| 2^nd^ Lockdown (Nov 2020 – Feb 2021) | □ | | | | | | | | | | | | | | | | | | | | | | □ | | | | | | | | | | | | | | | | | | | | | | | | | | □ | | | | | | | | | | | | | | | | | | | | | | | | | |  |
| 17. Have patients been or will they be tested before being discharged home with involvement of home care services? | | | | | | | | | | | | | | | | | | | | | | | | | | | | | | | | | | | | | | | | | | | | | | | | | | | | | | | | | | | | | | | | | | | | | | | | | | |  |
|  | All patients | | | | | | | | | | | | | | | | | | | | | | Patients with symptoms | | | | | | | | | | | | | | | | | | | | | | | | | | No testing | | | | | | | | | | | | | | | | | | | | | | | | | |  |
| 1^st^ Lockdown (Mar – Apr 2020) | □ | | | | | | | | | | | | | | | | | | | | | | □ | | | | | | | | | | | | | | | | | | | | | | | | | | □ | | | | | | | | | | | | | | | | | | | | | | | | | |  |
| Interim Phase May Oct 2020) | □ | | | | | | | | | | | | | | | | | | | | | | □ | | | | | | | | | | | | | | | | | | | | | | | | | | □ | | | | | | | | | | | | | | | | | | | | | | | | | |  |
| 2^nd^ Lockdown (Nov 2020 – Feb 2021) | □ | | | | | | | | | | | | | | | | | | | | | | □ | | | | | | | | | | | | | | | | | | | | | | | | | | □ | | | | | | | | | | | | | | | | | | | | | | | | | |  |
| 18. Have patients been or will they be tested before being discharged home without involvement of home care services? | | | | | | | | | | | | | | | | | | | | | | | | | | | | | | | | | | | | | | | | | | | | | | | | | | | | | | | | | | | | | | | | | | | | | | | | | | |  |
|  | All patients | | | | | | | | | | | | | | | | | | | | | | Patients with symptoms | | | | | | | | | | | | | | | | | | | | | | | | | | No testing | | | | | | | | | | | | | | | | | | | | | | | | | |  |
| 1^st^ Lockdown (Mar – Apr 2020) | □ | | | | | | | | | | | | | | | | | | | | | | □ | | | | | | | | | | | | | | | | | | | | | | | | | | □ | | | | | | | | | | | | | | | | | | | | | | | | | |  |
| Interim Phase May Oct 2020) | □ | | | | | | | | | | | | | | | | | | | | | | □ | | | | | | | | | | | | | | | | | | | | | | | | | | □ | | | | | | | | | | | | | | | | | | | | | | | | | |  |
| 2^nd^ Lockdown (Nov 2020 – Feb 2021) | □ | | | | | | | | | | | | | | | | | | | | | | □ | | | | | | | | | | | | | | | | | | | | | | | | | | □ | | | | | | | | | | | | | | | | | | | | | | | | | |  |
| 19. Have patients been or will they be tested before being discharged to an inpatient hospice? | | | | | | | | | | | | | | | | | | | | | | | | | | | | | | | | | | | | | | | | | | | | | | | | | | | | | | | | | | | | | | | | | | | | | | | | | | |  |
|  | All patients | | | | | | | | | | | | | | | | | | | | | | Patients with symptoms | | | | | | | | | | | | | | | | | | | | | | | | | | No testing | | | | | | | | | | | | | | | | | | | | | | | | | |  |
| 1^st^ Lockdown (Mar – Apr 2020) | □ | | | | | | | | | | | | | | | | | | | | | | □ | | | | | | | | | | | | | | | | | | | | | | | | | | □ | | | | | | | | | | | | | | | | | | | | | | | | | |  |
| Interim Phase May Oct 2020) | □ | | | | | | | | | | | | | | | | | | | | | | □ | | | | | | | | | | | | | | | | | | | | | | | | | | □ | | | | | | | | | | | | | | | | | | | | | | | | | |  |
| 2^nd^ Lockdown (Nov 2020 – Feb 2021) | □ | | | | | | | | | | | | | | | | | | | | | | □ | | | | | | | | | | | | | | | | | | | | | | | | | | □ | | | | | | | | | | | | | | | | | | | | | | | | | |  |
| 20. Have patients been or will they be tested before being discharged to a care home/nursing home? | | | | | | | | | | | | | | | | | | | | | | | | | | | | | | | | | | | | | | | | | | | | | | | | | | | | | | | | | | | | | | | | | | | | | | | | | | |  |
|  | All patients | | | | | | | | | | | | | | | | | | | | | | Patients with symptoms | | | | | | | | | | | | | | | | | | | | | | | | | | No testing | | | | | | | | | | | | | | | | | | | | | | | | | |  |
| 1^st^ Lockdown (Mar – Apr 2020) | □ | | | | | | | | | | | | | | | | | | | | | | □ | | | | | | | | | | | | | | | | | | | | | | | | | | □ | | | | | | | | | | | | | | | | | | | | | | | | | |  |
| Interim Phase May Oct 2020) | □ | | | | | | | | | | | | | | | | | | | | | | □ | | | | | | | | | | | | | | | | | | | | | | | | | | □ | | | | | | | | | | | | | | | | | | | | | | | | | |  |
| 2^nd^ Lockdown (Nov 2020 – Feb 2021) | □ | | | | | | | | | | | | | | | | | | | | | | □ | | | | | | | | | | | | | | | | | | | | | | | | | | □ | | | | | | | | | | | | | | | | | | | | | | | | | |  |
| 21. How difficult or easy is it to get a test result in time before dismissals? | | | | | | | | | | | | | | | | | | | | | | | | | | | | | | | | | | | | | | | | | | | | | | | | | | | | | | | | | | | | | | | | | | | | | | | | | | |  |
|  | Very difficult | | | | | | | | | | Difficult | | | | | | | | | | | | | | | | | | | Easy | | | | | | | | | | | | Very easy | | | | | | | | | | | | | | | | | | | | | Don’t know | | | | | | | | | | | |  |
| 1^st^ Lockdown (Mar – Apr 2020) | □ | | | | | | | | | | □ | | | | | | | | | | | | | | | | | | | □ | | | | | | | | | | | | □ | | | | | | | | | | | | | | | | | | | | | □ | | | | | | | | | | | |  |
| Interim Phase May Oct 2020) | □ | | | | | | | | | | □ | | | | | | | | | | | | | | | | | | | □ | | | | | | | | | | | | □ | | | | | | | | | | | | | | | | | | | | | □ | | | | | | | | | | | |  |
| 2^nd^ Lockdown (Nov 2020 – Feb 2021) | □ | | | | | | | | | | □ | | | | | | | | | | | | | | | | | | | □ | | | | | | | | | | | | □ | | | | | | | | | | | | | | | | | | | | | □ | | | | | | | | | | | |  |
| Next, we would like to ask you two questions about the testing of visitors/family.  22. Have visitors/family been or are being tested? | | | | | | | | | | | | | | | | | | | | | | | | | | | | | | | | | | | | | | | | | | | | | | | | | | | | | | | | | | | | | | | | | | | | | | | | | | |  |
|  | We tested/test all persons on site | | | | | | | | | | | All persons had/had to show a negative test result | | | | | | | | | | | | | | | | | | | We tested persons with symptoms on site | | | | | | | | | | | | Persons with symptoms had/had to show a negative test result | | | | | | | | | | | | | | | | | | | | | No test certificate required | | | | | | | | | | |  |
| 1^st^ Lockdown (Mar – Apr 2020) | □ | | | | | | | | | | | □ | | | | | | | | | | | | | | | | | | | □ | | | | | | | | | | | | □ | | | | | | | | | | | | | | | | | | | | | □ | | | | | | | | | | |  |
| Interim Phase May Oct 2020) | □ | | | | | | | | | | | □ | | | | | | | | | | | | | | | | | | | □ | | | | | | | | | | | | □ | | | | | | | | | | | | | | | | | | | | | □ | | | | | | | | | | |  |
| 2^nd^ Lockdown (Nov 2020 – Feb 2021) | □ | | | | | | | | | | | □ | | | | | | | | | | | | | | | | | | | □ | | | | | | | | | | | | □ | | | | | | | | | | | | | | | | | | | | | □ | | | | | | | | | | |  |
| 23. What testing procedures are used with visitors/family? | | | | | | | | | | | | | | | | | | | | | | | | | | | | | | | | | | | | | | | | | | | | | | | | | | | | | | | | | | | | | | | | | | | | | | | | | | |  |
|  | PCR tests financed by the hospital | | | | | | | | | | | PCR tests financed by persons themselves | | | | | | | | | | | | | | | | | | | PoCT financed by the hospital | | | | | | | | | | | | PoCT financed by persons themselves | | | | | | | | | | | | | | | | | | | | | Other | | | | | | | | | | |  |
| 1^st^ Lockdown (Mar – Apr 2020) | □ | | | | | | | | | | | □ | | | | | | | | | | | | | | | | | | | □ | | | | | | | | | | | | □ | | | | | | | | | | | | | | | | | | | | | □ | | | | | | | | | | |  |
| Interim Phase May Oct 2020) | □ | | | | | | | | | | | □ | | | | | | | | | | | | | | | | | | | □ | | | | | | | | | | | | □ | | | | | | | | | | | | | | | | | | | | | □ | | | | | | | | | | |  |
| 2^nd^ Lockdown (Nov 2020 – Feb 2021) | □ | | | | | | | | | | | □ | | | | | | | | | | | | | | | | | | | □ | | | | | | | | | | | | □ | | | | | | | | | | | | | | | | | | | | | □ | | | | | | | | | | |  |
| **Staff testing**  And now we would like to get some insight into the testing of staff.  24. Have all staff of your service been or will be tested? | | | | | | | | | | | | | | | | | | | | | | | | | | | | | | | | | | | | | | | | | | | | | | | | | | | | | | | | | | | | | | | | | | | | | | | | | | |  |
|  | Yes, all | | | | | | | | | | | | | | | | Only when showing symptoms | | | | | | | | | | | | | | | | | | | At their request | | | | | | | | | | | | | | | | | | | | | | | | No testing | | | | | | | | | | | | | | |  |
| 1^st^ Lockdown (Mar – Apr 2020) | □ | | | | | | | | | | | | | | | | □ | | | | | | | | | | | | | | | | | | | □ | | | | | | | | | | | | | | | | | | | | | | | | □ | | | | | | | | | | | | | | |  |
| Interim Phase May Oct 2020) | □ | | | | | | | | | | | | | | | | □ | | | | | | | | | | | | | | | | | | | □ | | | | | | | | | | | | | | | | | | | | | | | | □ | | | | | | | | | | | | | | |  |
| 2^nd^ Lockdown (Nov 2020 – Feb 2021) | □ | | | | | | | | | | | | | | | | □ | | | | | | | | | | | | | | | | | | | □ | | | | | | | | | | | | | | | | | | | | | | | | □ | | | | | | | | | | | | | | |  |
| 25. Do these tests take place on a regular basis? | | | | | | | | | | | | | | | | | | | | | | | | | | | | | | | | | | | | | | | | | | | | | | | | | | | | | | | | | | | | | | | | | | | | | | | | | | |  |
|  | Yes | | | | | | | | | | | | | | | | | | | | | | No | | | | | | | | | | | | | | | | | | | | | | | | | | | | N./a. | | | | | | | | | | | | | | | | | | | | | | | |  |
| 1^st^ Lockdown (Mar – Apr 2020) | □ | | | | | | | | | | | | | | | | | | | | | | □ | | | | | | | | | | | | | | | | | | | | | | | | | | | | □ | | | | | | | | | | | | | | | | | | | | | | | |  |
| Interim Phase May Oct 2020) | □ | | | | | | | | | | | | | | | | | | | | | | □ | | | | | | | | | | | | | | | | | | | | | | | | | | | | □ | | | | | | | | | | | | | | | | | | | | | | | |  |
| 2^nd^ Lockdown (Nov 2020 – Feb 2021) | □ | | | | | | | | | | | | | | | | | | | | | | □ | | | | | | | | | | | | | | | | | | | | | | | | | | | | □ | | | | | | | | | | | | | | | | | | | | | | | |  |
| 26. What testing procedures are used with staff? | | | | | | | | | | | | | | | | | | | | | | | | | | | | | | | | | | | | | | | | | | | | | | | | | | | | | | | | | | | | | | | | | | | | | | | | | | |  |
|  | PCR tests financed by the hospital | | | | | | | | | | | PCR tests financed by staff themselves | | | | | | | | | | | | | | | | | | | PoCT financed by the hospital | | | | | | | | | | | | PoCT financed by by staff themselves | | | | | | | | | | | | | | | | | | | | | Other | | | | | | | | | | |  |
| 1^st^ Lockdown (Mar – Apr 2020) | □ | | | | | | | | | | | □ | | | | | | | | | | | | | | | | | | | □ | | | | | | | | | | | | □ | | | | | | | | | | | | | | | | | | | | | □ | | | | | | | | | | |  |
| Interim Phase May Oct 2020) | □ | | | | | | | | | | | □ | | | | | | | | | | | | | | | | | | | □ | | | | | | | | | | | | □ | | | | | | | | | | | | | | | | | | | | | □ | | | | | | | | | | |  |
| 2^nd^ Lockdown (Nov 2020 – Feb 2021) | □ | | | | | | | | | | | □ | | | | | | | | | | | | | | | | | | | □ | | | | | | | | | | | | □ | | | | | | | | | | | | | | | | | | | | | □ | | | | | | | | | | |  |
| 27. What happens to employees who are waiting for their test results? | | | | | | | | | | | | | | | | | | | | | | | | | | | | | | | | | | | | | | | | | | | | | | | | | | | | | | | | | | | | | | | | | | | | | | | | | | |  |
|  | Quarantine at home | | | | | | | | | | | | | | | | | | | | | | Work at the PCU and quarantine at home outside working hours | | | | | | | | | | | | | | | | | | | | | | | | | | | | Work without contact with patients | | | | | | | | | | | | | | | | | | | | | | | |  |
| 1^st^ Lockdown (Mar – Apr 2020) | □ | | | | | | | | | | | | | | | | | | | | | | □ | | | | | | | | | | | | | | | | | | | | | | | | | | | | □ | | | | | | | | | | | | | | | | | | | | | | | |  |
| Interim Phase May Oct 2020) | □ | | | | | | | | | | | | | | | | | | | | | | □ | | | | | | | | | | | | | | | | | | | | | | | | | | | | □ | | | | | | | | | | | | | | | | | | | | | | | |  |
| 2^nd^ Lockdown (Nov 2020 – Feb 2021) | □ | | | | | | | | | | | | | | | | | | | | | | □ | | | | | | | | | | | | | | | | | | | | | | | | | | | | □ | | | | | | | | | | | | | | | | | | | | | | | |  |
| Would you like to comment on the testing or quarantine procedures (e. g. working quarantine, commuter quarantine, waiting times until test results, procedure in case of cold symptoms, etc.)? | | | | | | | | | | | | | | | | | | | | | | | | | | | | | | | | | | | | | | | | | | | | | | | | | | | | | | | | | | | | | | | | | | | | | | | | | | |  |
|  | | | | | | | | | | | | | | | | | | | | | | | | | | | | | | | | | | | | | | | | | | | | | | | | | | | | | | | | | | | | | | | | | | | | | | | | | | |  |
| 28. Have there been or are there any employees in the team who tested positive? | | | | | | | | | | | | | | | | | | | | | | | | | | | | | | | | | | | | | | | | | | | | | | | | | | | | | | | | | | | | | | | | | | | | | | | | | | |  |
|  | Yes | | | | | | | | | | | | | | | | | | | | | | | | | | | | | | | | | | | No | | | | | | | | | | | | | | | | | | | | | | | | | | | | | | | | | | | | | | |  |
| 1^st^ Lockdown (Mar – Apr 2020) | □ | | | | | | | | | | | | | | | | | | | | | | | | | | | | | | | | | | | □ | | | | | | | | | | | | | | | | | | | | | | | | | | | | | | | | | | | | | | |  |
| Interim Phase May Oct 2020) | □ | | | | | | | | | | | | | | | | | | | | | | | | | | | | | | | | | | | □ | | | | | | | | | | | | | | | | | | | | | | | | | | | | | | | | | | | | | | |  |
| 2^nd^ Lockdown (Nov 2020 – Feb 2021) | □ | | | | | | | | | | | | | | | | | | | | | | | | | | | | | | | | | | | □ | | | | | | | | | | | | | | | | | | | | | | | | | | | | | | | | | | | | | | |  |
| 29. Approximately what was the percentage of employees affected? | | | | | | | | | | | | | | | | | | | | | | | | | | | | | | | | | | | | | | | | | | | | | | | | | | | | | | | | | | | | | | | | | | | | | | | | | | |  |
|  | | | | | | | | | | | | | | | | | | | | | | | | | | | | | | | | | | | | | | | | | | | | | | | | | | | | | | | | | | | | | | | | | | | | | | | | | | |  |
| 30. Which of the following services were or will continue to be offered at the PCU during the pandemic? | | | | | | | | | | | | | | | | | | | | | | | | | | | | | | | | | | | | | | | | | | | | | | | | | | | | | | | | | | | | | | | | | | | | | | | | | | |  |
|  | 1^st^ Lockdown (Mar – Apr 2020) | | | | | | | | | | | | | | | | Interim Phase May Oct 2020) | | | | | | | | | | | | | | | | | | | 2^nd^ Lockdown (Nov 2020 – Feb 2021) | | | | | | | | | | | | | | | | | | | | | | | | We do not offer this service | | | | | | | | | | | | | | |  |
| Psychologist/Psycho-oncologist | □ | | | | | | | | | | | | | | | | □ | | | | | | | | | | | | | | | | | | | □ | | | | | | | | | | | | | | | | | | | | | | | | □ | | | | | | | | | | | | | | |  |
| Social Worker | □ | | | | | | | | | | | | | | | | □ | | | | | | | | | | | | | | | | | | | □ | | | | | | | | | | | | | | | | | | | | | | | | □ | | | | | | | | | | | | | | |  |
| Logotherapy, occupational therapy or physiotherapy | □ | | | | | | | | | | | | | | | | □ | | | | | | | | | | | | | | | | | | | □ | | | | | | | | | | | | | | | | | | | | | | | | □ | | | | | | | | | | | | | | |  |
| Art therapy, music therapy, humour therapy, use of singing bowls, animal therapy | □ | | | | | | | | | | | | | | | | □ | | | | | | | | | | | | | | | | | | | □ | | | | | | | | | | | | | | | | | | | | | | | | □ | | | | | | | | | | | | | | |  |
| Pastoral care | □ | | | | | | | | | | | | | | | | □ | | | | | | | | | | | | | | | | | | | □ | | | | | | | | | | | | | | | | | | | | | | | | □ | | | | | | | | | | | | | | |  |
| Bereavement support | □ | | | | | | | | | | | | | | | | □ | | | | | | | | | | | | | | | | | | | □ | | | | | | | | | | | | | | | | | | | | | | | | □ | | | | | | | | | | | | | | |  |
| Volunteers | □ | | | | | | | | | | | | | | | | □ | | | | | | | | | | | | | | | | | | | □ | | | | | | | | | | | | | | | | | | | | | | | | □ | | | | | | | | | | | | | | |  |
| Spiritual care | □ | | | | | | | | | | | | | | | | □ | | | | | | | | | | | | | | | | | | | □ | | | | | | | | | | | | | | | | | | | | | | | | □ | | | | | | | | | | | | | | |  |
| Special offers and programmes, such as ‘Families with Young Children’, ‘Children with parents with cancer’, etc. | □ | | | | | | | | | | | | | | | | □ | | | | | | | | | | | | | | | | | | | □ | | | | | | | | | | | | | | | | | | | | | | | | □ | | | | | | | | | | | | | | |  |
| Other | □ | | | | | | | | | | | | | | | | □ | | | | | | | | | | | | | | | | | | | □ | | | | | | | | | | | | | | | | | | | | | | | | □ | | | | | | | | | | | | | | |  |
| 31. Would you like to add something? | | | | | | | | | | | | | | | | | | | | | | | | | | | | | | | | | | | | | | | | | | | | | | | | | | | | | | | | | | | | | | | | | | | | | | | | | | |  |
|  | | | | | | | | | | | | | | | | | | | | | | | | | | | | | | | | | | | | | | | | | | | | | | | | | | | | | | | | | | | | | | | | | | | | | | | | | | |  |
| If special offers and services (e. g. art therapy, music therapy, bereavement support, volunteers) have been cancelled: What solutions are you thinking about to be able to include them again (e. g. PoCT; having services outdoors on the terrace etc.)? | | | | | | | | | | | | | | | | | | | | | | | | | | | | | | | | | | | | | | | | | | | | | | | | | | | | | | | | | | | | | | | | | | | | | | | | | | |  |
|  | | | | | | | | | | | | | | | | | | | | | | | | | | | | | | | | | | | | | | | | | | | | | | | | | | | | | | | | | | | | | | | | | | | | | | | | | | |  |
| 32. Have alternatives been or are alternatives being created by your facility for services that are not available (e. g. telephone counselling, picture documentation, diaries, video or messenger services - e. g. Skype, FaceTime, WhatsApp, Threema, Signal etc.)? | | | | | | | | | | | | | | | | | | | | | | | | | | | | | | | | | | | | | | | | | | | | | | | | | | | | | | | | | | | | | | | | | | | | | | | | | | |  |
|  | Yes, they are, but they are hardly or not used at all | | | | | | | | | | | Yes, they are used by some or many | | | | | | | | | | | | | | | | | | | Yes, but the Data Protection Regulation does not allow it | | | | | | | | | | | | Yes, but the hygiene concept does not allow it (disinfection of devices etc.) | | | | | | | | | | | | | | | | | | | | | No, we did not come up with alternative offers | | | | | | | | | | |  |
| 1^st^ Lockdown (Mar – Apr 2020) | □ | | | | | | | | | | | □ | | | | | | | | | | | | | | | | | | | □ | | | | | | | | | | | | □ | | | | | | | | | | | | | | | | | | | | | □ | | | | | | | | | | |  |
| Interim Phase May Oct 2020) | □ | | | | | | | | | | | □ | | | | | | | | | | | | | | | | | | | □ | | | | | | | | | | | | □ | | | | | | | | | | | | | | | | | | | | | □ | | | | | | | | | | |  |
| 2^nd^ Lockdown (Nov 2020 – Feb 2021) | □ | | | | | | | | | | | □ | | | | | | | | | | | | | | | | | | | □ | | | | | | | | | | | | □ | | | | | | | | | | | | | | | | | | | | | □ | | | | | | | | | | |  |
| Which of the newly created alternative services would you like to continue to use or use after the pandemic? | | | | | | | | | | | | | | | | | | | | | | | | | | | | | | | | | | | | | | | | | | | | | | | | | | | | | | | | | | | | | | | | | | | | | | | | | | |  |
|  | | | | | | | | | | | | | | | | | | | | | | | | | | | | | | | | | | | | | | | | | | | | | | | | | | | | | | | | | | | | | | | | | | | | | | | | | | |  |
| 33. In your opinion, was there a different need for spiritual care during the pandemic, as compared to before? | | | | | | | | | | | | | | | | | | | | | | | | | | | | | | | | | | | | | | | | | | | | | | | | | | | | | | | | | | | | | | | | | | | | | | | | | | |  |
| The need for spiritual care | | | | | | | | | | | | | | | | | | | | | | | | | | | | | | | | | | | | | | | | | | | | | | | | | | | | | | | | | | | | | | | | | | | | | | | | | | |  |
|  | Increased considerably | | | | | | Increased | | | | | | | | | | | | | | | | Remained unchanged | | | | | | | | | | | | | Decreased | | | | | | | | | | | | | | | Decreased considerably | | | | | | | | | | | | | | | | | N./a. | | | | | | |  |
| 1^st^ Lockdown (Mar – Apr 2020) | □ | | | | | | □ | | | | | | | | | | | | | | | | □ | | | | | | | | | | | | | □ | | | | | | | | | | | | | | | □ | | | | | | | | | | | | | | | | | □ | | | | | | |  |
| Interim Phase May Oct 2020) | □ | | | | | | □ | | | | | | | | | | | | | | | | □ | | | | | | | | | | | | | □ | | | | | | | | | | | | | | | □ | | | | | | | | | | | | | | | | | □ | | | | | | |  |
| 2^nd^ Lockdown (Nov 2020 – Feb 2021) | □ | | | | | | □ | | | | | | | | | | | | | | | | □ | | | | | | | | | | | | | □ | | | | | | | | | | | | | | | □ | | | | | | | | | | | | | | | | | □ | | | | | | |  |
| 34. If you did not provide pastoral care, spiritual care and/or psychological care or if it had to take place in other formats, did this place a burden on the staff (e. g. doctors, nursing staff, social workers) on site? | | | | | | | | | | | | | | | | | | | | | | | | | | | | | | | | | | | | | | | | | | | | | | | | | | | | | | | | | | | | | | | | | | | | | | | | | | |  |
|  | Yes | | | | | | | | | | | | | | | | Partly | | | | | | | | | | | | | | | | | | | No | | | | | | | | | | | | | | | | | | | | | | | | N./a. | | | | | | | | | | | | | | |  |
| 1^st^ Lockdown (Mar – Apr 2020) | □ | | | | | | | | | | | | | | | | □ | | | | | | | | | | | | | | | | | | | □ | | | | | | | | | | | | | | | | | | | | | | | | □ | | | | | | | | | | | | | | |  |
| Interim Phase May Oct 2020) | □ | | | | | | | | | | | | | | | | □ | | | | | | | | | | | | | | | | | | | □ | | | | | | | | | | | | | | | | | | | | | | | | □ | | | | | | | | | | | | | | |  |
| 2^nd^ Lockdown (Nov 2020 – Feb 2021) | □ | | | | | | | | | | | | | | | | □ | | | | | | | | | | | | | | | | | | | □ | | | | | | | | | | | | | | | | | | | | | | | | □ | | | | | | | | | | | | | | |  |
| 35. Was there or is there a complete ban or restriction on visiting the hospital at the times mentioned below? | | | | | | | | | | | | | | | | | | | | | | | | | | | | | | | | | | | | | | | | | | | | | | | | | | | | | | | | | | | | | | | | | | | | | | | | | | |  |
|  | Yes, complete ban | | | | | | | | | | | | | | | | | | | | | | Yes, restrictions | | | | | | | | | | | | | | | | | | | | | | | | | | | | No | | | | | | | | | | | | | | | | | | | | | | | |  |
| 1^st^ Lockdown (Mar – Apr 2020) | □ | | | | | | | | | | | | | | | | | | | | | | □ | | | | | | | | | | | | | | | | | | | | | | | | | | | | □ | | | | | | | | | | | | | | | | | | | | | | | |  |
| Interim Phase May Oct 2020) | □ | | | | | | | | | | | | | | | | | | | | | | □ | | | | | | | | | | | | | | | | | | | | | | | | | | | | □ | | | | | | | | | | | | | | | | | | | | | | | |  |
| 2^nd^ Lockdown (Nov 2020 – Feb 2021) | □ | | | | | | | | | | | | | | | | | | | | | | □ | | | | | | | | | | | | | | | | | | | | | | | | | | | | □ | | | | | | | | | | | | | | | | | | | | | | | |  |
| 36. Were there or are there special visiting regulations or restrictions for severely ill or dying patients? | | | | | | | | | | | | | | | | | | | | | | | | | | | | | | | | | | | | | | | | | | | | | | | | | | | | | | | | | | | | | | | | | | | | | | | | | | |  |
|  | Yes | | | | | | | | | | | | | | | | | | | | | | | | | | | | | | | | | | | | No | | | | | | | | | | | | | | | | | | | | | | | | | | | | | | | | | | | | | |  |
| 1^st^ Lockdown (Mar – Apr 2020) | □ | | | | | | | | | | | | | | | | | | | | | | | | | | | | | | | | | | | | □ | | | | | | | | | | | | | | | | | | | | | | | | | | | | | | | | | | | | | |  |
| Interim Phase May Oct 2020) | □ | | | | | | | | | | | | | | | | | | | | | | | | | | | | | | | | | | | | □ | | | | | | | | | | | | | | | | | | | | | | | | | | | | | | | | | | | | | |  |
| 2^nd^ Lockdown (Nov 2020 – Feb 2021) | □ | | | | | | | | | | | | | | | | | | | | | | | | | | | | | | | | | | | | □ | | | | | | | | | | | | | | | | | | | | | | | | | | | | | | | | | | | | | |  |
| 37. What did the visiting restrictions at your hospital include? | | | | | | | | | | | | | | | | | | | | | | | | | | | | | | | | | | | | | | | | | | | | | | | | | | | | | | | | | | | | | | | | | | | | | | | | | | |  |
|  | Maximum number of persons/day | | | | | | | | | | | | | | | | | | | | | | Maximum number of hours/day | | | | | | | | | | | | | | | | | | | | | | | | | | | | At fixed hours | | | | | | | | | | | | | | | | | | | | | | | |  |
| 1^st^ Lockdown (Mar – Apr 2020) | □ | | | | | | | | | | | | | | | | | | | | | | □ | | | | | | | | | | | | | | | | | | | | | | | | | | | | □ | | | | | | | | | | | | | | | | | | | | | | | |  |
| Interim Phase May Oct 2020) | □ | | | | | | | | | | | | | | | | | | | | | | □ | | | | | | | | | | | | | | | | | | | | | | | | | | | | □ | | | | | | | | | | | | | | | | | | | | | | | |  |
| 2^nd^ Lockdown (Nov 2020 – Feb 2021) | □ | | | | | | | | | | | | | | | | | | | | | | □ | | | | | | | | | | | | | | | | | | | | | | | | | | | | □ | | | | | | | | | | | | | | | | | | | | | | | |  |
| 38. How many relatives were or are allowed to visit the patients at the wards per day and for how long? Can you please briefly describe your visiting arrangements or restrictions in times of the pandemic (e. g. 1 person/day; any number of persons per day, but only x persons at a time)? | | | | | | | | | | | | | | | | | | | | | | | | | | | | | | | | | | | | | | | | | | | | | | | | | | | | | | | | | | | | | | | | | | | | | | | | | | |  |
|  | | | | | | | | | | | | | | | | | | | | | | | | | | | | | | | | | | | | | | | | | | | | | | | | | | | | | | | | | | | | | | | | | | | | | | | | | | |  |
| 39. Were there or are there special or extended visiting arrangements for dying patients (e. g. overnight stays by relatives, more visitors, etc.)? What were or are the regulations for COVID-19 positive relatives of the dying (e. g. strict ban on visits for COVID-19 positive relatives)?  If so, could you please describe them briefly? | | | | | | | | | | | | | | | | | | | | | | | | | | | | | | | | | | | | | | | | | | | | | | | | | | | | | | | | | | | | | | | | | | | | | | | | | | |  |
|  | | | | | | | | | | | | | | | | | | | | | | | | | | | | | | | | | | | | | | | | | | | | | | | | | | | | | | | | | | | | | | | | | | | | | | | | | | |  |
| 40. Were special regulations for visitors/relatives of seriously ill and dying patients implemented at all wards? | | | | | | | | | | | | | | | | | | | | | | | | | | | | | | | | | | | | | | | | | | | | | | | | | | | | | | | | | | | | | | | | | | | | | | | | | | |  |
|  | | | | Yes | | | | | | | | | | | | | | | | | | | | | | | | | No | | | | | | | | | | | | | | | | | | | | | | | | | | Don’t know | | | | | | | | | | | | | | | | | | | |  |
| 1^st^ Lockdown (Mar – Apr 2020) | | | | □ | | | | | | | | | | | | | | | | | | | | | | | | | □ | | | | | | | | | | | | | | | | | | | | | | | | | | □ | | | | | | | | | | | | | | | | | | | |  |
| Interim Phase May Oct 2020) | | | | □ | | | | | | | | | | | | | | | | | | | | | | | | | □ | | | | | | | | | | | | | | | | | | | | | | | | | | □ | | | | | | | | | | | | | | | | | | | |  |
| 2^nd^ Lockdown (Nov 2020 – Feb 2021) | | | | □ | | | | | | | | | | | | | | | | | | | | | | | | | □ | | | | | | | | | | | | | | | | | | | | | | | | | | □ | | | | | | | | | | | | | | | | | | | |  |
| 41. Did or do the visiting arrangements pose a particular challenge for your team? | | | | | | | | | | | | | | | | | | | | | | | | | | | | | | | | | | | | | | | | | | | | | | | | | | | | | | | | | | | | | | | | | | | | | | | | | | |  |
|  | | | Yes | | | | | | | | | | | | | | | | | | | | | | | Partly | | | | | | | | | | | | | | | | | | | | | | | | | | | | | | No | | | | | | | | | | | | | | | | | | |  |
| 1^st^ Lockdown (Mar – Apr 2020) | | | □ | | | | | | | | | | | | | | | | | | | | | | | □ | | | | | | | | | | | | | | | | | | | | | | | | | | | | | | □ | | | | | | | | | | | | | | | | | | |  |
| Interim Phase May Oct 2020) | | | □ | | | | | | | | | | | | | | | | | | | | | | | □ | | | | | | | | | | | | | | | | | | | | | | | | | | | | | | □ | | | | | | | | | | | | | | | | | | |  |
| 2^nd^ Lockdown (Nov 2020 – Feb 2021) | | | □ | | | | | | | | | | | | | | | | | | | | | | | □ | | | | | | | | | | | | | | | | | | | | | | | | | | | | | | □ | | | | | | | | | | | | | | | | | | |  |
| 42. What is the process after the death of patients in the hospital in pandemic times?? | | | | | | | | | | | | | | | | | | | | | | | | | | | | | | | | | | | | | | | | | | | | | | | | | | | | | | | | | | | | | | | | | | | | | | | | | | |  |
|  | | | 1^st^ Lockdown (Mar – Apr 2020) | | | | | | | | | | | | | | | | | | | | | | | Interim Phase May Oct 2020) | | | | | | | | | | | | | | | | | | | | | | | | | | | | | | 2^nd^ Lockdown (Nov 2020 – Feb 2021) | | | | | | | | | | | | | | | | | | |  |
| Relatives may still say goodbye in person | | | □ | | | | | | | | | | | | | | | | | | | | | | | □ | | | | | | | | | | | | | | | | | | | | | | | | | | | | | | □ | | | | | | | | | | | | | | | | | | |  |
| A limited number of people are allowed to say goodbye | | | □ | | | | | | | | | | | | | | | | | | | | | | | □ | | | | | | | | | | | | | | | | | | | | | | | | | | | | | | □ | | | | | | | | | | | | | | | | | | |  |
| Outside the ward, a personal farewell is made possible | | | □ | | | | | | | | | | | | | | | | | | | | | | | □ | | | | | | | | | | | | | | | | | | | | | | | | | | | | | | □ | | | | | | | | | | | | | | | | | | |  |
| A digital farewell is possible for all patients | | | □ | | | | | | | | | | | | | | | | | | | | | | | □ | | | | | | | | | | | | | | | | | | | | | | | | | | | | | | □ | | | | | | | | | | | | | | | | | | |  |
| A personal farewell is no longer possible for all patients | | | □ | | | | | | | | | | | | | | | | | | | | | | | □ | | | | | | | | | | | | | | | | | | | | | | | | | | | | | | □ | | | | | | | | | | | | | | | | | | |  |
| For those who have died of COVID-19, it is not possible to say goodbye in person | | | □ | | | | | | | | | | | | | | | | | | | | | | | □ | | | | | | | | | | | | | | | | | | | | | | | | | | | | | | □ | | | | | | | | | | | | | | | | | | |  |
| A purely digital farewell is possible for COVID-19 patients | | | □ | | | | | | | | | | | | | | | | | | | | | | | □ | | | | | | | | | | | | | | | | | | | | | | | | | | | | | | □ | | | | | | | | | | | | | | | | | | |  |
| 43. Have you developed additional services for saying goodbye? If so, can you please describe them briefly (e. g. photos)? | | | | | | | | | | | | | | | | | | | | | | | | | | | | | | | | | | | | | | | | | | | | | | | | | | | | | | | | | | | | | | | | | | | | | | | | | | |  |
|  | | | | | | | | | | | | | | | | | | | | | | | | | | | | | | | | | | | | | | | | | | | | | | | | | | | | | | | | | | | | | | | | | | | | | | | | | | |  |
| 44. Has there been or will there be triage in your hospital in the event of insufficient resources in the intensive care unit or in the event of need for ventilation support? | | | | | | | | | | | | | | | | | | | | | | | | | | | | | | | | | | | | | | | | | | | | | | | | | | | | | | | | | | | | | | | | | | | | | | | | | | | |
|  | Yes | | | | | | | | | | | | | | | | | | | | | | No | | | | | | | | | | | | | | | | | | | | | | | | | | Not necessary so far | | | | | | | | | | | | | | | | | | | | | | | | | | |
| 1^st^ Lockdown (Mar – Apr 2020) |  | | | | | | | | | | | | | | | | | | | | | |  | | | | | | | | | | | | | | | | | | | | | | | | | |  | | | | | | | | | | | | | | | | | | | | | | | | | | |
| Interim Phase May Oct 2020) |  | | | | | | | | | | | | | | | | | | | | | |  | | | | | | | | | | | | | | | | | | | | | | | | | |  | | | | | | | | | | | | | | | | | | | | | | | | | | |
| 2^nd^ Lockdown (Nov 2020 – Feb 2021) |  | | | | | | | | | | | | | | | | | | | | | |  | | | | | | | | | | | | | | | | | | | | | | | | | |  | | | | | | | | | | | | | | | | | | | | | | | | | | |
| 45. How often was staff from your team involved in triage decisions? | | | | | | | | | | | | | | | | | | | | | | | | | | | | | | | | | | | | | | | | | | | | | | | | | | | | | | | | | | | | | | | | | | | | | | | | | | | |
|  | Always | | | | | | | | | | | | | | | | Sometimes | | | | | | | | | | | | | | | | | | | Never | | | | | | | | | | | | | | | | | | | | | | | | Not necessary so far | | | | | | | | | | | | | | | |
| 1^st^ Lockdown (Mar – Apr 2020) | □ | | | | | | | | | | | | | | | | □ | | | | | | | | | | | | | | | | | | | □ | | | | | | | | | | | | | | | | | | | | | | | | □ | | | | | | | | | | | | | | | |
| Interim Phase May Oct 2020) | □ | | | | | | | | | | | | | | | | □ | | | | | | | | | | | | | | | | | | | □ | | | | | | | | | | | | | | | | | | | | | | | | □ | | | | | | | | | | | | | | | |
| 2^nd^ Lockdown (Nov 2020 – Feb 2021) | □ | | | | | | | | | | | | | | | | □ | | | | | | | | | | | | | | | | | | | □ | | | | | | | | | | | | | | | | | | | | | | | | □ | | | | | | | | | | | | | | | |
| Is there anything else you would like to tell us about triage? | | | | | | | | | | | | | | | | | | | | | | | | | | | | | | | | | | | | | | | | | | | | | | | | | | | | | | | | | | | | | | | | | | | | | | | | | | | |
|  | | | | | | | | | | | | | | | | | | | | | | | | | | | | | | | | | | | | | | | | | | | | | | | | | | | | | | | | | | | | | | | | | | | | | | | | | | | |
| 46. Has the COVID-19 pandemic had or will it have an impact on your service’s income? | | | | | | | | | | | | | | | | | | | | | | | | | | | | | | | | | | | | | | | | | | | | | | | | | | | | | | | | | | | | | | | | | | | | | | | | | | |  |
|  | | | Considerable decrease | | | | | | | Decrease | | | | | | | | | | | | | | | | No change | | | | | | | | | | | | | | Increase | | | | | | | | | | | | | | | | Considerable increase | | | | | | | | | | | | | | | | | Don’t know | |  |
| 1^st^ Lockdown (Mar – Apr 2020) | | | □ | | | | | | | □ | | | | | | | | | | | | | | | | □ | | | | | | | | | | | | | | □ | | | | | | | | | | | | | | | | □ | | | | | | | | | | | | | | | | | □ | |  |
| Interim Phase May Oct 2020) | | | □ | | | | | | | □ | | | | | | | | | | | | | | | | □ | | | | | | | | | | | | | | □ | | | | | | | | | | | | | | | | □ | | | | | | | | | | | | | | | | | □ | |  |
| 2^nd^ Lockdown (Nov 2020 – Feb 2021) | | | □ | | | | | | | □ | | | | | | | | | | | | | | | | □ | | | | | | | | | | | | | | □ | | | | | | | | | | | | | | | | □ | | | | | | | | | | | | | | | | | □ | |  |
| 47. Were you or are you concerned about financial losses due to the COVID-19 pandemic? | | | | | | | | | | | | | | | | | | | | | | | | | | | | | | | | | | | | | | | | | | | | | | | | | | | | | | | | | | | | | | | | | | | | | | | | | | |  |
|  | | | Not concerned at all | | | | | | | | | | Rather less concerned | | | | | | | | | | | | | | | | | | | Rather more concerned | | | | | | | | | | | | | | | Very much concerned | | | | | | | | | | | | | | | | | | | | | | N./a. | | | | | |  |
| 1^st^ Lockdown (Mar – Apr 2020) | | | □ | | | | | | | | | | □ | | | | | | | | | | | | | | | | | | | □ | | | | | | | | | | | | | | | □ | | | | | | | | | | | | | | | | | | | | | | □ | | | | | |  |
| Interim Phase May Oct 2020) | | | □ | | | | | | | | | | □ | | | | | | | | | | | | | | | | | | | □ | | | | | | | | | | | | | | | □ | | | | | | | | | | | | | | | | | | | | | | □ | | | | | |  |
| 2^nd^ Lockdown (Nov 2020 – Feb 2021) | | | □ | | | | | | | | | | □ | | | | | | | | | | | | | | | | | | | □ | | | | | | | | | | | | | | | □ | | | | | | | | | | | | | | | | | | | | | | □ | | | | | |  |
| 48. Did you or do you have staff shortages due to the COVID-19 pandemic? | | | | | | | | | | | | | | | | | | | | | | | | | | | | | | | | | | | | | | | | | | | | | | | | | | | | | | | | | | | | | | | | | | | | | | | | | | |  |
|  | | | Yes | | | | | | | | | | | | | | | | | | | | | | | | | | | | | | | | | | | | | No | | | | | | | | | | | | | | | | | | | | | | | | | | | | | | | | | | |  |
| 1^st^ Lockdown (Mar – Apr 2020) | | | □ | | | | | | | | | | | | | | | | | | | | | | | | | | | | | | | | | | | | | □ | | | | | | | | | | | | | | | | | | | | | | | | | | | | | | | | | | |  |
| Interim Phase May Oct 2020) | | | □ | | | | | | | | | | | | | | | | | | | | | | | | | | | | | | | | | | | | | □ | | | | | | | | | | | | | | | | | | | | | | | | | | | | | | | | | | |  |
| 2^nd^ Lockdown (Nov 2020 – Feb 2021) | | | □ | | | | | | | | | | | | | | | | | | | | | | | | | | | | | | | | | | | | | □ | | | | | | | | | | | | | | | | | | | | | | | | | | | | | | | | | | |  |
| 49. In which areas did you or do you have staff shortages? | | | | | | | | | | | | | | | | | | | | | | | | | | | | | | | | | | | | | | | | | | | | | | | | | | | | | | | | | | | | | | | | | | | | | | | | | | |  |
|  | | | | | | | | | | | | | | | | | | | | | | | | | | | | | | | | | | | | | | | | | | | | | | | | | | | | | | | | | | | | | | | | | | | | | | | | | | |  |
| 50. Did you have or do you have the possibility to access support from additional staff? (Multiple answers possible) | | | | | | | | | | | | | | | | | | | | | | | | | | | | | | | | | | | | | | | | | | | | | | | | | | | | | | | | | | | | | | | | | | | | | | | | | | |  |
|  | | | Yes, from other wards | | | | | | | Yes, from a temp agency | | | | | | | | | | | | | | | | Yes, from medical students | | | | | | | | | | | | | | Yes, from retired staff | | | | | | | | | | | | | | | | No, not at all | | | | | | | | | | | | | | | | | Other | |  |
| 1^st^ Lockdown (Mar – Apr 2020) | | | □ | | | | | | | □ | | | | | | | | | | | | | | | | □ | | | | | | | | | | | | | | □ | | | | | | | | | | | | | | | | □ | | | | | | | | | | | | | | | | | □ | |  |
| Interim Phase May Oct 2020) | | | □ | | | | | | | □ | | | | | | | | | | | | | | | | □ | | | | | | | | | | | | | | □ | | | | | | | | | | | | | | | | □ | | | | | | | | | | | | | | | | | □ | |  |
| 2^nd^ Lockdown (Nov 2020 – Feb 2021) | | | □ | | | | | | | □ | | | | | | | | | | | | | | | | □ | | | | | | | | | | | | | | □ | | | | | | | | | | | | | | | | □ | | | | | | | | | | | | | | | | | □ | |  |
| 51. Due to the COVID-19 pandemic, did you have or have you developed strategies for maintaining care in case of staff shortages (e. g. due to infected staff or quarantine for staff)? | | | | | | | | | | | | | | | | | | | | | | | | | | | | | | | | | | | | | | | | | | | | | | | | | | | | | | | | | | | | | | | | | | | | | | | | | | |  |
|  | | | Yes | | | | | | | | | | | | | | | | | | | | | | | No | | | | | | | | | | | | | | | | | | | | | | | | | | | | | | Don’t know | | | | | | | | | | | | | | | | | | |  |
| 1^st^ Lockdown (Mar – Apr 2020) | | | □ | | | | | | | | | | | | | | | | | | | | | | | □ | | | | | | | | | | | | | | | | | | | | | | | | | | | | | | □ | | | | | | | | | | | | | | | | | | |  |
| Interim Phase May Oct 2020) | | | □ | | | | | | | | | | | | | | | | | | | | | | | □ | | | | | | | | | | | | | | | | | | | | | | | | | | | | | | □ | | | | | | | | | | | | | | | | | | |  |
| 2^nd^ Lockdown (Nov 2020 – Feb 2021) | | | □ | | | | | | | | | | | | | | | | | | | | | | | □ | | | | | | | | | | | | | | | | | | | | | | | | | | | | | | □ | | | | | | | | | | | | | | | | | | |  |
| 52. Have the strategies been or are they written down as conceptual measures? | | | | | | | | | | | | | | | | | | | | | | | | | | | | | | | | | | | | | | | | | | | | | | | | | | | | | | | | | | | | | | | | | | | | | | | | | | |  |
|  | | | Yes | | | | | | | | | | | | | | | | | Only verbal | | | | | | | | | | | | | | | | | | | | Not at all | | | | | | | | | | | | | | | | | | | | | | | | Don`t know | | | | | | | | | | |  |
| 1^st^ Lockdown (Mar – Apr 2020) | | | □ | | | | | | | | | | | | | | | | | □ | | | | | | | | | | | | | | | | | | | | □ | | | | | | | | | | | | | | | | | | | | | | | | □ | | | | | | | | | | |  |
| Interim Phase May Oct 2020) | | | □ | | | | | | | | | | | | | | | | | □ | | | | | | | | | | | | | | | | | | | | □ | | | | | | | | | | | | | | | | | | | | | | | | □ | | | | | | | | | | |  |
| 2^nd^ Lockdown (Nov 2020 – Feb 2021) | | | □ | | | | | | | | | | | | | | | | | □ | | | | | | | | | | | | | | | | | | | | □ | | | | | | | | | | | | | | | | | | | | | | | | □ | | | | | | | | | | |  |
| 53. What did these strategies involve? | | | | | | | | | | | | | | | | | | | | | | | | | | | | | | | | | | | | | | | | | | | | | | | | | | | | | | | | | | | | | | | | | | | | | | | | | | |  |
|  | | | Yes | | | | | | | | | | | | | | | | | No | | | | | | | | | | | | | | | | | | | | Currently being implemented | | | | | | | | | | | | | | | | | | | | | | | | N./a. | | | | | | | | | | |  |
| Telework | | | □ | | | | | | | | | | | | | | | | | □ | | | | | | | | | | | | | | | | | | | | □ | | | | | | | | | | | | | | | | | | | | | | | | □ | | | | | | | | | | |  |
| Separation of work areas | | | □ | | | | | | | | | | | | | | | | | □ | | | | | | | | | | | | | | | | | | | | □ | | | | | | | | | | | | | | | | | | | | | | | | □ | | | | | | | | | | |  |
| Clustering of teams | | | □ | | | | | | | | | | | | | | | | | □ | | | | | | | | | | | | | | | | | | | | □ | | | | | | | | | | | | | | | | | | | | | | | | □ | | | | | | | | | | |  |
| Fixed teams in each shift | | | □ | | | | | | | | | | | | | | | | | □ | | | | | | | | | | | | | | | | | | | | □ | | | | | | | | | | | | | | | | | | | | | | | | □ | | | | | | | | | | |  |
| No common lunch breaks | | | □ | | | | | | | | | | | | | | | | | □ | | | | | | | | | | | | | | | | | | | | □ | | | | | | | | | | | | | | | | | | | | | | | | □ | | | | | | | | | | |  |
| Mono-disciplinary team meetings | | | □ | | | | | | | | | | | | | | | | | □ | | | | | | | | | | | | | | | | | | | | □ | | | | | | | | | | | | | | | | | | | | | | | | □ | | | | | | | | | | |  |
| Use of larger rooms for meetings | | | □ | | | | | | | | | | | | | | | | | □ | | | | | | | | | | | | | | | | | | | | □ | | | | | | | | | | | | | | | | | | | | | | | | □ | | | | | | | | | | |  |
| Interdisciplinary team meetings via Zoom other platforms | | |  | | | | | | | | | | | | | | | | |  | | | | | | | | | | | | | | | | | | | |  | | | | | | | | | | | | | | | | | | | | | | | |  | | | | | | | | | | |  |
| Travel ban (business trips) | | | □ | | | | | | | | | | | | | | | | | □ | | | | | | | | | | | | | | | | | | | | □ | | | | | | | | | | | | | | | | | | | | | | | | □ | | | | | | | | | | |  |
| Travel bans to high-risk areas | | | □ | | | | | | | | | | | | | | | | | □ | | | | | | | | | | | | | | | | | | | | □ | | | | | | | | | | | | | | | | | | | | | | | | □ | | | | | | | | | | |  |
| Multi-step plan | | | □ | | | | | | | | | | | | | | | | | □ | | | | | | | | | | | | | | | | | | | | □ | | | | | | | | | | | | | | | | | | | | | | | | □ | | | | | | | | | | |  |
| Closure of the PCU | | | □ | | | | | | | | | | | | | | | | | □ | | | | | | | | | | | | | | | | | | | | □ | | | | | | | | | | | | | | | | | | | | | | | | □ | | | | | | | | | | |  |
| 1^st^ Lockdown (Mar – Apr 2020) | | | □ | | | | | | | | | | | | | | | | | □ | | | | | | | | | | | | | | | | | | | | □ | | | | | | | | | | | | | | | | | | | | | | | | □ | | | | | | | | | | |  |
| Interim Phase May Oct 2020) | | | □ | | | | | | | | | | | | | | | | | □ | | | | | | | | | | | | | | | | | | | | □ | | | | | | | | | | | | | | | | | | | | | | | | □ | | | | | | | | | | |  |
| 2^nd^ Lockdown (Nov 2020 – Feb 2021) | | | □ | | | | | | | | | | | | | | | | | □ | | | | | | | | | | | | | | | | | | | | □ | | | | | | | | | | | | | | | | | | | | | | | | □ | | | | | | | | | | |  |
| 54. Have there been or are there shortages in any of the following in your service caused by the pandemic?  (Please tick as appropriate. Multiple answers possible) | | | | | | | | | | | | | | | | | | | | | | | | | | | | | | | | | | | | | | | | | | | | | | | | | | | | | | | | | | | | | | | | | | | | | | | | | | |  |
|  | | | 1^st^ Lockdown (Mar – Apr 2020) | | | | | | | | | | | | | | | | | | | | | | | Interim Phase May Oct 2020) | | | | | | | | | | | | | | | | | | | | | | | | | | | | | | 2^nd^ Lockdown (Nov 2020 – Feb 2021) | | | | | | | | | | | | | | | | | | |  |
| FFP mask | | | □ | | | | | | | | | | | | | | | | | | | | | | | □ | | | | | | | | | | | | | | | | | | | | | | | | | | | | | | □ | | | | | | | | | | | | | | | | | | |  |
| Face mask | | | □ | | | | | | | | | | | | | | | | | | | | | | | □ | | | | | | | | | | | | | | | | | | | | | | | | | | | | | | □ | | | | | | | | | | | | | | | | | | |  |
| Protective gown | | | □ | | | | | | | | | | | | | | | | | | | | | | | □ | | | | | | | | | | | | | | | | | | | | | | | | | | | | | | □ | | | | | | | | | | | | | | | | | | |  |
| Pairs of goggles/visors | | | □ | | | | | | | | | | | | | | | | | | | | | | | □ | | | | | | | | | | | | | | | | | | | | | | | | | | | | | | □ | | | | | | | | | | | | | | | | | | |  |
| Surgical gloves | | | □ | | | | | | | | | | | | | | | | | | | | | | | □ | | | | | | | | | | | | | | | | | | | | | | | | | | | | | | □ | | | | | | | | | | | | | | | | | | |  |
| Sanitiser (surfaces) | | | □ | | | | | | | | | | | | | | | | | | | | | | | □ | | | | | | | | | | | | | | | | | | | | | | | | | | | | | | □ | | | | | | | | | | | | | | | | | | |  |
| Sanitiser (hands) | | | □ | | | | | | | | | | | | | | | | | | | | | | | □ | | | | | | | | | | | | | | | | | | | | | | | | | | | | | | □ | | | | | | | | | | | | | | | | | | |  |
| Tests/swabs | | | □ | | | | | | | | | | | | | | | | | | | | | | | □ | | | | | | | | | | | | | | | | | | | | | | | | | | | | | | □ | | | | | | | | | | | | | | | | | | |  |
| Were there or are there shortages of medicines or of other things? | | | | | | | | | | | | | | | | | | | | | | | | | | | | | | | | | | | | | | | | | | | | | | | | | | | | | | | | | | | | | | | | | | | | | | | | | | |  |
|  | | | | | | | | | | | | | | | | | | | | | | | | | | | | | | | | | | | | | | | | | | | | | | | | | | | | | | | | | | | | | | | | | | | | | | | | | | |  |
| 55. How much stress does the team of your service feel in times of pandemic due to the following factors? | | | | | | | | | | | | | | | | | | | | | | | | | | | | | | | | | | | | | | | | | | | | | | | | | | | | | | | | | | | | | | | | | | | | | | | | | | |  |
|  | | | None at all | | | | | | | | | | A little | | | | | | | | | | | | | | | | | | | Much | | | | | | | | | | | | | | | Very much | | | | | | | | | | | | | | | | | | | | | | Don’t know | | | | | |  |
| No time to be able to respond to the wishes and problems of those affected | | | □ | | | | | | | | | | □ | | | | | | | | | | | | | | | | | | | □ | | | | | | | | | | | | | | | □ | | | | | | | | | | | | | | | | | | | | | | □ | | | | | |  |
| Too little time (less than 3 days) until death of patients | | | □ | | | | | | | | | | □ | | | | | | | | | | | | | | | | | | | □ | | | | | | | | | | | | | | | □ | | | | | | | | | | | | | | | | | | | | | | □ | | | | | |  |
| Unexpected death of patients | | | □ | | | | | | | | | | □ | | | | | | | | | | | | | | | | | | | □ | | | | | | | | | | | | | | | □ | | | | | | | | | | | | | | | | | | | | | | □ | | | | | |  |
| Conflicting treatment plans in the care network | | | □ | | | | | | | | | | □ | | | | | | | | | | | | | | | | | | | □ | | | | | | | | | | | | | | | □ | | | | | | | | | | | | | | | | | | | | | | □ | | | | | |  |
| Insufficient symptom relief | | | □ | | | | | | | | | | □ | | | | | | | | | | | | | | | | | | | □ | | | | | | | | | | | | | | | □ | | | | | | | | | | | | | | | | | | | | | | □ | | | | | |  |
| Too high own demands on one's own work | | | □ | | | | | | | | | | □ | | | | | | | | | | | | | | | | | | | □ | | | | | | | | | | | | | | | □ | | | | | | | | | | | | | | | | | | | | | | □ | | | | | |  |
| Omission of physical contact and distance rules (patients) | | | □ | | | | | | | | | | □ | | | | | | | | | | | | | | | | | | | □ | | | | | | | | | | | | | | | □ | | | | | | | | | | | | | | | | | | | | | | □ | | | | | |  |
| Communication difficulties due to wearing masks | | | □ | | | | | | | | | | □ | | | | | | | | | | | | | | | | | | | □ | | | | | | | | | | | | | | | □ | | | | | | | | | | | | | | | | | | | | | | □ | | | | | |  |
| Distance rules (visitors/relatives) | | | □ | | | | | | | | | | □ | | | | | | | | | | | | | | | | | | | □ | | | | | | | | | | | | | | | □ | | | | | | | | | | | | | | | | | | | | | | □ | | | | | |  |
| Too little time for rituals (commemoration/ saying goodbye) | | | □ | | | | | | | | | | □ | | | | | | | | | | | | | | | | | | | □ | | | | | | | | | | | | | | | □ | | | | | | | | | | | | | | | | | | | | | | □ | | | | | |  |
| No on-site support from family of patients | | | □ | | | | | | | | | | □ | | | | | | | | | | | | | | | | | | | □ | | | | | | | | | | | | | | | □ | | | | | | | | | | | | | | | | | | | | | | □ | | | | | |  |
| Too few staff (incl. vacancies) | | | □ | | | | | | | | | | □ | | | | | | | | | | | | | | | | | | | □ | | | | | | | | | | | | | | | □ | | | | | | | | | | | | | | | | | | | | | | □ | | | | | |  |
| 56. How much strain does your team feel in times of pandemic due to the following factors in the working environment? | | | | | | | | | | | | | | | | | | | | | | | | | | | | | | | | | | | | | | | | | | | | | | | | | | | | | | | | | | | | | | | | | | | | | | | | | | |  |
|  | | | None at all | | | | | | | | | | | A little | | | | | | | | | | | | | | | | | | | Much | | | | | | | | | | | | Very much | | | | | | | | | | | | | | | | | | | | | Don’t know | | | | | | | | |  |
| Increased workload | | | □ | | | | | | | | | | | □ | | | | | | | | | | | | | | | | | | | □ | | | | | | | | | | | | □ | | | | | | | | | | | | | | | | | | | | | □ | | | | | | | | |  |
| Increased sick leaves | | | □ | | | | | | | | | | | □ | | | | | | | | | | | | | | | | | | | □ | | | | | | | | | | | | □ | | | | | | | | | | | | | | | | | | | | | □ | | | | | | | | |  |
| Conflicts within the team | | | □ | | | | | | | | | | | □ | | | | | | | | | | | | | | | | | | | □ | | | | | | | | | | | | □ | | | | | | | | | | | | | | | | | | | | | □ | | | | | | | | |  |
| Implementation of stricter hygiene regulations | | | □ | | | | | | | | | | | □ | | | | | | | | | | | | | | | | | | | □ | | | | | | | | | | | | □ | | | | | | | | | | | | | | | | | | | | | □ | | | | | | | | |  |
| Less contact and proximity to colleagues | | | □ | | | | | | | | | | | □ | | | | | | | | | | | | | | | | | | | □ | | | | | | | | | | | | □ | | | | | | | | | | | | | | | | | | | | | □ | | | | | | | | |  |
| Concern about getting infected | | | □ | | | | | | | | | | | □ | | | | | | | | | | | | | | | | | | | □ | | | | | | | | | | | | □ | | | | | | | | | | | | | | | | | | | | | □ | | | | | | | | |  |
| Concern about infecting others | | | □ | | | | | | | | | | | □ | | | | | | | | | | | | | | | | | | | □ | | | | | | | | | | | | □ | | | | | | | | | | | | | | | | | | | | | □ | | | | | | | | |  |
| Closure of wards/beds | | | □ | | | | | | | | | | | □ | | | | | | | | | | | | | | | | | | | □ | | | | | | | | | | | | □ | | | | | | | | | | | | | | | | | | | | | □ | | | | | | | | |  |
| Deployment of employees in the COVID-19 area | | | □ | | | | | | | | | | | □ | | | | | | | | | | | | | | | | | | | □ | | | | | | | | | | | | □ | | | | | | | | | | | | | | | | | | | | | □ | | | | | | | | |  |
| Lack of multi-professional cooperation within the team | | | □ | | | | | | | | | | | □ | | | | | | | | | | | | | | | | | | | □ | | | | | | | | | | | | □ | | | | | | | | | | | | | | | | | | | | | □ | | | | | | | | |  |
| Lack of cooperation with network partners | | | □ | | | | | | | | | | | □ | | | | | | | | | | | | | | | | | | | □ | | | | | | | | | | | | □ | | | | | | | | | | | | | | | | | | | | | □ | | | | | | | | |  |
| Spirit of palliative care pushed back by Covid-19 | | | □ | | | | | | | | | | | □ | | | | | | | | | | | | | | | | | | | □ | | | | | | | | | | | | □ | | | | | | | | | | | | | | | | | | | | | □ | | | | | | | | |  |
| 57. In your opinion, have working conditions at your workplace improved or worsened since the COVID-19 outbreak? | | | | | | | | | | | | | | | | | | | | | | | | | | | | | | | | | | | | | | | | | | | | | | | | | | | | | | | | | | | | | | | | | | | | | | | | | | |  |
|  | | | Considerably improved | | | | | | | | Improved | | | | | | | | | | | | | | | | | Remained unchanged | | | | | | | | | | | | | Worsened | | | | | | | | | | | | | Considerably worsened | | | | | | | | | | | | | | | | | Don’t know | | | |  |
| 1^st^ Lockdown (Mar – Apr 2020) | | | □ | | | | | | | | □ | | | | | | | | | | | | | | | | | □ | | | | | | | | | | | | | □ | | | | | | | | | | | | | □ | | | | | | | | | | | | | | | | | □ | | | |  |
| Interim Phase May Oct 2020) | | | □ | | | | | | | | □ | | | | | | | | | | | | | | | | | □ | | | | | | | | | | | | | □ | | | | | | | | | | | | | □ | | | | | | | | | | | | | | | | | □ | | | |  |
| 2^nd^ Lockdown (Nov 2020 – Feb 2021) | | | □ | | | | | | | | □ | | | | | | | | | | | | | | | | | □ | | | | | | | | | | | | | □ | | | | | | | | | | | | | □ | | | | | | | | | | | | | | | | | □ | | | |  |
| 58. In your opinion, has the care of people with palliative care needs improved or worsened as a result of the COVID-19 pandemic? | | | | | | | | | | | | | | | | | | | | | | | | | | | | | | | | | | | | | | | | | | | | | | | | | | | | | | | | | | | | | | | | | | | | | | | | | | |  |
|  | | | Considerably improved | | | | | | | | Improved | | | | | | | | | | | | | | | | | Remained unchanged | | | | | | | | | | | | | Worsened | | | | | | | | | | | | | Considerably worsened | | | | | | | | | | | | | | | | | Don’t know | | | |  |
| 1^st^ Lockdown (Mar – Apr 2020) | | | □ | | | | | | | | □ | | | | | | | | | | | | | | | | | □ | | | | | | | | | | | | | □ | | | | | | | | | | | | | □ | | | | | | | | | | | | | | | | | □ | | | |  |
| Interim Phase May Oct 2020) | | | □ | | | | | | | | □ | | | | | | | | | | | | | | | | | □ | | | | | | | | | | | | | □ | | | | | | | | | | | | | □ | | | | | | | | | | | | | | | | | □ | | | |  |
| 2^nd^ Lockdown (Nov 2020 – Feb 2021) | | | □ | | | | | | | | □ | | | | | | | | | | | | | | | | | □ | | | | | | | | | | | | | □ | | | | | | | | | | | | | □ | | | | | | | | | | | | | | | | | □ | | | |  |
| 59. In the current pandemic situation, how would you rate the cooperation with...? (German marks) | | | | | | | | | | | | | | | | | | | | | | | | | | | | | | | | | | | | | | | | | | | | | | | | | | | | | | | | | | | | | | | | | | | | | | | | | | |  |
|  | | | Excellent | | | | | | | | Good | | | | | | | | | | | | | | | | | Satisfactory | | | | | | | | | | | | | Sufficient | | | | | | | | | | | | | Inadequate | | | | | | | | | | | | | | | | | Failed | | | |  |
| Colleagues in cases of internal transitions | | |  | | | | | | | |  | | | | | | | | | | | | | | | | |  | | | | | | | | | | | | |  | | | | | | | | | | | | |  | | | | | | | | | | | | | | | | |  | | | |  |
| General practitioners | | | □ | | | | | | | | □ | | | | | | | | | | | | | | | | | □ | | | | | | | | | | | | | □ | | | | | | | | | | | | | □ | | | | | | | | | | | | | | | | | □ | | | |  |
| Specialist registrar/consultant | | | □ | | | | | | | | □ | | | | | | | | | | | | | | | | | □ | | | | | | | | | | | | | □ | | | | | | | | | | | | | □ | | | | | | | | | | | | | | | | | □ | | | |  |
| Home specialist palliative care teams | | | □ | | | | | | | | □ | | | | | | | | | | | | | | | | | □ | | | | | | | | | | | | | □ | | | | | | | | | | | | | □ | | | | | | | | | | | | | | | | | □ | | | |  |
| Home nursing care services | | | □ | | | | | | | | □ | | | | | | | | | | | | | | | | | □ | | | | | | | | | | | | | □ | | | | | | | | | | | | | □ | | | | | | | | | | | | | | | | | □ | | | |  |
| Hospice and palliative care volunteer services | | | □ | | | | | | | | □ | | | | | | | | | | | | | | | | | □ | | | | | | | | | | | | | □ | | | | | | | | | | | | | □ | | | | | | | | | | | | | | | | | □ | | | |  |
| Physiotherapists, occupational therapists | | | □ | | | | | | | | □ | | | | | | | | | | | | | | | | | □ | | | | | | | | | | | | | □ | | | | | | | | | | | | | □ | | | | | | | | | | | | | | | | | □ | | | |  |
| Care homes/nursing homes | | | □ | | | | | | | | □ | | | | | | | | | | | | | | | | | □ | | | | | | | | | | | | | □ | | | | | | | | | | | | | □ | | | | | | | | | | | | | | | | | □ | | | |  |
| Inpatient hospices | | | □ | | | | | | | | □ | | | | | | | | | | | | | | | | | □ | | | | | | | | | | | | | □ | | | | | | | | | | | | | □ | | | | | | | | | | | | | | | | | □ | | | |  |
| Other palliative care units | | | □ | | | | | | | | □ | | | | | | | | | | | | | | | | | □ | | | | | | | | | | | | | □ | | | | | | | | | | | | | □ | | | | | | | | | | | | | | | | | □ | | | |  |
| Public health departments | | | □ | | | | | | | | □ | | | | | | | | | | | | | | | | | □ | | | | | | | | | | | | | □ | | | | | | | | | | | | | □ | | | | | | | | | | | | | | | | | □ | | | |  |
| Funeral homes | | | □ | | | | | | | | □ | | | | | | | | | | | | | | | | | □ | | | | | | | | | | | | | □ | | | | | | | | | | | | | □ | | | | | | | | | | | | | | | | | □ | | | |  |
| 60. How difficult or easy is it to organise follow-up home care in times of the COVID-19 pandemic? | | | | | | | | | | | | | | | | | | | | | | | | | | | | | | | | | | | | | | | | | | | | | | | | | | | | | | | | | | | | | | | | | | | | | | | | | | |  |
|  | | | Very difficult | | | | | | | | Difficult | | | | | | | | | | | | | | | | | Remained unchanged | | | | | | | | | | | | | Easy | | | | | | | | | | | | | Very easy | | | | | | | | | | | | | | | | | Don’t know | | | |  |
| 1^st^ Lockdown (Mar – Apr 2020) | | | □ | | | | | | | | □ | | | | | | | | | | | | | | | | | □ | | | | | | | | | | | | | □ | | | | | | | | | | | | | □ | | | | | | | | | | | | | | | | | □ | | | |  |
| Interim Phase May Oct 2020) | | | □ | | | | | | | | □ | | | | | | | | | | | | | | | | | □ | | | | | | | | | | | | | □ | | | | | | | | | | | | | □ | | | | | | | | | | | | | | | | | □ | | | |  |
| 2^nd^ Lockdown (Nov 2020 – Feb 2021) | | | □ | | | | | | | | □ | | | | | | | | | | | | | | | | | □ | | | | | | | | | | | | | □ | | | | | | | | | | | | | □ | | | | | | | | | | | | | | | | | □ | | | |  |
| 61. How difficult or easy is it to organise follow-up inpatient care in times of the COVID-19 pandemic? | | | | | | | | | | | | | | | | | | | | | | | | | | | | | | | | | | | | | | | | | | | | | | | | | | | | | | | | | | | | | | | | | | | | | | | | | | |  |
|  | | | Very difficult | | | | | | | | Difficult | | | | | | | | | | | | | | | | | Remained unchanged | | | | | | | | | | | | | Easy | | | | | | | | | | | | | Very easy | | | | | | | | | | | | | | | | | Don’t know | | | |  |
| 1^st^ Lockdown (Mar – Apr 2020) | | | □ | | | | | | | | □ | | | | | | | | | | | | | | | | | □ | | | | | | | | | | | | | □ | | | | | | | | | | | | | □ | | | | | | | | | | | | | | | | | □ | | | |  |
| Interim Phase May Oct 2020) | | | □ | | | | | | | | □ | | | | | | | | | | | | | | | | | □ | | | | | | | | | | | | | □ | | | | | | | | | | | | | □ | | | | | | | | | | | | | | | | | □ | | | |  |
| 2^nd^ Lockdown (Nov 2020 – Feb 2021) | | | □ | | | | | | | | □ | | | | | | | | | | | | | | | | | □ | | | | | | | | | | | | | □ | | | | | | | | | | | | | □ | | | | | | | | | | | | | | | | | □ | | | |  |
| Can you please share with us some of the challenges encountered during admission, discharge or organising care after discharge? | | | | | | | | | | | | | | | | | | | | | | | | | | | | | | | | | | | | | | | | | | | | | | | | | | | | | | | | | | | | | | | | | | | | | | | | | | |  |
|  | | | | | | | | | | | | | | | | | | | | | | | | | | | | | | | | | | | | | | | | | | | | | | | | | | | | | | | | | | | | | | | | | | | | | | | | | | |  |
| 62. How important are the following team strengthening factors for your team and do they continue to take place? | | | | | | | | | | | | | | | | | | | | | | | | | | | | | | | | | | | | | | | | | | | | | | | | | | | | | | | | | | | | | | | | | | | | | | | | | | |  |
|  | | | Not important at all | | | | | Less important | | | | | | | | | | | | | Important | | | | | | | | | | | | | | Very important | | | | | | | | | Currently discontin-ued/ lacking | | | | | | | | | | | | | Currently ongoing/ not lacking | | | | | | | | | | | | | | | N./a. | | | |
| Supervision | | | □ | | | | | □ | | | | | | | | | | | | | □ | | | | | | | | | | | | | | □ | | | | | | | | | □ | | | | | | | | | | | | | □ | | | | | | | | | | | | | | | □ | | | |
| Rituals | | | □ | | | | | □ | | | | | | | | | | | | | □ | | | | | | | | | | | | | | □ | | | | | | | | | □ | | | | | | | | | | | | | □ | | | | | | | | | | | | | | | □ | | | |
| Humour | | | □ | | | | | □ | | | | | | | | | | | | | □ | | | | | | | | | | | | | | □ | | | | | | | | | □ | | | | | | | | | | | | | □ | | | | | | | | | | | | | | | □ | | | |
| Meeting for lunch breaks | | |  | | | | |  | | | | | | | | | | | | |  | | | | | | | | | | | | | |  | | | | | | | | |  | | | | | | | | | | | | |  | | | | | | | | | | | | | | |  | | | |
| Faith/religion | | | □ | | | | | □ | | | | | | | | | | | | | □ | | | | | | | | | | | | | | □ | | | | | | | | | □ | | | | | | | | | | | | | □ | | | | | | | | | | | | | | | □ | | | |
| Team activities (e.g. excursions, celebrations) | | | □ | | | | | □ | | | | | | | | | | | | | □ | | | | | | | | | | | | | | □ | | | | | | | | | □ | | | | | | | | | | | | | □ | | | | | | | | | | | | | | | □ | | | |
| Team sport | | | □ | | | | | □ | | | | | | | | | | | | | □ | | | | | | | | | | | | | | □ | | | | | | | | | □ | | | | | | | | | | | | | □ | | | | | | | | | | | | | | | □ | | | |
| Team meetings | | | □ | | | | | □ | | | | | | | | | | | | | □ | | | | | | | | | | | | | | □ | | | | | | | | | □ | | | | | | | | | | | | | □ | | | | | | | | | | | | | | | □ | | | |
| Commemorations / Hours of Remembrance | | | □ | | | | | □ | | | | | | | | | | | | | □ | | | | | | | | | | | | | | □ | | | | | | | | | □ | | | | | | | | | | | | | □ | | | | | | | | | | | | | | | □ | | | |
| Interdisciplinary handover talks | | | □ | | | | | □ | | | | | | | | | | | | | □ | | | | | | | | | | | | | | □ | | | | | | | | | □ | | | | | | | | | | | | | □ | | | | | | | | | | | | | | | □ | | | |
| Case discussions with volunteers, home specialist palliative care teams and other cooperating services | | | □ | | | | | □ | | | | | | | | | | | | | □ | | | | | | | | | | | | | | □ | | | | | | | | | □ | | | | | | | | | | | | | □ | | | | | | | | | | | | | | | □ | | | |
| 63. Did the institution offer special support or appreciative measures for employees due to the pandemic (e. g. emergency child care, bonuses, etc.)? | | | | | | | | | | | | | | | | | | | | | | | | | | | | | | | | | | | | | | | | | | | | | | | | | | | | | | | | | | | | | | | | | | | | | | | | | | | |
|  | | | Yes | | | | | | | | | | | | | | | | | | | | | | | No | | | | | | | | | | | | | | | | | | | | | | | | | | | | | | Don’t know | | | | | | | | | | | | | | | | | | | |
| 1^st^ Lockdown (Mar – Apr 2020) | | | □ | | | | | | | | | | | | | | | | | | | | | | | □ | | | | | | | | | | | | | | | | | | | | | | | | | | | | | | □ | | | | | | | | | | | | | | | | | | | |
| Interim Phase May Oct 2020) | | | □ | | | | | | | | | | | | | | | | | | | | | | | □ | | | | | | | | | | | | | | | | | | | | | | | | | | | | | | □ | | | | | | | | | | | | | | | | | | | |
| 2^nd^ Lockdown (Nov 2020 – Feb 2021) | | | □ | | | | | | | | | | | | | | | | | | | | | | | □ | | | | | | | | | | | | | | | | | | | | | | | | | | | | | | □ | | | | | | | | | | | | | | | | | | | |
| Would you like to tell us which of the offers were particularly helpful? | | | | | | | | | | | | | | | | | | | | | | | | | | | | | | | | | | | | | | | | | | | | | | | | | | | | | | | | | | | | | | | | | | | | | | | | | | | |
|  | | | | | | | | | | | | | | | | | | | | | | | | | | | | | | | | | | | | | | | | | | | | | | | | | | | | | | | | | | | | | | | | | | | | | | | | | | | |
| 64. How well do you feel informed by the following institutions or contact persons? | | | | | | | | | | | | | | | | | | | | | | | | | | | | | | | | | | | | | | | | | | | | | | | | | | | | | | | | | | | | | | | | | | | | | | | | | | | |
|  | | | Excellent | | | | | | | | Well | | | | | | | | | | | | | | | | | Bad | | | | | | | | | | | | | Very bad | | | | | | | | | | | | | Currently better than at the begin of the pandemic | | | | | | | | | | | | | | | | | No contact person | | | | |
| Hospital management | | | □ | | | | | | | | □ | | | | | | | | | | | | | | | | | □ | | | | | | | | | | | | | □ | | | | | | | | | | | | | □ | | | | | | | | | | | | | | | | | □ | | | | |
| Covid-19 crisis committee | | | □ | | | | | | | | □ | | | | | | | | | | | | | | | | | □ | | | | | | | | | | | | | □ | | | | | | | | | | | | | □ | | | | | | | | | | | | | | | | | □ | | | | |
| Public health department | | | □ | | | | | | | | □ | | | | | | | | | | | | | | | | | □ | | | | | | | | | | | | | □ | | | | | | | | | | | | | □ | | | | | | | | | | | | | | | | | □ | | | | |
| Hygiene manager | | | □ | | | | | | | | □ | | | | | | | | | | | | | | | | | □ | | | | | | | | | | | | | □ | | | | | | | | | | | | | □ | | | | | | | | | | | | | | | | | □ | | | | |
| 65. Did you and your team receive training specifically on COVID-19, hygiene and protection measures?? | | | | | | | | | | | | | | | | | | | | | | | | | | | | | | | | | | | | | | | | | | | | | | | | | | | | | | | | | | | | | | | | | | | | | | | | | | | |
|  | | | Yes | | | | | | | | | | | | | | | | | | | | | | | | | No | | | | | | | | | | | | | | | | | | | | | | | | | | Don’t know | | | | | | | | | | | | | | | | | | | | | |
| Live-online trainings | | | □ | | | | | | | | | | | | | | | | | | | | | | | | | □ | | | | | | | | | | | | | | | | | | | | | | | | | | □ | | | | | | | | | | | | | | | | | | | | | |
| Video trainings and podcasts | | | □ | | | | | | | | | | | | | | | | | | | | | | | | | □ | | | | | | | | | | | | | | | | | | | | | | | | | | □ | | | | | | | | | | | | | | | | | | | | | |
| Classroom training | | | □ | | | | | | | | | | | | | | | | | | | | | | | | | □ | | | | | | | | | | | | | | | | | | | | | | | | | | □ | | | | | | | | | | | | | | | | | | | | | |
| Written training material and brochures | | | □ | | | | | | | | | | | | | | | | | | | | | | | | | □ | | | | | | | | | | | | | | | | | | | | | | | | | | □ | | | | | | | | | | | | | | | | | | | | | |
| Information and instructions via e-mail | | | □ | | | | | | | | | | | | | | | | | | | | | | | | | □ | | | | | | | | | | | | | | | | | | | | | | | | | | □ | | | | | | | | | | | | | | | | | | | | | |
| 66. How well do you feel informed about vaccines, vaccination schedules and the organisation of vaccination procedures? | | | | | | | | | | | | | | | | | | | | | | | | | | | | | | | | | | | | | | | | | | | | | | | | | | | | | | | | | | | | | | | | | | | | | | | | | | | |
|  | | | Excellent | | | | | | | | | | | Well | | | | | | | | | | | | | | | | | | | Bad | | | | | | | | | | | | Very bad | | | | | | | | | | | | | | | | | | | | | Don’t know | | | | | | | | | |
| Vaccines | | | □ | | | | | | | | | | | □ | | | | | | | | | | | | | | | | | | | □ | | | | | | | | | | | | □ | | | | | | | | | | | | | | | | | | | | | □ | | | | | | | | | |
| Vaccination schedules | | | □ | | | | | | | | | | | □ | | | | | | | | | | | | | | | | | | | □ | | | | | | | | | | | | □ | | | | | | | | | | | | | | | | | | | | | □ | | | | | | | | | |
| Organisation of the vaccination procedures | | | □ | | | | | | | | | | | □ | | | | | | | | | | | | | | | | | | | □ | | | | | | | | | | | | □ | | | | | | | | | | | | | | | | | | | | | □ | | | | | | | | | |
| 67. What is the priority of your team in the vaccination schedule? | | | | | | | | | | | | | | | | | | | | | | | | | | | | | | | | | | | | | | | | | | | | | | | | | | | | | | | | | | | | | | | | | | | | | | | | | | | |
|  | | | Highest | | | | | | | | | | | | | | | | | Medium | | | | | | | | | | | | | | | | | | | | | Low | | | | | | | | | | | | | | | | | | | | | Don’t know | | | | | | | | | | | | | |
|  | | | □ | | | | | | | | | | | | | | | | | □ | | | | | | | | | | | | | | | | | | | | | □ | | | | | | | | | | | | | | | | | | | | | □ | | | | | | | | | | | | | |
| 68. Have the first staff members of your team been or will be vaccinated? | | | | | | | | | | | | | | | | | | | | | | | | | | | | | | | | | | | | | | | | | | | | | | | | | | | | | | | | | | | | | | | | | | | | | | | | | | | |
|  | | | Yes | | | | | | | | | | | | | | | | | | | | | | | | | No | | | | | | | | | | | | | | | | | | | | | | | | | | Appointments are made and we are next | | | | | | | | | | | | | | | | | | | | | |
|  | | | □ | | | | | | | | | | | | | | | | | | | | | | | | | □ | | | | | | | | | | | | | | | | | | | | | | | | | | □ | | | | | | | | | | | | | | | | | | | | | |
| 69. Please make a statement on the organisation of a clinical ethics committee in your institution | | | | | | | | | | | | | | | | | | | | | | | | | | | | | | | | | | | | | | | | | | | | | | | | | | | | | | | | | | | | | | | | | | | | | | | | | | | |
|  | | | There was already a clinical ethics committee in place before the pandemic | | | | | | | | | | | | | | | | | A clinical ethics committee was only introduced in the wake of the pandemic | | | | | | | | | | | | | | | | | | | | | No, there is still no clinical ethics committee at our hospital | | | | | | | | | | | | | | | | | | | | | Other | | | | | | | | | | | | | |
|  | | | □ | | | | | | | | | | | | | | | □ | | | | | | | | | | | | | | | | | | | | □ | | | | | | | | | | | | | | | | | | | | | □ | | | | | | | | | | | | | | | | |
| If other, can you please explain? | | | | | | | | | | | | | | | | | | | | | | | | | | | | | | | | | | | | | | | | | | | | | | | | | | | | | | | | | | | | | | | | | | | | | | | | | | | |
|  | | | | | | | | | | | | | | | | | | | | | | | | | | | | | | | | | | | | | | | | | | | | | | | | | | | | | | | | | | | | | | | | | | | | | | | | | | | |
| 70. Were or are representatives of your team regularly involved in a crisis team/committee? | | | | | | | | | | | | | | | | | | | | | | | | | | | | | | | | | | | | | | | | | | | | | | | | | | | | | | | | | | | | | | | | | | | | | | | | | |  |  |
|  | | | Yes | | | | | | | | | | | | | | | | | | | | | | | | No | | | | | | | | | | | | | | | | | | | | | | | | | | Partly | | | | | | | | | | | | | | | | | | | | |  |  |
| 1^st^ Lockdown (Mar – Apr 2020) | | | □ | | | | | | | | | | | | | | | | | | | | | | | | □ | | | | | | | | | | | | | | | | | | | | | | | | | | □ | | | | | | | | | | | | | | | | | | | | |  |  |
| Interim Phase May Oct 2020) | | | □ | | | | | | | | | | | | | | | | | | | | | | | | □ | | | | | | | | | | | | | | | | | | | | | | | | | | □ | | | | | | | | | | | | | | | | | | | | |  |  |
| 2^nd^ Lockdown (Nov 2020 – Feb 2021) | | | □ | | | | | | | | | | | | | | | | | | | | | | | | □ | | | | | | | | | | | | | | | | | | | | | | | | | | □ | | | | | | | | | | | | | | | | | | | | |  |  |
| 71. Was palliative medicine or palliative care proactively involved in the crisis team/committee from the outset? | | | | | | | | | | | | | | | | | | | | | | | | | | | | | | | | | | | | | | | | | | | | | | | | | | | | | | | | | | | | | | | | | | | | | | | | | |  |  |
|  | | | Yes, proactively | | | | | | | | | | | | | | | | Yes, but only after complaints | | | | | | | | | | | | | | | | | | | | | No | | | | | | | | | | | | | | | | | | | | | N./a. | | | | | | | | | | | | |  |  |
| 1^st^ Lockdown (Mar – Apr 2020) | | | □ | | | | | | | | | | | | | | | | □ | | | | | | | | | | | | | | | | | | | | | □ | | | | | | | | | | | | | | | | | | | | | □ | | | | | | | | | | | | |  |  |
| Interim Phase May Oct 2020) | | | □ | | | | | | | | | | | | | | | | □ | | | | | | | | | | | | | | | | | | | | | □ | | | | | | | | | | | | | | | | | | | | | □ | | | | | | | | | | | | |  |  |
| 2^nd^ Lockdown (Nov 2020 – Feb 2021) | | | □ | | | | | | | | | | | | | | | | □ | | | | | | | | | | | | | | | | | | | | | □ | | | | | | | | | | | | | | | | | | | | | □ | | | | | | | | | | | | |  |  |
| 72. What hygiene measures have been or are being introduced at your service since the beginning of the pandemic? | | | | | | | | | | | | | | | | | | | | | | | | | | | | | | | | | | | | | | | | | | | | | | | | | | | | | | | | | | | | | | | | | | | | | | | | | |  |  |
|  | | | Consistently implemented | | | | | | | | | | | Partly implemented | | | | | | | | | | | | | | | | | | | Not implemented | | | | | | | | | | | | Part of the action plan | | | | | | | | | | | | | | | | | | | | | Existed before the pandemic | | | | | | | |  |  |
| Basic hygiene measures | | | □ | | | | | | | | | | | □ | | | | | | | | | | | | | | | | | | | □ | | | | | | | | | | | | □ | | | | | | | | | | | | | | | | | | | | | □ | | | | | | | |  |  |
| Increased adherence to hand hygiene | | | □ | | | | | | | | | | | □ | | | | | | | | | | | | | | | | | | | □ | | | | | | | | | | | | □ | | | | | | | | | | | | | | | | | | | | | □ | | | | | | | |  |  |
| Face masks for staff | | | □ | | | | | | | | | | | □ | | | | | | | | | | | | | | | | | | | □ | | | | | | | | | | | | □ | | | | | | | | | | | | | | | | | | | | | □ | | | | | | | |  |  |
| Face masks for patients | | | □ | | | | | | | | | | | □ | | | | | | | | | | | | | | | | | | | □ | | | | | | | | | | | | □ | | | | | | | | | | | | | | | | | | | | | □ | | | | | | | |  |  |
| Face masks for visitors | | | □ | | | | | | | | | | | □ | | | | | | | | | | | | | | | | | | | □ | | | | | | | | | | | | □ | | | | | | | | | | | | | | | | | | | | | □ | | | | | | | |  |  |
| FFP2 masks | | | □ | | | | | | | | | | | □ | | | | | | | | | | | | | | | | | | | □ | | | | | | | | | | | | □ | | | | | | | | | | | | | | | | | | | | | □ | | | | | | | |  |  |
| Disinfection of surfaces | | | □ | | | | | | | | | | | □ | | | | | | | | | | | | | | | | | | | □ | | | | | | | | | | | | □ | | | | | | | | | | | | | | | | | | | | | □ | | | | | | | |  |  |
| Number of persons/room size | | | □ | | | | | | | | | | | □ | | | | | | | | | | | | | | | | | | | □ | | | | | | | | | | | | □ | | | | | | | | | | | | | | | | | | | | | □ | | | | | | | |  |  |
| Protective clothing (gown/ visor/ goggles/ double gloves) | | | □ | | | | | | | | | | | □ | | | | | | | | | | | | | | | | | | | □ | | | | | | | | | | | | □ | | | | | | | | | | | | | | | | | | | | | □ | | | | | | | |  |  |
| Visiting regulations or restrictions | | | □ | | | | | | | | | | | □ | | | | | | | | | | | | | | | | | | | □ | | | | | | | | | | | | □ | | | | | | | | | | | | | | | | | | | | | □ | | | | | | | |  |  |
| Ventilating/airing rooms | | | □ | | | | | | | | | | | □ | | | | | | | | | | | | | | | | | | | □ | | | | | | | | | | | | □ | | | | | | | | | | | | | | | | | | | | | □ | | | | | | | |  |  |
| Avoiding contact between patients | | | □ | | | | | | | | | | | □ | | | | | | | | | | | | | | | | | | | □ | | | | | | | | | | | | □ | | | | | | | | | | | | | | | | | | | | | □ | | | | | | | |  |  |
| Avoiding contact between visitors | | | □ | | | | | | | | | | | □ | | | | | | | | | | | | | | | | | | | □ | | | | | | | | | | | | □ | | | | | | | | | | | | | | | | | | | | | □ | | | | | | | |  |  |
| Distance rules or no contact | | | □ | | | | | | | | | | | □ | | | | | | | | | | | | | | | | | | | □ | | | | | | | | | | | | □ | | | | | | | | | | | | | | | | | | | | | □ | | | | | | | |  |  |
| No farewell rituals | | | □ | | | | | | | | | | | □ | | | | | | | | | | | | | | | | | | | □ | | | | | | | | | | | | □ | | | | | | | | | | | | | | | | | | | | | □ | | | | | | | |  |  |
| 73. What hygiene measures have been or are being introduced in your hospital/to your team since the beginning of the pandemic?? | | | | | | | | | | | | | | | | | | | | | | | | | | | | | | | | | | | | | | | | | | | | | | | | | | | | | | | | | | | | | | | | | | | | | | | | | | | |
|  | | | | | Consistently implemented | | | | | | | | | | Partly implemented | | | | | | | | | | | | | | | | | | | Not implemented | | | | | | | | | | | | Part of the action plan | | | | | | | | | | | | | | | | | | | | | Existed before the pandemic | | | | | | |  |  |
| Secured entry areas | | | | | □ | | | | | | | | | | □ | | | | | | | | | | | | | | | | | | | □ | | | | | | | | | | | | □ | | | | | | | | | | | | | | | | | | | | | □ | | | | | | |  |  |
| Separate entrances for staff and visitors | | | | | □ | | | | | | | | | | □ | | | | | | | | | | | | | | | | | | | □ | | | | | | | | | | | | □ | | | | | | | | | | | | | | | | | | | | | □ | | | | | | |  |  |
| Closure of common areas for patients | | | | | □ | | | | | | | | | | □ | | | | | | | | | | | | | | | | | | | □ | | | | | | | | | | | | □ | | | | | | | | | | | | | | | | | | | | | □ | | | | | | |  |  |
| Closure of common areas for visitors | | | | | □ | | | | | | | | | | □ | | | | | | | | | | | | | | | | | | | □ | | | | | | | | | | | | □ | | | | | | | | | | | | | | | | | | | | | □ | | | | | | |  |  |
| Minimising contact and communication with patients | | | | | □ | | | | | | | | | | □ | | | | | | | | | | | | | | | | | | | □ | | | | | | | | | | | | □ | | | | | | | | | | | | | | | | | | | | | □ | | | | | | |  |  |
| Minimising contact and communication with visitors | | | | | □ | | | | | | | | | | □ | | | | | | | | | | | | | | | | | | | □ | | | | | | | | | | | | □ | | | | | | | | | | | | | | | | | | | | | □ | | | | | | |  |  |
| Closure of common areas for staff | | | | | □ | | | | | | | | | | □ | | | | | | | | | | | | | | | | | | | □ | | | | | | | | | | | | □ | | | | | | | | | | | | | | | | | | | | | □ | | | | | | |  |  |
| Clustering of teams | | | | | □ | | | | | | | | | | □ | | | | | | | | | | | | | | | | | | | □ | | | | | | | | | | | | □ | | | | | | | | | | | | | | | | | | | | | □ | | | | | | |  |  |
| Mono-disciplinary team meetings | | | | | □ | | | | | | | | | | □ | | | | | | | | | | | | | | | | | | | □ | | | | | | | | | | | | □ | | | | | | | | | | | | | | | | | | | | | □ | | | | | | |  |  |
| Keeping visitor lists | | | | | □ | | | | | | | | | | □ | | | | | | | | | | | | | | | | | | | □ | | | | | | | | | | | | □ | | | | | | | | | | | | | | | | | | | | | □ | | | | | | |  |  |
| Staff keep contact lists | | | | | □ | | | | | | | | | | □ | | | | | | | | | | | | | | | | | | | □ | | | | | | | | | | | | □ | | | | | | | | | | | | | | | | | | | | | □ | | | | | | |  |  |
| Access only with registration and admission by staff | | | | | □ | | | | | | | | | | □ | | | | | | | | | | | | | | | | | | | □ | | | | | | | | | | | | □ | | | | | | | | | | | | | | | | | | | | | □ | | | | | | |  |  |
| Hygiene training of relatives by nursing staff | | | | | □ | | | | | | | | | | □ | | | | | | | | | | | | | | | | | | | □ | | | | | | | | | | | | □ | | | | | | | | | | | | | | | | | | | | | □ | | | | | | |  |  |
| No farewell rituals | | | | | □ | | | | | | | | | | □ | | | | | | | | | | | | | | | | | | | □ | | | | | | | | | | | | □ | | | | | | | | | | | | | | | | | | | | | □ | | | | | | |  |  |
| 74. Do you have a written hygiene concept specifically for PCU?  If so, we would be pleased if you could send it to us for anonymous evaluation by e-mail to katja.maus[at:@]ukbonn.de.  If you would like to receive a reminder email after the survey, just let us know your e-mail address.  We will be very happy to get back to you! | | | | | | | | | | | | | | | | | | | | | | | | | | | | | | | | | | | | | | | | | | | | | | | | | | | | | | | | | | | | | | | | | | | | | | | | | |  |  |
| Please insert your e-mail address | | | | | | | | | | | | | | | | | | | | | | | | | | | | | | | | | | | | | | | | | | | | | | | | | | | | | | | | | | | | | | | | | | | | | | | | | |  |  |
|  | | | | | | | | | | | | | | | | | | | | | | | | | | | | | | | | | | | | | | | | | | | | | | | | | | | | | | | | | | | | | | | | | | | | | | | | | |  |  |
| Finally, we would like to ask 3 questions:  75. Approximately how many requests have you had in 2019 and 2020 | | | | | | | | | | | | | | | | | | | | | | | | | | | | | | | | | | | | | | | | | | | | | | | | | | | | | | | | | | | | | | | | | | | | | | | | | |  |  |
| 2019 ………………2020 | | | | | | | | | | | | | | | | | | | | | | | | | | | | | | | | | | | | | | | | | | | | | | | | | | | | | | | | | | | | | | | | | | | | | | | | | |  |  |
| 74. In which city and in which district is your palliative care unit located? | | | | | | | | | | | | | | | | | | | | | | | | | | | | | | | | | | | | | | | | | | | | | | | | | | | | | | | | | | | | | | | | | | | | | | | | | |  |  |
|  | | | | | | | | | | | | | | | | | | | | | | | | | | | | | | | | | | | | | | | | | | | | | | | | | | | | | | | | | | | | | | | | | | | | | | | | | |  |  |
| 75. What is your profession/position? | | | | | | | | | | | | | | | | | | | | | | | | | | | | | | | | | | | | | | | | | | | | | | | | | | | | | | | | | | | | | | | | | | | | | | | | | |  |  |
| □ Nurse  □ Head nurse  □ Physician, consultant  □ Head physician/head of department  □ Social worker  □ Psychologist  □ Admin  □ Other | | | | | | | | | | | | | | | | | | | | | | | | | | | | | | | | | | | | | | | | | | | | | | | | | | | | | | | | | | | | | | | | | | | | | | | | | |  |  |
