# Supplementary material for: National strategy for palliative care of severely ill and dying people and their relatives in pandemics (PallPan) in Germany - study protocol of a mixed-methods project
Source: BMC Palliat Care. 2022 Jan 13;21:10. doi: 10.1186/s12904-021-00898-w (PMC8756412; doi:10.1186/s12904-021-00898-w)
Supplement: Supplementary file 15 — Additional file 15: Supplementary file WP6. Interview guide Pandemic Teams. [file 12904_2021_898_MOESM15_ESM.docx]

**PallPan: "National strategy for palliative care in pandemic times" WP 6: Pandemic response teams Interview guide**

**Interview of pandemic response team members on the topic of palliative medicine during the pandemic**

**Interview guide**

| Date: | ___________________ |
| --- | --- |
| Interviewer: | ___________________ |
| Participant’s ID: | ___________________ |

*The interviews covered the composition of the pandemic response team and communication within it; its organisational structure and the nature and extent of its powers; personal and job-related information pertaining to the participants; the pandemic response team’s activities related to caring for seriously ill and dying patients with and without SARS-CoV-2 infection during pandemic restrictions; and care of bereaved family members.*

**A. Task in the pandemic response team (PRT)**

| Which PRT do you work in? |  |
| --- | --- |
| Is working in the PRT part of your main profession?  ● yes: since when? |  |
| How much time do you work for the PRT (hours worked per week/percent of weekly working hours) |  |

**B. Caring for seriously ill patients (± Covid-19), the dying and the bereaved**

1. First phase: Since WHO declared the pandemic (11 March 2020)
2. Second phase: Once the first restrictions were eased
3. Third phase: Case numbers currently on the rise again

**Focus of crisis management**

| Have you experienced different phases?  ● Different phases  ● Impact on work in the PRT |  |
| --- | --- |
| What are the main responsibilities of your PRT?  ● Changes as a result of transitioning from one phase to the next |  |
| In what order were topics dealt with?  ● Why? |  |
| Were death, dying and grief given consideration?  ● yes: in which phase? What exactly were you able to do/achieve?  ● no: did you feel that palliative care or similar topics ought to have been taken into account? |  |
| Did you keep any written record of recommendations/instructions?  yes: Could you provide us with the relevant documents? (letters, handouts, instructions, recommendations) |  |

**Members and structure of pandemic response team**

| **Only for macro and meso level:**  Are structures and responsibilities within your PRT governed by rules/regulations?  ● yes: which ones?  ● no: move to next question |  |
| --- | --- |
| Who is the initiator of your PRT? |  |
| Who was/is responsible for deciding on the members of the PRT?  ● Institution/function |  |
| How many members are there in your PRT? |  |
| Which specialist areas are represented in your PRT? |  |
| Are areas of responsibility within the PRT clearly defined?  ● yes: which ones? |  |
| Did your PRT include a regular/advisory member from a palliative or hospice care team?  ● yes: Which contribution was made by palliative experts? Which impulses did they give?    ● no: Would you have liked a palliative expert to be involved?  - incurable diseases  - existential questions  - dealing with death and dying  - ethical issues |  |
| Did you feel the lack of other areas of expertise?  ● yes: What would you have liked other experts to contribute? |  |
| **only for macro and meso level:**  Were specialist associations or other representative bodies involved in the work of your PRT?  e.g. Deutsche Gesellschaft für Palliativmedizin (German Association for Palliative Medicine) or the equivalent associations for pneumology or infectiology |  |

**Communication in the pandemic response team**

| Does communication take place online or in personal meetings? |  |
| --- | --- |
| How often are regular meetings held? |  |
| How do members of the PRT communicate with each other and with other departments?  - Taking feedback into account  - Interdisciplinary exchanges  - Interviewing staff |  |
| When taking decisions, were you able to refer to pandemic plans or other rules/regulations? |  |
| How does the PRT take decisions/set priorities?  - Majority decision  - Expert opinions  - Decisions are taken by leaders |  |
| Is there an ethic task force or a committee responsible for setting priorities/organising triage? |  |
| Which means of communication are used to make announcements or publish information about decisions?  - Internet forum, posters, flyers, notice board, e-mail |  |

**Structure and powers of the pandemic response team**

| Which higher authorities does the PRT report to? |  |
| --- | --- |
| What powers does your PRT have and what is the legal basis for them?  - Laws, ordinances |  |
| Which possibilities does the PRT have to enforce decisions?  ● for whom are decisions binding/not binding?  ● who monitors implementation? |  |
| What is the area of validity for resolutions/decisions passed by your PRT?  - all individual units/institutions run by the governing body |  |
| What is determined by supervisory authorities (term, powers)? |  |
| Has a maximum duration been decided for your PRT?  ● yes: Will the PRT structure be kept for future pandemics? |  |

**D. Personal details and professional experience**

1. Gender: □_1_ female □_2_ male □_3_ diverse
2. Age [years]: _____
3. Profession:

□_1_ medical profession

□_a_ doctor (specialty ________________)

□_b_ nurse/careworker

□_c_ other

□_2_ administrative profession

□_a_ politician

□_b_ office worker

□_c_ member of hospital board

□_d_ other

□_3_ helping profession

□_a_ pastoral care worker

□_b_ psychologist

□_c_ other

□_4_ economist

□_5_ other, ________________________________

1. Current workplace

Health care

□_1_ administration

□_2_ clinical department within a hospital (internal medicine, surgery, other)

□_3_ theoretical speciality (hygiene, microbiology, pathology)

□_4_ outpatient care/specialty:______________________

□_5_ other_____________________________

other

□_1_ administration

□_2_ municipal authorities

□_3_ Federal authorities

□_4_ European authorities

□_4_ other_____________________________

1. How long have you worked in this profession?

□_1_ less than one year

□_2_between 1 and 5 years

□_3_ between 5 and 10 years

□_4_ more than 10 years

1. Do you have a managerial position?
2. How many members of staff are you responsible for?

□_1_ fewer than 10

□_2_between 10 and 50

□_3_ between 51 and 100

□_4_ more than 100

8. Town/country

9. Highest seven day incidence rate per 100 days/100000 citizens

10. Governing body: run privately, by the church or by the state
